# Maintenance proton pump inhibitor use and risk of colorectal cancer: a Swedish retrospective cohort study

**DOI:** 10.1136/bmjopen-2023-079591

**Published:** 2024-07-02

**Authors:** Qing Liu, Xinchen Wang, Lars Engstrand, Omid Sadr-Azodi, Katja Fall, Nele Brusselaers

**Affiliations:** 1 Department of Microbiology, Tumour and Cell Biology, Karolinska Institutet, Stockholm, Sweden; 2 Centre for Psychiatry Research, Department of Clinical Neuroscience, Karolinska Institutet, Stockholm, Sweden; 3 Department of Clinical Sciences, Intervention and Technology, Karolinska Institutet, Stockholm, Sweden; 4 Department of Surgery, Capio Saint Göran Hospital, Stockholm, Sweden; 5 Clinical Epidemiology and Biostatistics School of Medical Sciences, Örebro University, Örebro, Sweden; 6 Institute of Environmental Medicine, Karolinska Institutet, Stockholm, Sweden; 7 Department of Head and Skin, Ghent University, Ghent, Belgium; 8 Global Health Institute, Antwerp University, Antwerp, Belgium

**Keywords:** CLINICAL PHARMACOLOGY, EPIDEMIOLOGIC STUDIES, GASTROENTEROLOGY, Gastrointestinal tumours, REGISTRIES, Risk Factors

## Abstract

**Objectives:**

We aimed to evaluate the risk of colorectal adenocarcinoma (CRA) associated with long-term use of proton pump inhibitors (PPIs) in a large nationwide cohort.

**Design:**

Retrospective cohort study.

**Setting:**

This research was conducted at the national level, encompassing the entire population of Sweden.

**Participants:**

This study utilised Swedish national registries to identify all adults who had ≥180 days of cumulative PPI use between July 2005 and December 2012, excluding participants who were followed up for less than 1 year. A total of 754 118 maintenance PPI users were included, with a maximum follow-up of 7.5 years.

**Interventions:**

Maintenance PPI use (cumulative≥180 days), with a comparator of maintenance histamine-2 receptor antagonist (H_2_RA) use.

**Primary and secondary outcome measures:**

The primary outcome measure was the risk of CRA, presented as standardised incidence ratios (SIRs) with 95% confidence intervals (CIs). Subgroup analyses were performed to explore the impact of indications, tumour locations, tumour stages and the duration of follow-up. A multivariable Poisson regression model was fitted to estimate the incidence rate ratios (IRRs) and 95% CIs of PPI versus H_2_RA use.

**Results:**

Maintenance PPI users exhibited a slightly elevated risk of CRA compared to the general population (SIR 1.10, 95% CI=1.06 to 1.13) for both men and women. Individuals aged 18–39 (SIR 2.79, 95% CI=1.62 to 4.47) and 40–49 (SIR 2.02, 95% CI=1.65 to 2.45) had significantly higher risks than the general population. Right-sided CRA showed a higher risk compared to the general population (SIR 1.26, 95% CI=1.20 to 1.32). There was no significant difference in the risk of CRA between maintenance PPI users and maintenance H_2_RA users (IRR 1.05, 95% CI=0.87 to 1.27, *p*<0.05).

**Conclusions:**

Maintenance PPI use may be associated with an increased risk of CRA, but a prolonged observation time is needed.

**STRENGTHS AND LIMITATIONS OF THIS STUDY**

- High number of enrolled individuals and person-years.
- High external validity and reduced residual confounding by the active comparator.
- Lack of additional data on some lifestyle risk factors.
- Potential confounding introduced by ever users who received maintenance proton pump inhibitor use long before the study period.
- Longer follow-up (ie, more than 10 years) may be needed.

## Introduction

Proton pump inhibitors (PPIs) are one of the most commonly prescribed drugs globally and are widely used for the treatment and prevention of various gastric acid-related disorders.^1 2^ The use of both prescription and over-the-counter PPIs has increased in many countries, with an estimated 10%–30% of adults regularly using PPIs.^3 4^ Although PPIs have been deemed safe for short-term use and are recommended for a broad spectrum of indications by current guidelines and expert reviews,^5–7^ only a few indications justify long-term use exceeding 4 weeks. Despite this, PPIs are often overprescribed,^7 8^ which highlights the need for further investigation into the potential long-term side effects and public health implications of their widespread use.

Long-term PPI use has been associated with an increased risk of gastrointestinal cancers, including gastric, oesophageal and pancreatic cancers.^9–11^ Previous studies have demonstrated that PPI use significantly impacts the gut microbiome and contributes to dysbiosis,^12–14^ which has been linked to oncogenesis in the gut epithelium.^15 16^ Enteric infections and the trophic effects of PPI-induced hypergastrinaemia may also serve as potential cancerogenic factors.^17^ While some cohort studies have reported elevated relative risks of colorectal cancer (CRC) following PPI use,^18 19^ others have found no association between CRC and PPI use.^2 20–25^ However, potential methodological limitations in some of these studies have impacted the results. For example, some case–control studies introduced selection bias in exposure effect estimation by enrolling prevalent users rather than new users,^21 22 24 26^ and latency bias and time-window bias were present in some previous cohort studies due to the selection of narrow observation time ranges.^19 21 27^

To clarify the relationship between PPI use and CRC, we conducted a large cohort study using high-coverage national registries to control for immortal time bias and time-window bias. We aimed to assess the association between maintenance PPI use and the risk of colorectal adenocarcinoma (CRA), which accounts for >95% of all CRC cases.^28^ We focused on right-sided and left-sided CRAs due to their chronological exposure to the drug-effects based on the anatomical locations.^28 29^ Additionally, we used the less popular histamine-2 receptor antagonists (H_2_RAs) as active comparators in the supplementary analysis to minimise confounding-by-indication, as they share similar indications with PPIs and were used for treating gastric acid-related disorders before the introduction of PPIs as more potent acid suppressants.^30^

## Methods

### Study design

This was a register-based cohort study that enrolled all Swedish adults (aged 18 years old) who were on maintenance PPI therapy between July 2005 and December 2012. The study population was compared to the general population (ie, the Swedish total population) stratified by the same age, sex and calendar period. Participants were followed from 1 year after the first dispensed prescription date until the first diagnosis of CRA, death or the end of the study period (31 December 2012). Data were obtained from the Swedish Cancer Registry, the National Patient Registry, the Cause of Death Registry, the Prescribed Drug Registry and the Swedish Total Population Registry.^31–35^ Cancer and demographic information of the total population can be downloaded from the National Board of Health and Welfare’s statistical database.^31^ The study protocol was established a priori, and the study was conducted in accordance with the Strengthening the Reporting of Observational Studies in Epidemiology (STROBE) statement.^36^

### Patient and public involvement

Due to its data-driven nature, this retrospective study had no patient or public directly involved in the study’s design, data analysis, method selection or dissemination planning. Research questions and outcome measures were derived from existing literature and clinical relevance without direct input from them. Recruitment and follow-up relied on existing records from national registries.

### Exposure

PPI usage data were obtained from the Prescribed Drug Registry, labelled by the anatomical therapeutic chemical (ATC)/defined daily dose (DDD) system. This system annotated the DDD per prescribed package with ATC codes. PPIs are also available over the counter in Sweden but only in small quantities and at exorbitant prices in comparison to prescribed PPIs.^37^ Therefore, most PPI consumption of long-term users is likely recorded in this registry. Maintenance PPI use (ATC code A02BC) was defined as cumulative use for at least 180 days prior to the onset of any cancer, death, or the end of the study period. Maintenance H_2_RA (ATC code A02BA) use served as an active comparator, and it was defined as using H_2_RA for at least 180 days before the onset of any cancer, death, or the end of the study period.

## Outcome

The primary outcome was CRA, defined using the International Classification of Diseases (ICD, 10th edition) codes C18–C20 from the Swedish Cancer Registry,^38^ and the histopathology code 096. The tumours were further classified based on their anatomical location as right-sided, left-sided or overlapping/unspecified sites (ICD codes in online supplemental table S1). The tumour-node-metastasis (TNM) classification of the American Joint Committee on Cancer eighth edition Staging of Colorectal Cancer was used to define tumour stage and grouped as follows: stages 0–I (Tis, N0, M0 for stage 0; T1–2, N0, M0 for stage I), II (T3–4, N0, M0), III (T1–4, N1–2, M0) and IV (any T, any N, M1).^39^

10.1136/bmjopen-2023-079591.supp1 Supplementary data

### Exclusions

Individuals with any documented cancer diagnosis prior to the study period or prior to the first dispensing date of PPIs were excluded. Individuals who switched between maintenance use of PPIs and H_2_RAs (defined as ≥180 days of accumulated use of either drug) or had an exposure period of less than 1 year were also excluded. (online supplemental figure S1)

### Covariates

Age at treatment initiation was categorised into 18–39, 40–49, 50–59, 60–69, and ≥70 years old, and calendar periods were categorised into 2005–2006, 2007–2009, and 2010–2012. The duration of time since drug initiation was categorised as 1–2, 2–4, 4–6, and ≥6 years. No missing values for these variables were found for any of the study participants.

The most common recorded indications of maintenance PPI use were extracted from the National Patient Registry, including *Helicobacter pylori* infection or eradication, gastro-oesophageal reflux, Barrett’s oesophagus, peptic ulcer, Zollinger Ellison’s syndrome, gastro-duodenitis, dyspepsia, maintenance use (>180 days during the study period) of low-dose aspirin, and maintenance use of non-steroidal anti-inflammatory drugs (NSAIDs)^9 11 40^ (ICD codes in online supplemental table S1). We performed subgroup analyses on individuals with and without the listed indications, as well as on each indication separately when the statistical power was sufficient.

### Statistical analyses

We calculated standardised incidence ratios (SIRs) with 95% confidence intervals (CIs) to assess the association between maintenance PPI use and CRA. Standardisation was performed according to Breslow and Day’s method, and the allocation of person-years followed Clayton’s algorithm.^41^ SIRs were obtained by calculating the ratio of observed CRA incidence rate compared to the expected incidence rate derived from the incidence among the Swedish total population of the same age, sex and calendar period. The information on the incidence of CRA (summarised in online supplemental table S2) and the demographic characteristics of the total population can be downloaded from the National Board of Health and Welfare’s statistical database.^42^ During the study period (2005–2012), the overall incidence of CRA in men ranged between 81.8 - 89.9/100 000 individuals and between 74.8 - 79.7/100 000 individuals in women (presented in online supplemental figure 2). In our main analysis, we compared the overall risk of CRA in the study population to that in the general population of Sweden, stratifying the main outcomes by tumour stages (0–I, II, III, IV, and missing values), tumour location (right-sided and left-sided), indications and follow-up time since drug initiation (1–2, 2–4, 4–6, and ≥6 years). We extensively investigated the risk of different tumour locations by age, sex, and indications for maintenance PPI use. Additionally, we assessed the risk of CRA in maintenance PPI users with a maximal follow-up time from the first dispensed prescription date, considering age, sex, calendar period, tumour stage and tumour location.

Furthermore, we conducted two supplementary analyses. First, we estimated SIRs for maintenance H_2_RA users and conducted a duration analysis (1–2, 2–4, 4–6, and ≥6 years). Second, we used a multivariable Poisson regression model to estimate incidence rate ratios (IRRs) with 95% CIs to compare CRA risk between maintenance PPI users and H_2_RA users, adjusting for age, sex, and common therapy indications.^43^ The number of patients analysed for the Poisson regression model equals the summarised number of maintenance PPI users and maintenance H_2_RA users. Propensity score adjustment between maintenance PPI users and H_2_RA users was performed in the model to better control for confounders. Propensity scores were estimated using multivariable logistic regression, including age, sex, and indications. We applied *p* values less than 0.05 statistically significant for all the analyses. Stata MP V.14.2 was used for all calculations.

## Results

### Characteristics of the PPI cohort

This study included 754 118 adult maintenance PPI users with no prior history of cancer, with a median follow-up of 5.3 years (totalling 4 177 396 person-years) and a maximum follow-up of 7.5 years. Women (0.52%) had a lower cumulative incidence of CRA than men (0.62%). Maintenance PPI use was most common in individuals aged 60–69 and ≥70 years old (tables 1 and 2). A total of 4432 cases of CRA were identified, with the majority being left-sided (n=2499, 56.4%), stages II (n=1102, 24.9%) and III (n=1017, 22.9%).

**Table 1:** Characteristics of all maintenance proton pump inhibitor (PPI) users in Sweden between 2005 and 2012

Characteristics of the study population	Maintenance PPI users	
	Number	%
Total	754 118	100.0
Age at starting the maintenance PPI use, years		
18–39 years	84 510	11.2
40–49 years	99 897	13.3
50–59 years	149 167	19.8
60–69 years	167 456	22.2
≥70 years	253 088	33.6
Sex		
Men	310 357	41.2
Women	443 761	58.9
Calendar period		
2005–2006	430 527	57.1
2007–2009	221 369	29.4
2010–2012	102 222	13.6
Colorectal adenocarcinoma	4432	0.6
Right-sided	1884	0.3
Left-sided	2499	0.3
Overlapping or unspecified	49	0.0
Tumour stages of colorectal adenocarcinoma		
Stages 0–I	674	0.1
Stage II	1102	0.1
Stage III	1017	0.1
Stage IV	748	0.1
Missing	891	0.1
Indications for maintenance PPI use		
*Helicobacter pylori* infection or eradication	55 311	7.3
Gastro-oesophageal reflux	193 371	25.6
Barrett’s oesophagus	5717	0.8
Peptic ulcer	74 946	10.0
Zollinger Ellison’s syndrome	28	0.0
Gastro-duodenitis	100 191	13.2
Dyspepsia	41 916	5.6
Aspirin maintenance use	262 242	34.8
Non-steroidal anti-inflammatory drugs maintenance use	231 792	30.7
Without any of these recorded indications	186 529	24.7
Maintenance PPI use based on daily drug usage		
IQR	1810	–
Person-years	3 426 925	–
Follow-up time, years (median)	5.3	–

IQR, interquartile range; PPI, proton pump inhibitor.

**Table 2:** Risk of colorectal adenocarcinomas in maintenance proton pump inhibitor (PPI) users, expressed as standardised incidence ratios (SIRs) and 95% confidence intervals (CIs) by age, sex, calendar period, tumour subsites, and stages

	Maintenance PPI users	
	Number	SIR (95% CI)
Total colorectal adenocarcinomas	4432	1.10 (1.06 to 1.13)
Sex		
Men	2113	1.13 (1.08 to 1.18)
Women	2319	1.10 (1.06 to 1.15)
Age at starting the maintenance PPI use, years		
18–39	38	2.79 (1.62 to 4.47)
40–49	145	2.02 (1.65 to 2.45)
50–59	531	1.27 (1.13 to 1.41)
60–69	1277	1.26 (1.19 to 1.34)
≥70	2441	1.04 (1.00 to 1.07)
Calendar year period		
2005–2006	3215	1.29 (1.09 to 1.51)
2007–2009	1049	1.17 (1.12 to 1.22)
2010–2012	168	1.07 (1.02 to 1.11)
Subsites of colorectal adenocarcinoma		
Right-sided	1884	1.26 (1.20 to 1.32)
Left-sided	2499	0.99 (0.96 to 1.03)
Stages of colorectal adenocarcinoma		
Stages 0–I	674	1.22 (1.13 to 1.32)
Stage II	1102	1.10 (1.03 to 1.16)
Stage III	1017	1.07 (1.00 to 1.13)
Stage IV	748	0.95 (0.88 to 1.02)
Missing	891	1.19 (1.11 to 1.27)

CI, confidence interval; PPI, proton pump inhibitor; SIR, standardised incidence ratio.

### Risk of colorectal adenocarcinoma

Compared to the general population, the overall risk of CRA was increased in maintenance PPI users (SIR 1.10, 95% CI 1.06 to 1.13). This risk was particularly affected by age, with the highest estimates present in those aged 18–39 years (SIR 2.79, 95% CI 1.62 to 4.47) and 40–49 years (SIR 2.02, 95% CI 1.65 to 2.45), while no convincing association was found in individuals ≥70 years old (SIR 1.04, 95% CI 1.00 to 1.07). In maintenance PPI users, the diagnosed CRA was particularly associated with an increased risk of early stage (stages 0–I, SIR 1.12, 95% CI 1.04 to 1.21) (table 2). In terms of tumour location, maintenance PPI users had a higher risk of right-sided CRA than the general population, regardless of age and sex (tables 2 and 3). Meanwhile, no association between PPI use and CRA risk was found in left-sided CRC (tables 2 and 3).

**Table 3:** Sex and age disparities in the risk of right-sided and left-sided colorectal adenocarcinomas in maintenance proton pump inhibitor (PPI) users, expressed as standardised incidence ratios (SIRs) and 95% confidence intervals (CIs)

Cancer in categories	Right-sided colorectal adenocarcinoma		Left-sided colorectal adenocarcinoma	
	Number	SIR (95% CI)	Number	SIR (95% CI)
Sex				
Men	736	1.25 (1.16 to 1.34)	1359	1.04 (0.98 to 1.10)
Women	1148	1.27 (1.19 to 1.34)	1,14	0.94 (0.89 to 1.00)
Age at starting the maintenance PPI, years				
18–39	13	2.78 (1.02 to 6.06)	24	2.81 (1.40 to 5.03)
40–49	37	1.98 (1.31 to 2.89)	103	1.95 (1.53 to 2.46)
50–59	167	1.42 (1.14 to 1.75)	359	1.20 (1.05 to 1.37)
60–69	528	1.58 (1.43 to 1.75)	732	1.12 (1.03 to 1.21)
≥70	1139	1.17 (1.11 to 1.24)	1281	0.90 (0.86 to 0.95)

CI, confidence interval; PPI, proton pump inhibitor; SIRs, standardised incidence ratios.

### Common indications for maintenance PPI use

Indications for maintenance PPI use did not show the same trend for the drug–cancer–risk relationship in the exposed population. PPI use was not related to an increased risk of CRA among patients with Barrett’s oesophagus. A marginally increased risk of CRA among PPI users was observed in participants with the indications of *Helicobacter pylori* infection or eradication (SIR 1.29, 95% CI 1.15 to 1.44), dyspepsia (SIR 1.29, 95% CI 1.16 to 1.42), gastro-duodenitis (SIR 1.28, 95% CI 1.19 to 1.38), gastro-oesophageal reflux (SIR 1.26, 95% CI 1.19 to 1.33) and peptic ulcer (SIR 1.16, 95% CI 1.07 to 1.26). Among individuals with the combined use of non-steroidal anti-inflammatory drugs (NSAIDs) or low-dose aspirin, maintenance PPI use was not significantly associated with the risk of CRA (table 4). Compared to the general population, most of the indications were related to an increased risk of right-sided CRAs in maintenance PPI users (table 4).

**Table 4:** Risk of (a) colorectal adenocarcinomas (b) right-sided and left-sided colorectal adenocarcinomas according to indications for maintenance proton pump inhibitor (PPI) users, expressed as standardised incidence ratios (SIRs) and 95% confidence intervals (CIs)

(a) Risk of colorectal adenocarcinomas		
Indications for maintenance of PPI use	Colorectal adenocarcinoma	
	Number of cases (% in maintenance PPI users)	SIR (95% CI)
Without any below indication	879 (4.7)	1.13 (1.06 to 1.21)
*Helicobacter pylori* infection or eradication	328 (5.9)	1.29 (1.15 to 1.44)
Gastro-oesophageal reflux	1352 (7)	1.26 (1.19 to 1.33)
Barrett’s oesophagus	39 (6.8)	1.16 (0.83 to 1.59)
Peptic ulcer	625 (8.3)	1.16 (1.07 to 1.26)
Zollinger Ellison’s syndrome	0	–
Gastro-duodenitis	739 (7.4)	1.28 (1.19 to 1.38)
Dyspepsia	368 (8.8)	1.29 (1.16 to 1.42)
Maintenance use of low-dose aspirin	2026 (7.7)	1.00 (0.96 to 1.05)
Maintenance use of non-steroidal anti-inflammatory drugs	1096 (4.7)	1.00 (0.94 to 1.06)
With any of the above indications	3553 (6.3)	1.09 (1.05 to 1.12)

(b) Risk of right-sided and left-sided colorectal adenocarcinomas				
Indications for maintenance of PPI use	Right-sided colorectal adenocarcinoma		Left-sided colorectal adenocarcinoma	
	Number of cases (% in maintenance PPI users)	SIR (95 % CI)	Number of cases (% in maintenance PPI users)	SIR (95 % CI)
Without any below indication	360 (1.9)	1.27 (1.15 to 1.41)	511 (2.7)	1.05 (0.97 to 1.15)
*Helicobacter pylori* infection or eradication	148 (2.7)	1.63 (1.37 to 1.91)	176 (3.2)	1.09 (0.94 to 1.27)
Gastro-oesophageal reflux	616 (3.2)	1.58 (1.45 to 1.70)	718 (3.7)	1.06 (0.99 to 1.15)
Barrett’s oesophagus	17 (3)	1.53 (0.89 to 2.45)	21 (3.7)	0.95 (0.59 to 1.45)
Peptic ulcer	288 (3.8)	1.45 (1.28 to 1.62)	333 (4.4)	1.00 (0.90 to 1.11)
Zollinger Ellison’s syndrome	0	–	0	
Gastro-duodenitis	361 (3.6)	1.70 (1.53 to 1.88)	370 (3.7)	1.03 (0.93 to 1.14)
Dyspepsia	186 (4.4)	1.74 (1.50 to 2.01)	179 (4.3)	1.01 (0.87 to 1.17)
Maintenance use of low-dose aspirin	900 (3.4)	1.18 (1.10 to 1.26)	1110 (4.2)	0.90 (0.85 to 0.95)
Maintenance use of non-steroidal anti-inflammatory drugs	397 (1.7)	0.98 (0.89 to 1.09)	685 (3)	1.00 (0.92 to 1.08)
With any of the above indications	1524 (2.7)	1.26 (1.19 to 1.32)	1988 (3.5)	0.98 (0.94 to 1.02)

CI, confidence interval; PPI, proton pump inhibitor; SIRs, standardised incidence ratios.

### Duration of follow-up time

**Figure 1:**
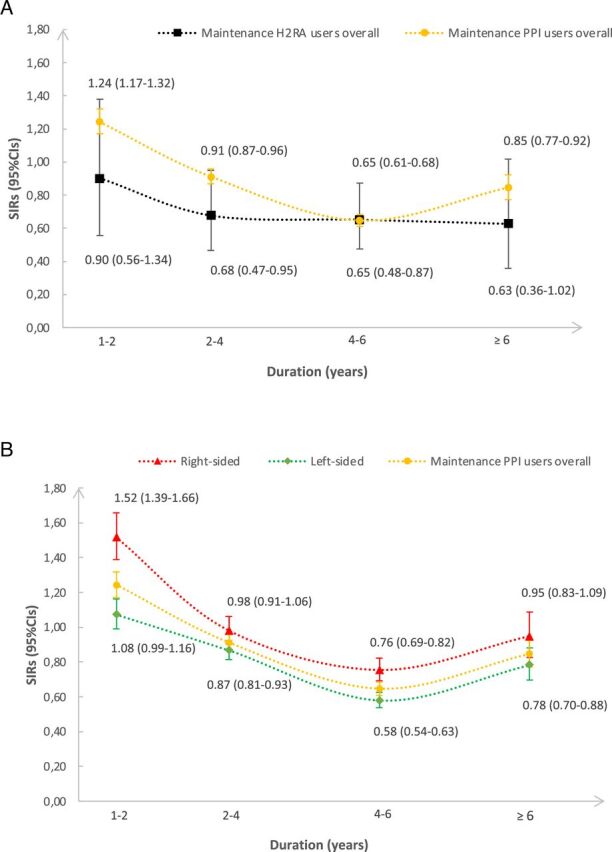
Duration of follow-up and risk of colorectal adenocarcinomas among (A)maintenance proton pump inhibitor (PPI) users and maintenance histamine-2 receptor antagonist (H_2_RA) users (B)overall maintenance PPI users and users diagnosed with right-sided or left-sided colorectal adenocarcinomas, expressed as standardised incidence ratios (SIRs) and 95% confidence intervals (CIs).

There was an elevated risk of CRA observed between the exposed population and the general population during the 1–2 years of follow-up (SIR 1.24, 95% CI 1.17 to 1.32). Thereafter, the results suggested slightly decreased risks of CRA in the 2–4 years (SIR 0.91, 95% CI 0.87 to 0.96), 4–6 years (SIR 0.65, 95% CI 0.61 to 0.68), and ≥6 years (SIR 0.85, 95% CI 0.77 to 0.92) of follow-up (figure 1A). There was no obvious difference in CRA risks observed between the maintenance H_2_RA users and the general population during the follow-up time (figure 1A). An increased risk of right-sided CRA was also observed during the 1–2 years of follow-up (figure 1B).

### Comparison with maintenance H_2_RA use

With a total of 19 795 individuals and 130 810 person-years of follow-up, the risk of CRA was not found to be associated with maintenance H_2_RA use compared to the background population (n=115, SIR 0.85, 95% CI 0.71 to 1.03). The multivariable Poisson regression model of maintenance PPI versus H_2_RA use showed an IRR of 1.05 (95% CI 0.87 to 1.27, *p*<0.05).

## Discussion

In this large population-based study investigating the association between maintenance PPI use and CRC risk, we found a potential 10% higher risk of CRA compared to the general population. SIRs showed that maintenance PPI use might be associated with an increased risk of right-sided CRA compared to that in the general population. Young to middle-aged adult (ie, 18–39 and 40–49 years old) participants showed a higher risk of CRA than the general population.

Our findings are in line with an estimated exposure effect of 2.03 (95% CI 1.56 to 2.63) between PPI users and CRC risk reported by a Taiwanese cohort,^18^ where immortal time bias could not be ruled out. In our study, we excluded 41 697 individuals with less than 1 year of follow-up to avoid detection bias. As CRA typically takes several to 20 years to develop from polyp to adenocarcinoma,^44^ cancer diagnoses or deaths within 1 year after new incident PPI prescriptions should be excluded because they could result in a biological effect indicating that PPI initiation preceded or increased the cancer risks. We have further carefully addressed the possibility that our strict exclusion of the first year of follow-up introduced immortal time bias. Therefore, we estimated the overall risk of CRA within the same study population starting follow-up from the first dispensed prescription date of PPIs. The results were consistent with our main analysis (online supplemental table S3), suggesting that immortal time bias might not be a problem; otherwise, the overestimates of healthy individuals should have led to an opposite observed effect.

In the duration analysis, maintenance PPI use was associated with an increased risk of CRA than the general population during the 1-2 years of follow-up. Following that the exposed population showed a slightly lower risk of CRA than the general population. Then, the exposed population had a lower but less decreased risk of CRA compared to the general population with ≥6 years of follow-up. A similar pattern was observed for the risk of right-sided CRA. This may be because of potential confounding introduced by ever users who received maintenance PPI use long before the study period, as we do not have information on prescribed drug use prior to the start of the study (July 2005) when the National Prescribed Drug Registry was not fully constructed. A longer follow-up (ie, more than 10 years) and more extensive wash-out of ever users may be needed, so that an increasing risk of CRA would be observed. Meanwhile, reverse causality (ie, the observed effect explained by reversely linking initiating PPI to subclinical symptoms of CRC) should also be considered during the follow-up. If CRC patients were prone to be prescribed maintenance-used PPIs, substantial positive effects on the exposure estimation might have influenced each stratum of the exposed population during our analysis. However, the different trends of right-sided versus left-sided CRC risk during different time periods (figure 1B) and with different indications (table 4) indicated that the reverse causality due to the prevalent use of maintenance PPIs might be less concerning.

Among the indication subgroup analyses, only combined maintenance PPI use with NSAIDs or low-dose aspirin showed a potential preventive effect against colorectal tumour progression. A previous American cohort study concluded that long-term use of NSAIDs and low-dose aspirin reduced the risk of CRC.^45^ Our study also provided evidence that long-term NSAID or low-dose aspirin use appeared to be beneficial in concomitant use with PPIs. While we acknowledged the existing literature of concomitant drug use with PPIs, the necessity for continued evaluation in light of the increased focus on combined PPI use with NSAIDs or low-dose aspirin still needs to be emphasised in assessing CRC preventive potential. Nevertheless, an increased risk of CRA was observed among the *Helicobacter pylori* infection or eradication, gastro-oesophageal reflux, gastro-duodenitis and dyspepsia groups, which have not been established as the risk factors for CRA. The elevated risk could be explained by the fact that acid-related disorders facilitate changes in the upper-intestinal microbiome barrier owing to gastric acid over secretion.^46 47^ Gut dysbiosis and hypergastrinaemia may also contribute to the association between PPI use and colorectal carcinogenesis.^15 48 49^

The strengths of our study include not only the high number of individuals and person-years, but also the well-designed method for calculating cancer risks. The high-quality, high-coverage national registries facilitated the identification of eligible individuals with a lengthy follow-up. Compared with previous studies, we constructed this cohort with many individuals and long follow-up times to minimise time-related bias. Moreover, the results are generalisable to other Nordic nations because comparable recording systems are maintained in other Nordic nations. The method enables us to compare large populations at different levels without considering migrations and drop-offs. In addition, we accounted for an alternative indicator of H_2_RA to reduce the residual confounding caused by PPI indications, which could lead to bidirectional results in the association between exposure and outcome. H_2_RAs are also used as acid suppressants to treat stomach acid production conditions like peptic ulcers and Zollinger-Ellison syndrome, and provide relief from excessive acid secretion symptoms.^50^ Similar to PPI, prolonged use of H_2_RAs can lead to elevated systemic levels of gastrin, a hormone known to stimulate the proliferation of colorectal epithelium and contribute to colon adenoma progression.^50 51^ Yet, our study showed that H_2_RAs may not be associated with CRA/CRC risks, in accordance with previous population-based studies.^21 52^ Thus, the residual confounding by indication was minimised when comparing maintenance PPI and maintenance H_2_RA use.

Our study has certain limitations, including a lack of additional data on lifestyle risk factors such as smoking, alcohol, diet, and body mass index. Moreover, although we followed the participants up to a maximum duration of 7.5 years, a median follow-up time of 5.3 years may be insufficient. A longer median follow-up permits a more extended observation of all naturally evoked outcomes and a more extensive exclusion of prevalent use without harming the sample size and power too much. Nevertheless, during the long progression period, the clinical diagnosis of CRA can be delayed following the onset of cancerous symptoms, which will dilute the association between the PPI and CRC risk.

In our study, maintenance PPI users account for less than 11% of the Swedish population (7.1–7.6 million people).^53 54^ The prevalence is lower than that of other Nordic countries, such as 15.5% in Denmark.^3^ But inappropriate PPI prescriptions are increasing among adults.^55 56^ Meanwhile, early onset CRC (diagnosed at <50 years of age) has been reported to be rising, especially as young populations at increased risk of CRC tend to have an unhealthy diet, a lack of exercise, obesity and a low acceptance of colonoscopy.^57–59^ The message to clinics is that young to middle-aged people should pay attention to the potential risk of CRA associated with maintenance PPI use. Moreover, combining low-dose aspirin or other NSAIDs with maintenance PPI treatment could be meaningful to PPI users with common indications, although further studies are needed before any firm conclusions can be made. Finally, right-sided CRA is more clinically insidious, more advanced at diagnosis and worse in prognosis than left-sided CRA.^29 60 61^ Early chemoprevention for maintenance PPI users should be considered to prevent poorer outcomes of right-sided CRA because of the significantly increased risk of right-sided CRA following maintenance PPI use observed in our study than the general population.

In conclusion, our study showed that maintenance PPI use may be associated with an increased risk of CRA. Nevertheless, we need more population-based studies with extended observation to confirm the association.

## Supplementary Material

Reviewer comments

Author's manuscript

## Data Availability

Data are available upon reasonable request. The data sets analysed during the current study are not publicly available due to the sharing agreement at Karolinska Institutet and the National Board of Health and Welfare but are available from the corresponding authors on reasonable request.

## References

[1] Shin JM , Sachs G . Pharmacology of proton pump inhibitors. Curr Gastroenterol Rep 2008;10:528–34. 10.1007/s11894-008-0098-4 19006606 PMC2855237

[2] Yang YX , Hennessy S , Propert K , et al . Chronic proton pump inhibitor therapy and the risk of colorectal cancer. Gastroenterology 2007;133:748–54. 10.1053/j.gastro.2007.06.022 17678926

[3] Hálfdánarson ÓÖ , Pottegård A , Björnsson ES , et al . Proton-pump inhibitors among adults: a nationwide drug-utilization study. Therap Adv Gastroenterol 2018;11. 10.1177/1756284818777943 PMC5977421 29872455

[4] Lassalle M , Le Tri T , Bardou M , et al . Use of proton pump inhibitors in adults in France: a nationwide drug utilization study. Eur J Clin Pharmacol 2020;76:449–57. 10.1007/s00228-019-02810-1 31838548

[5] Freedberg DE , Kim LS , Yang YX . The risks and benefits of long-term use of proton pump inhibitors: expert review and best practice advice from the American Gastroenterological Association. Gastroenterology 2017;152:706–15. 10.1053/j.gastro.2017.01.031 28257716

[6] Katz PO , Gerson LB , Vela MF . Guidelines for the diagnosis and management of gastroesophageal reflux disease. Am J Gastroenterol 2013;108:308–28. 10.1038/ajg.2012.444 23419381

[7] Targownik LE , Fisher DA , Saini SD . AGA clinical practice update on de-prescribing of proton pump inhibitors: expert review. Gastroenterology 2022;162:1334–42. 10.1053/j.gastro.2021.12.247 35183361

[8] Kurlander JE , Gonzalez JJ , Saini SD . Deprescribing proton pump inhibitors. JAMA Intern Med 2020;180:1711–2. 10.1001/jamainternmed.2020.2441 32804191

[9] Brusselaers N , Engstrand L , Lagergren J . Maintenance proton pump inhibition therapy and risk of oesophageal cancer. Cancer Epidemiology 2018;53:172–7. 10.1016/j.canep.2018.02.004 29477057

[10] Schneider JL , Kolitsopoulos F , Corley DA . Risk of gastric cancer, gastrointestinal cancers and other cancers: a comparison of treatment with Pantoprazole and other proton pump inhibitors. Aliment Pharmacol Ther 2016;43:73–82. 10.1111/apt.13450 26541643

[11] Brusselaers N , Sadr-Azodi O , Engstrand L . Long-term proton pump inhibitor usage and the association with Pancreatic cancer in Sweden. J Gastroenterol 2020;55:453–61. 10.1007/s00535-019-01652-z 31811561 PMC7080689

[12] Imhann F , Bonder MJ , Vich Vila A , et al . Proton pump inhibitors affect the gut microbiome. Gut 2016;65:740–8. 10.1136/gutjnl-2015-310376 26657899 PMC4853569

[13] Vich Vila A , Collij V , Sanna S , et al . Impact of commonly used drugs on the composition and metabolic function of the gut microbiota. Nat Commun 2020;11:362:362. 10.1038/s41467-019-14177-z 31953381 PMC6969170

[14] Dekkers KF , Sayols-Baixeras S , Baldanzi G , et al . An online atlas of human plasma metabolite signatures of gut microbiome composition. Nat Commun 2022;13:5370. 10.1038/s41467-022-33050-0 36151114 PMC9508139

[15] Bruno G , Zaccari P , Rocco G , et al . Proton pump inhibitors and dysbiosis: current knowledge and aspects to be clarified. World J Gastroenterol 2019;25:2706–19. 10.3748/wjg.v25.i22.2706 31235994 PMC6580352

[16] Chen CC , Liou JM , Lee YC , et al . The interplay between helicobacter pylori and gastrointestinal microbiota . Gut Microbes 2021;13. 10.1080/19490976.2021.1909459 PMC8096336 33938378

[17] Srivastava A , Gupta J , Kumar S , et al . Gut biofilm forming bacteria in inflammatory bowel disease. Microbial Pathogenesis 2017;112:5–14. 10.1016/j.micpath.2017.09.041 28942174

[18] Lei W-Y , Wang J-H , Yi C-H , et al . Association between use of proton pump inhibitors and colorectal cancer: a nationwide population-based study. Clin Res Hepatol Gastroenterol 2021;45. 10.1016/j.clinre.2020.02.017 32224118

[19] Lai SW , Liao KF , Lai HC , et al . Use of proton pump inhibitors correlates with increased risk of colorectal cancer in Taiwan. Asia Pac J Clin Oncol 2013;9:192–3. 10.1111/ajco.12054 23298363

[20] Robertson DJ , Larsson H , Friis S , et al . Proton pump inhibitor use and risk of colorectal cancer: a population-based, case-control study. Gastroenterology 2007;133:755–60. 10.1053/j.gastro.2007.06.014 17678921

[21] Chubak J , Boudreau DM , Rulyak SJ , et al . Colorectal cancer risk in relation to use of acid suppressive medications. Pharmacoepidemiol Drug Saf 2009;18:540–4. 10.1002/pds.1749 19367565 PMC3690626

[22] van Soest EM , van Rossum LGM , Dieleman JP , et al . Proton pump inhibitors and the risk of colorectal cancer. Am J Gastroenterology 2008;103:966–73. 10.1111/j.1572-0241.2007.01665.x 18070237

[23] Lee JK , Merchant SA , Schneider JL , et al . Proton pump inhibitor use and risk of gastric, colorectal, liver, and pancreatic cancers in a community-based population. Am J Gastroenterol 2020;115:706–15. 10.14309/ajg.0000000000000591 32205645

[24] Kuiper JG , Herk‐Sukel MPP , Lemmens V , et al . Proton pump inhibitors are not associated with an increased risk of colorectal cancer. GastroHep 2020;2:165–70. 10.1002/ygh2.409

[25] Abrahami D , McDonald EG , Schnitzer ME , et al . Proton pump inhibitors and risk of colorectal cancer. Gut 2022;71:111–8. 10.1136/gutjnl-2021-325096 34210775

[26] Lai S , Liao K , Lai H , et al . Use of proton pump inhibitors correlates with increased risk of colorectal cancer in T Aiwan. Asia-Pac J Clncl Oncology 2013;9:192–3. 10.1111/ajco.12054 23298363

[27] Hwang IC , Chang J , Park SM . Emerging hazard effects of proton pump inhibitor on the risk of colorectal cancer in low-risk populations: a Korean nationwide prospective cohort study. PLoS One 2017;12:e0189114. 10.1371/journal.pone.0189114 29216279 PMC5720708

[28] Thrumurthy SG , Thrumurthy SSD , Gilbert CE , et al . n.d. Colorectal adenocarcinoma: risks, prevention and diagnosis. BMJ:i3590. 10.1136/bmj.i3590 27418368

[29] Baran B , Mert Ozupek N , Yerli Tetik N , et al . Difference between left-sided and right-sided colorectal cancer: a focused review of literature. Gastroenterology Res 2018;11:264–73. 10.14740/gr1062w 30116425 PMC6089587

[30] Strand DS , Kim D , Peura DA . 25 years of proton pump inhibitors: a comprehensive review. Gut Liver 2017;11:27–37. 10.5009/gnl15502 27840364 PMC5221858

[31] Socialstyrelsen . The National patient register- socialstyrelsen. 2019. Available: https://www.socialstyrelsen.se/en/statistics-and-data/registers/register-information/the-national-patient-register/

[32] Swedish cancer Registry quality. Available: https://www.socialstyrelsen.se/en/statistics-and-data/registers/register-information/swedish-cancer-register/

[33] Ludvigsson JF , Almqvist C , Bonamy A-KE , et al . Registers of the Swedish total population and their use in medical research. Eur J Epidemiol 2016;31:125–36. 10.1007/s10654-016-0117-y 26769609

[34] Brooke HL , Talbäck M , Hörnblad J , et al . The Swedish cause of death register. Eur J Epidemiol 2017;32:765–73. 10.1007/s10654-017-0316-1 28983736 PMC5662659

[35] Wallerstedt SM , Wettermark B , Hoffmann M . The first decade with the Swedish prescribed drug register – A systematic review of the output in the scientific literature. Basic Clin Pharma Tox 2016;119:464–9. 10.1111/bcpt.12613 27112967

[36] von Elm E , Altman DG , Egger M , et al . The strengthening the reporting of observational studies in epidemiology (STROBE) statement: guidelines for reporting observational studies. The Lancet 2007;370:1453–7. 10.1016/S0140-6736(07)61602-X 18064739

[37] FASS general - home. 2021. Available: https://www.fass.se/LIF/startpage

[38] Barlow L , Westergren K , Holmberg L , et al . The completeness of the Swedish cancer register – a sample survey for year 1998. Acta Oncologica 2009;48:27–33. 10.1080/02841860802247664 18767000

[39] Vogel JD , Eskicioglu C , Weiser MR , et al . The American society of colon and rectal Surgeons clinical practice guidelines for the treatment of colon cancer. Dis Colon Rectum 2017;60:999–1017. 10.1097/DCR.0000000000000926 28891842

[40] Brusselaers N , Lagergren J . Maintenance use of non-Steroidal anti-inflammatory drugs and risk of gastrointestinal cancer in a nationwide population-based cohort study in Sweden. BMJ Open 2018;8:e021869. 10.1136/bmjopen-2018-021869 PMC6042574 29982219

[41] Machin D , Breslow NE , Day NE . Statistical methods in cancer research. In: The Design and Analysis of Cohort Studies. Biometrics [Internet]. 1990. Available: https://www.jstor.org/stable/2532481?origin=crossref

[42] Statistics database - national board of health and welfare. 2024. Available: https://www.socialstyrelsen.se/statistik-och-data/statistik/statistikdatabasen/

[43] Bender R . Introduction to the use of regression models in epidemiology. Methods Mol Biol 2009;471:179–95. 10.1007/978-1-59745-416-2_9 19109780

[44] Simon K . Colorectal cancer development and advances in screening. Clin Interv Aging 2016;11:967–76. 10.2147/CIA.S109285 27486317 PMC4958365

[45] Chan AT , Giovannucci EL , Meyerhardt JA , et al . Long-term use of aspirin and nonsteroidal anti-inflammatory drugs and risk of colorectal cancer. JAMA 2005;294:914–23. 10.1001/jama.294.8.914 16118381 PMC1550973

[46] Zhong L , Shanahan ER , Raj A , et al . Dyspepsia and the Microbiome: time to focus on the small intestine. Gut 2017;66:1168–9. 10.1136/gutjnl-2016-312574 27489239

[47] Minalyan A , Gabrielyan L , Scott D , et al . The gastric and intestinal Microbiome: role of proton pump inhibitors. Curr Gastroenterol Rep 2017;19:42. 10.1007/s11894-017-0577-6 28733944 PMC5621514

[48] Macke L , Schulz C , Koletzko L , et al . Systematic review: the effects of proton pump inhibitors on the Microbiome of the digestive tract-evidence from next-generation sequencing studies. Aliment Pharmacol Ther 2020;51:505–26. 10.1111/apt.15604 31990420

[49] Robertson DJ , Sandler RS , Ahnen DJ , et al . Gastrin, Helicobacter Pylori, and colorectal adenomas. Clinical Gastroenterology and Hepatology 2009;7:163–7. 10.1016/j.cgh.2008.09.006 18929688

[50] Effects of three H2-receptor antagonists (cimetidine, Famotidine, ranitidine) on serum gastrin Level- PubMed. 2024. Available: https://pubmed.ncbi.nlm.nih.gov/12503773/ 12503773

[51] Baldwin GS , Shulkes A . Gastrin as an autocrine growth factor in colorectal carcinoma: implications for therapy. WJG 1998;4:461. 10.3748/wjg.v4.i6.461 11819345 PMC4723428

[52] Babic A , Zhang X , Morales-Oyarvide V , et al . Acid-suppressive medications and risk of colorectal cancer: results from three large prospective cohort studies. Br J Cancer 2020;123:844–51. 10.1038/s41416-020-0939-y 32541871 PMC7462971

[53] Population statistics. 2023. Available: https://www.scb.se/en/finding-statistics/statistics-by-subject-area/population/population-composition/population-statistics/

[54] Brusselaers N , Wahlin K , Engstrand L , et al . Maintenance therapy with proton pump inhibitors and risk of gastric cancer: a nationwide population-based cohort study in Sweden. BMJ Open 2017;7:e017739. 10.1136/bmjopen-2017-017739 PMC5665226 29084798

[55] Calvo LLJ , García Cámara P , Llorente Barrio M , et al . Successful deprescribing of proton pump inhibitors with a patient-centered process: the DESPIBP project. Eur J Clin Pharmacol 2021;77:1927–33. 10.1007/s00228-021-03186-x 34269841

[56] Muheim L , Signorell A , Markun S , et al . Potentially inappropriate proton-pump inhibitor prescription in the general population: a claims-based retrospective time trend analysis. Therap Adv Gastroenterol 2021;14. 10.1177/1756284821998928 PMC8053831 33948109

[57] Brenner DR , Ruan Y , Shaw E , et al . Increasing colorectal cancer incidence trends among younger adults in Canada. Prev Med 2017;105:345–9. 10.1016/j.ypmed.2017.10.007 28987338

[58] Vuik FE , Nieuwenburg SA , Bardou M , et al . Increasing incidence of colorectal cancer in young adults in Europe over the last 25 years. Gut 2019;68:1820–6. 10.1136/gutjnl-2018-317592 31097539 PMC6839794

[59] Potter JD . n.d. Rising rates of colorectal cancer in younger adults. BMJ. 10.1136/bmj.l4280 31235545

[60] Meguid RA , Slidell MB , Wolfgang CL , et al . Is there a difference in survival between Right- versus left-sided colon cancers? Ann Surg Oncol 2008;15:2388–94. 10.1245/s10434-008-0015-y 18622647 PMC3072702

[61] Mik M , Berut M , Dziki L , et al . Right-and left-sided colon cancer-clinical and pathological differences of the disease entity in one organ. Arch Med Sci 2017;13:157–62. 10.5114/aoms.2016.58596 28144267 PMC5206358

