# Supplementary material for: Maintenance proton pump inhibitor use and risk of colorectal cancer: a Swedish retrospective cohort study
Source: BMJ Open. 2024 Jul 2;14(7):e079591. doi: 10.1136/bmjopen-2023-079591 (PMC11227764; doi:10.1136/bmjopen-2023-079591)

Supplementary Materials:

Table S1. International Classification of Diseases code for oncology and Anatomical Therapeutic Chemical codes used in this study

Table S2. Information of colorectal adenocarcinoma in the Swedish total population between 2005 and 2012

Table S3. Risk of colorectal adenocarcinomas in maintenance proton pump inhibitor (PPI) users followed up from enrolment, expressed as standardised incidence ratios (SIRs) and 95% confidence intervals (CIs) by age, sex, calendar period, tumour subsites and stages

Figure S1. Flow chart illustrating the enrolment of the study population of (**a**) maintenance proton pump inhibitor (PPI) users (**b**) maintenance histamine-2 receptor antagonist (H_2RA) users

Figure S2. Charts showing the crude incidence of Swedish total population.

Table S1. International Classification of Diseases code for oncology and Anatomical Therapeutic Chemical codes used in this study

Diseases	International Classification of Diseases code for oncology, 10th edition	Dispensed drugs	Anatomical Therapeutic Chemical code
Colorectal cancer	C18 -C20	Proton pump inhibitor	A02BC
Adenocarcinoma	096	Non-steroidal anti-inflammatory drug	M01A
Right-sided colorectal cancer	C18.0 -C18.4	Low-dose aspirin	B01AC06
Left-sided colorectal cancer	C18.5 - C18.7, C19, C20	Histamine-2 receptor antagonist	A02BA
Overlapping or unspecified sites of colorectal cancer	C18.8 C18.9		

Table S2. Information of colorectal adenocarcinoma in the Swedish total population between 2005 and 2012

Characteristics	Colorectal adenocarcinoma
Total	34,765
Age at entry the cohort, years	
18-39 years	532
40-49 years	1,324
50-59 years	3,974
60-69 years	9,298
≥70 years	19,637
Sex	
Men	18,601
Women	16,164
Calendar period	
2005-2006	7,998
2007-2009	13,044
2010-2012	13,723
Colorectal adenocarcinoma	
Right-sided	11,743
Left-sided	22,229
Overlapping or unspecified	793

Table S3. Risk of colorectal adenocarcinomas in maintenance proton pump inhibitor (PPI) users followed from enrolment, expressed as standardised incidence ratios (SIRs) and 95% confidence intervals (CIs) by age, sex, calendar period, tumour subsites and stages

	Maintenance PPI users with maximum follow-up time	
	Number	SIRs (95% CI)
Total colorectal adenocarcinomas	6,887	1.44 (1.40-1.47)
Sex		
Men	3,341	1.48 (1.43-1.53)
Women	3,546	1.40 (1.35-1.44)
Age at starting the maintenance PPI use, years		
18-39	67	5.53 (4.03-7.39)
40-49	218	2.75 (2.35-3.19)
50-59	775	1.78 (1.64-1.94)
60-69	1,870	1.60 (1.52-1.68)
≥70	3,957	1.32 (1.28-1.36)
Calendar year period		
2005-2006	4,126	1.76 (1.65-1.88)
2007-2009	1,982	1.55 (1.49-1.60)
2010-2012	779	1.28 (1.24-1.33)
Subsites of colorectal adenocarcinoma		
Right-sided	3,043	1.74 (1.68-1.81)
Left-sided	3,710	1.25 (1.21-1.29)
Stages of colorectal adenocarcinoma		
Stage 0-I	966	1.49 (1.40-1.59)
Stage II	1,657	1.41 (1.34-1.48)
Stage III	1,561	1.40 (1.33-1.47)
Stage IV	1,172	1.27 (1.19-1.34)
Missing	1,531	1.62 (1.54-1.70)
Follow-up time, years (median)	6.1	-

Figure S1. Flow chart illustrating the enrolment of the study population of (**a**) maintenance proton pump inhibitor (PPI) users (**b**) maintenance histamine-2 receptor antagonist (H_2RA) users

(**a**) maintenance PPI users

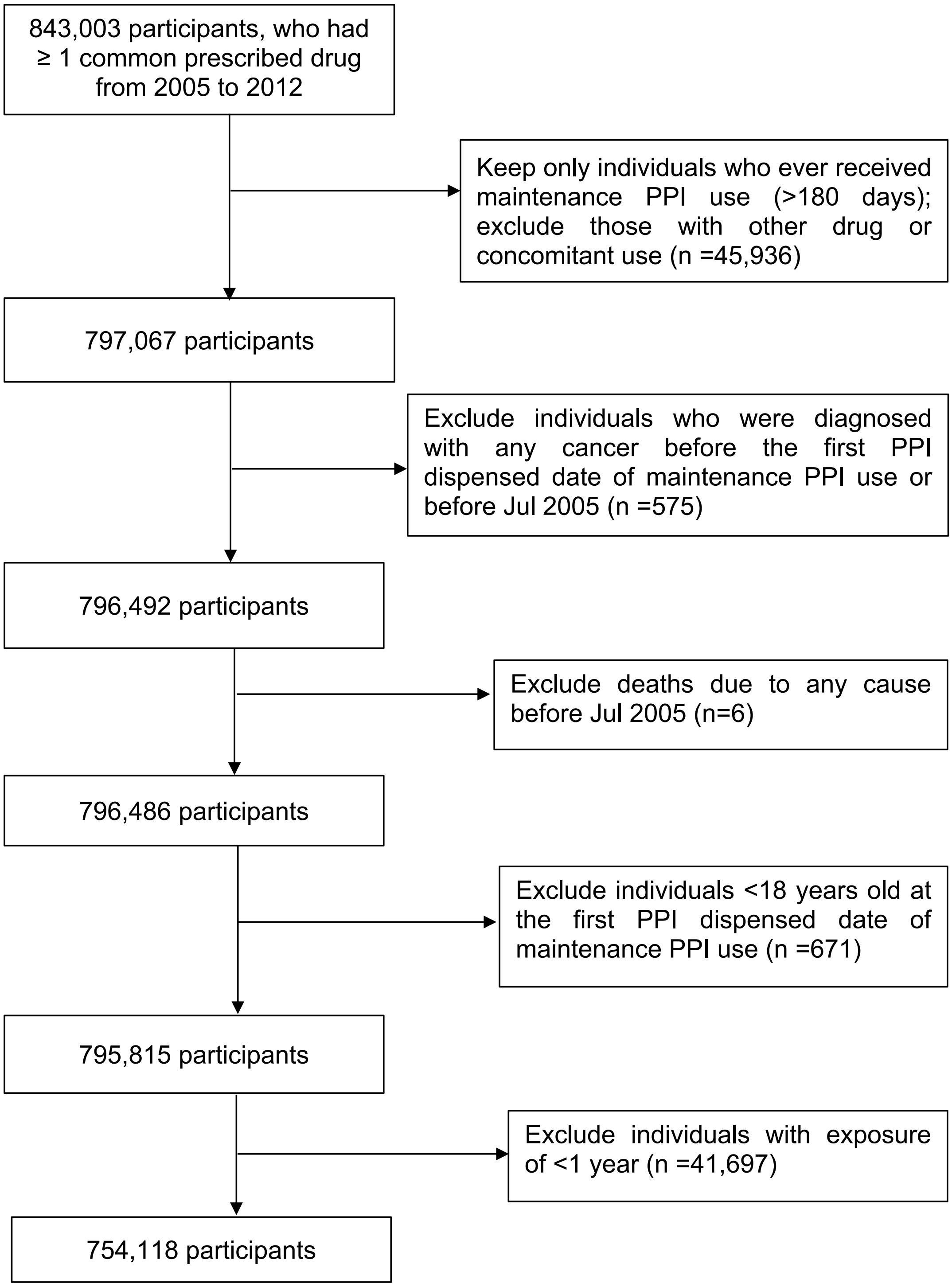

(**b**) maintenance H_2RA users

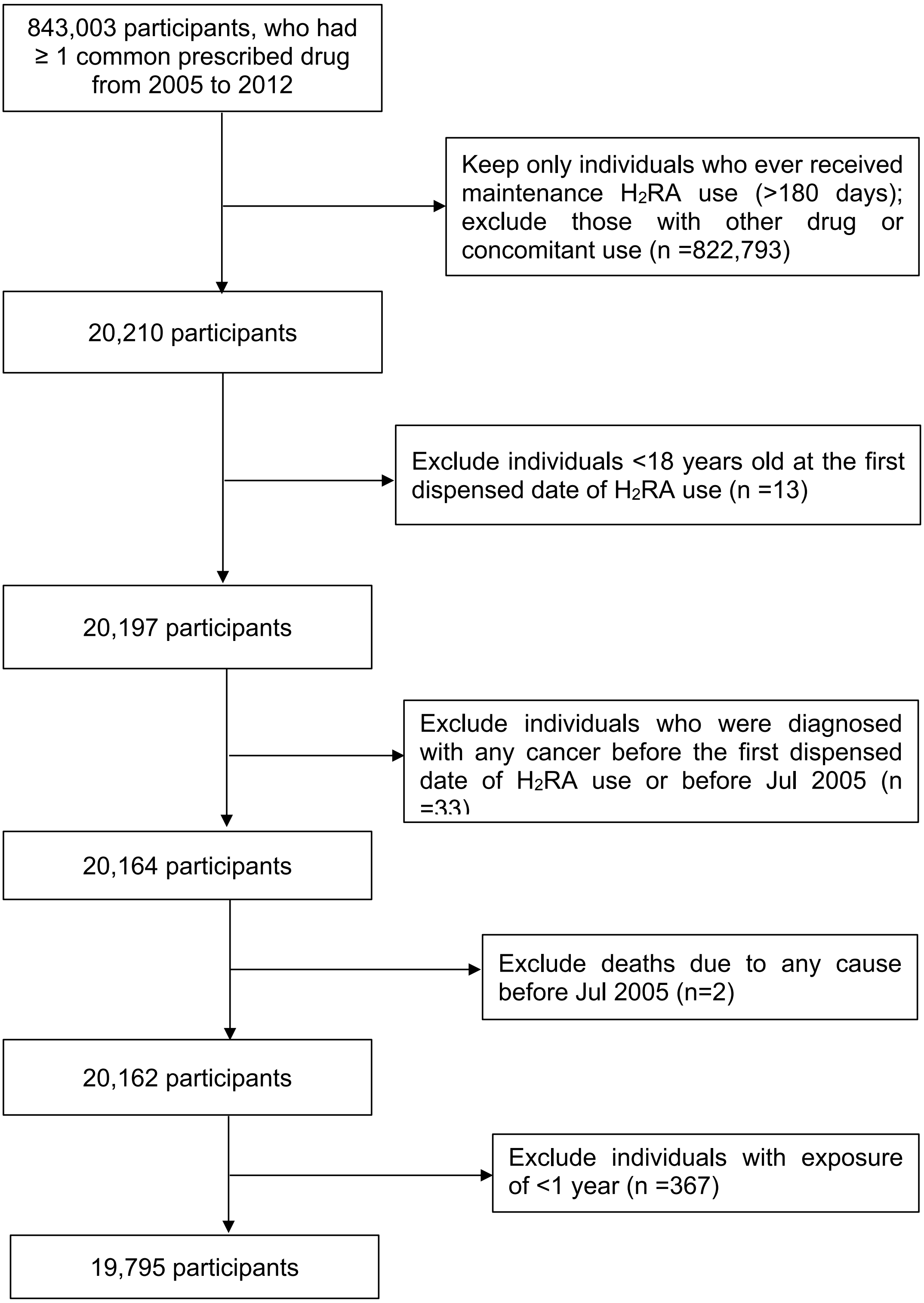

Figure S2. Charts showing the crude incidence of Swedish total population. Data were retrieved from the National Board of Health and Welfare, Statistical Database.

(**a**) Crude incidence of new colorectal adenocarcinoma cases (over 20 years old) in Sweden (2012), by sex

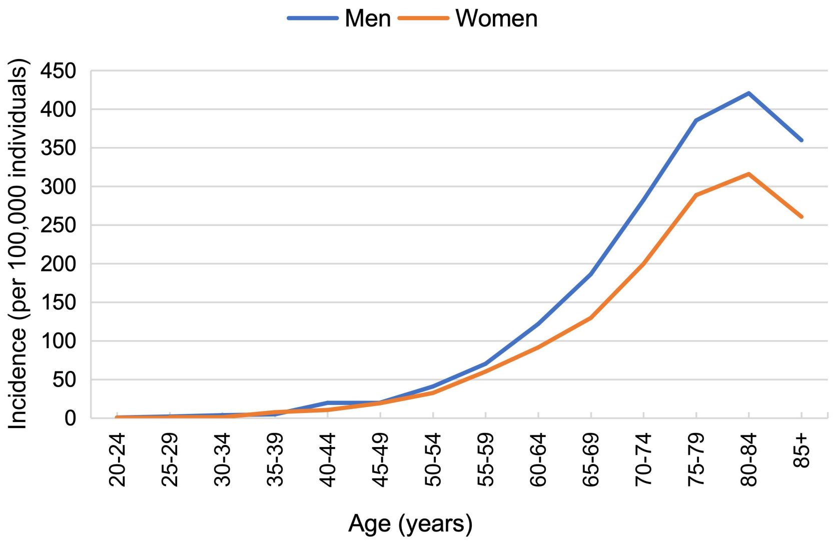

(**b**) Crude incidence of new colorectal adenocarcinoma cases in Sweden (2006-2012), by sex

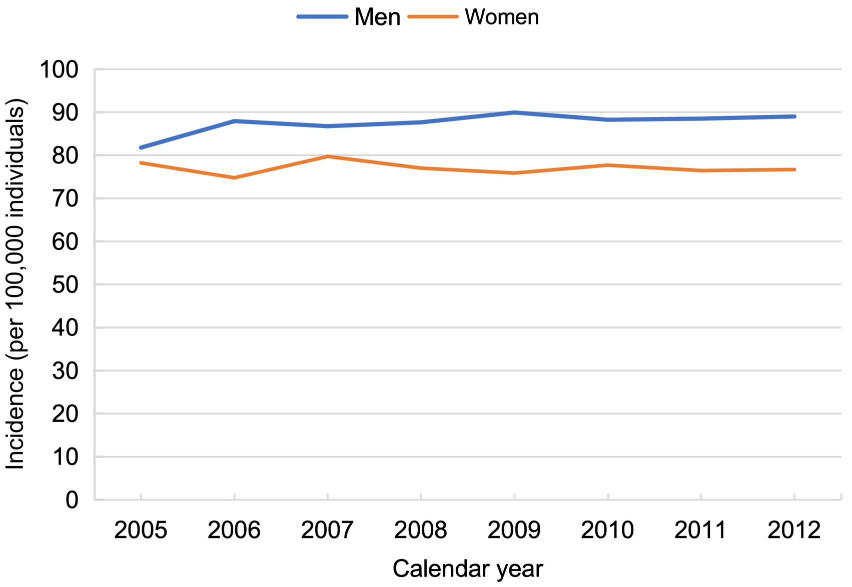

Supplement: Supplementary data [file bmjopen-2023-079591supp001.pdf]
