# Supplementary material for: Maintenance proton pump inhibitor use and risk of colorectal cancer: a Swedish retrospective cohort study
Source: BMJ Open. 2024 Jul 2;14(7):e079591. doi: 10.1136/bmjopen-2023-079591 (PMC11227764; doi:10.1136/bmjopen-2023-079591)
Supplement: Author's manuscript [file bmjopen-2023-079591.draft_revisions.pdf]

BMJ Open is committed to open peer review. As part of this commitment we make the peer review history of every article we publish publicly available.

When an article is published we post the peer reviewers' comments and the authors' responses online. We also post the versions of the paper that were used during peer review. These are the versions that the peer review comments apply to.

The versions of the paper that follow are the versions that were submitted during the peer review process. They are not the versions of record or the final published versions. They should not be cited or distributed as the published version of this manuscript.

BMJ Open is an open access journal and the full, final, typeset and author-corrected version of record of the manuscript is available on our site with no access controls, subscription charges or pay-per-view fees (http://bmjopen.bmj.com).

If you have any questions on BMJ Open's open peer review process please email info.bmjopen@bmj.com

BMJ Open

Maintenance proton pump inhibitor use and risk of colorectal cancer: A population-based Swedish cohort study

Journal:	*BMJ Open*
Manuscript ID	bmjopen-2023-079591
Article Type:	Original research
Date Submitted by the Author:	05-Sep-2023
Complete List of Authors:	Liu, Qing; Karolinska Institutet, Department of Microbiology, Tumour and Cell Biology<br>WANG, Xinchen; Karolinska Institutet, Department of Clinical Neuroscience<br>Engstrand, Lars; Karolinska Institutet, Microbiology, Tumour and Cell Biology<br>Sadr-Azodi, Omid; Karolinska Institutet<br>Fall, Katja; Karolinska Institutet, Dept of Medical Epidemiology and Biostatistic<br>Brusselaers, Nele; Karolinska Institutet, Molecular medicine and Surgery
Keywords:	CLINICAL PHARMACOLOGY, EPIDEMIOLOGIC STUDIES, GASTROENTEROLOGY, Gastrointestinal tumours < GASTROENTEROLOGY, REGISTRIES, Risk Factors

SCHOLARONE™
Manuscripts

I, the Submitting Author has the right to grant and does grant on behalf of all authors of the Work (as defined in the below author licence), an exclusive licence and/or a non-exclusive licence for contributions from authors who are: i) UK Crown employees; ii) where BMJ has agreed a CC-BY licence shall apply, and/or iii) in accordance with the terms applicable for US Federal Government officers or employees acting as part of their official duties; on a worldwide, perpetual, irrevocable, royalty-free basis to BMJ Publishing Group Ltd ("BMJ") its licensees and where the relevant Journal is co-owned by BMJ to the co-owners of the Journal, to publish the Work in this journal and any other BMJ products and to exploit all rights, as set out in our licence.

The Submitting Author accepts and understands that any supply made under these terms is made by BMJ to the Submitting Author unless you are acting as an employee on behalf of your employer or a postgraduate student of an affiliated institution which is paying any applicable article publishing charge ("APC") for Open Access articles. Where the Submitting Author wishes to make the Work available on an Open Access basis (and intends to pay the relevant APC), the terms of reuse of such Open Access shall be governed by a Creative Commons licence – details of these licences and which Creative Commons licence will apply to this Work are set out in our licence referred to above.

Other than as permitted in any relevant BMJ Author's Self Archiving Policies, I confirm this Work has not been accepted for publication elsewhere, is not being considered for publication elsewhere and does not duplicate material already published. I confirm all authors consent to publication of this Work and authorise the granting of this licence.

For peer review only

Title: Maintenance proton pump inhibitor use and risk of colorectal cancer: A population-based Swedish cohort study

Authors:

Qing LIU [1*], Xinchen WANG [2], Lars ENGSTRAND [1], Omid SADR-AZODI [3,4], Katja FALL[5,6], Nele BRUSSELAERS [1,7,8*]

Affiliations:

1 Centre for Translational Microbiome Research, Department of Microbiology, Tumour and Cell Biology, Karolinska Institutet, Stockholm, Sweden

2 Centre for Psychiatry Research, Department of Clinical Neuroscience, Karolinska Institutet, Stockholm, Sweden

3 Department of Clinical Sciences, Intervention and Technology, Karolinska Institutet, Stockholm, Sweden

4 Department of Surgery, Capio Saint Göran Hospital, Stockholm, Sweden

5 Clinical Epidemiology and Biostatistics School of Medical Sciences, Örebro University, Örebro, Sweden

6 Institute of Environmental Medicine, Karolinska Institutet, Stockholm, Sweden

7 Department of Head and Skin, Ghent University, Ghent, Belgium

8 Global Health Institute, Antwerp University, Antwerp, Belgium

Corresponding author: Qing Liu, qing.liu2017@outlook.com. Centre for Translational Microbiome Research, Department of Microbiology, Tumour and Cell biology, Karolinska Institutet. Biomedicum, kvarter 8a. Solnavägen 9, SE-171 65 Stockholm. Telephone: +46 (0) 8-524 845 97.

For peer review only

Abstract

Objectives: We aimed to evaluate the risk of colorectal adenocarcinoma (CRA) associated with long-term use of proton pump inhibitors (PPIs) in a large nationwide cohort.

Design: Retrospective cohort study.

Setting: This research was conducted at the national level, encompassing the entire population of Sweden.

Participants: This study utilized Swedish national registries to identify all adults who had ≥180 days of cumulative PPI use between July 2005 and December 2012, excluding participants who were followed up for less than one year. A total of 754,118 maintenance PPI users were included, with a maximum follow-up of 7.5 years.

Interventions: Maintenance PPI use (cumulative ≥180 days), with a comparator of maintenance histamine-2 receptor antagonist (H_2RA) use.

Primary and Secondary Outcome Measures: The primary outcome measure was the risk of CRA, presented as standardised incidence ratios (SIRs) with 95% confidence intervals (CIs). Subgroup analyses were performed to explore the impact of indications, tumour locations, tumour stages, and the duration of follow-up. A multivariable Poisson regression model was fitted to estimate the incidence rate ratios (IRRs) and 95% CIs of PPI versus H_2RA use.

Results: Maintenance PPI users exhibited a slightly elevated risk of CRA compared to the general population (SIR 1.10, 95% CI= 1.06-1.13) for both men and women. Individuals aged 18-39 (SIR 2.79, 95% CI=1.62-4.47) and 40-49 (SIR 2.02, 95% CI=1.65-2.45) had significantly higher risks than the general population. Right-sided CRA showed a higher risk compared to the general population (SIR 1.26, 95% CI=1.20-1.32). There was no significant difference in the risk of CRA between maintenance PPI users and maintenance H2RA users (IRR 1.05, 95% CI=0.87-1.27, P<0.05).

Conclusions: Maintenance PPI use may be associated with an increased risk of CRA, but a prolonged observation time is needed.

Keywords: Cancer risk, Colorectal cancer, Proton pump inhibitors, Carcinogenesis, Histamine-2 receptor antagonist

Strengths and limitations of this study

Strengths

- High number of enrolled individuals and person-years
- High external validity
- Not affected by migrations and drop-offs
- Reduced residual confounding by the active comparator

Limitations

- Lack of additional data on some lifestyle risk factors
- Potential confounding introduced by ever users who received maintenance PPI use long before the study period
- Longer follow-up (i.e., more than 10 years) may be needed

For peer review only

Introduction

Proton pump inhibitors (PPIs) are one of the most commonly prescribed drugs globally and are widely used for the treatment and prevention of various gastric acid-related disorders(1,2). The use of both prescription and over-the-counter PPIs has increased in many countries, with an estimated 10-30% of adults regularly using PPIs(3,4). Although PPIs have been deemed safe for short-term use and are recommended for a broad spectrum of indications by current guidelines and expert reviews(5–7), only a few indications justify long-term use exceeding 4 weeks. Despite this, PPIs are often overprescribed(7,8), which highlights the need for further investigation into the potential long-term side effects and public health implications of their widespread use.

Long-term PPI use has been associated with an increased risk of gastrointestinal cancers, including gastric, oesophageal and pancreatic cancers(9–11). Previous studies have demonstrated that PPI use significantly impacts the gut microbiome and contributes to dysbiosis(12–14), which has been linked to oncogenesis in the gut epithelium(15,16). Enteric infections and the trophic effects of PPI-induced hypergastrinaemia may also serve as potential cancerogenic factors(17). While some cohort studies have reported elevated relative risks of colorectal cancer (CRC) following PPI use(18,19), others have found no association between CRC and PPI use12–15. However, potential methodological limitations in some of these studies have impacted the results. For example, some case-control studies introduced selection bias in exposure effect estimation by enrolling prevalent users rather than new users(22,23,25,27), and latency bias and time-window bias were present in some previous cohort studies due to the selection of narrow observation time ranges (19,22,28).

To clarify the relationship between PPI use and CRC, we conducted a large cohort study using high-coverage national registries to control for immortal time bias and time-window bias. We aimed to assess the association between maintenance PPI use and the risk of colorectal adenocarcinoma (CRA), which accounts for >95% of all CRC cases(29). We focused on right-sided and left-sided CRAs due to their chronological exposure to the drug-effects based on the anatomical locations(29,30). Additionally, we used the less popular histamine-2 receptor antagonists (H_2RAs) as active comparators in the supplementary analysis to minimise confounding-by-indication, as they share similar indications with PPIs and were used for treating gastric acid-related disorders before the introduction of PPIs as more potent acid suppressants(31).

Methods

Study design

This was a register-based cohort study that enrolled all Swedish adults (aged 18 years old) who were on maintenance PPI therapy between July 2005 and December 2012.

The study population was compared to the entire Swedish population stratified by the same age, sex, and calendar period. Participants were followed from one year after the first dispensed prescription date until the first diagnosis of CRA, death or the end of the study period (December 31, 2012). Data were obtained from the Swedish Cancer Registry, the National Patient Registry, the Cause of Death Registry, the Prescribed Drug Registry, and the Registry of the Total Population(32–36). The study protocol was established a priori, and the study was conducted in accordance with the Strengthening the Reporting of Observational Studies in Epidemiology (STROBE) statement(37). No individual informed consent was required due to the registry-based nature of the data. Ethical approval was obtained from the Regional Ethical Review Board in Stockholm (2014/1291-31/4).

Patient and public involvement

Due to its data-driven nature, this retrospective study had limited patient and public involvement. Patients were not involved in the study's design, data analysis, method selection or dissemination planning. Research questions and outcome measures were derived from existing literature and clinical relevance without direct input from them. Recruitment and follow-up relied on existing records from national registries.

Exposure

PPI usage data were obtained from the Prescribed Drug Registry, labelled by the (ATC)/defined daily dose (DDD) system. This system annotated the DDD per prescribed package with ATC codes. PPIs are also available over-the-counter in Sweden but only in small quantities and at exorbitant prices in comparison to prescribed PPIs(38). Therefore, most PPI consumption of long-term users is likely recorded in this registry. Maintenance PPI use (ATC code A02BC) was defined as cumulative use for at least 180 days prior to the onset of any cancer, death, or the end of the study period. Maintenance H_2RA (ATC code A02BA) use was used as an active comparator.

Outcome

The primary outcome was CRA, defined using the International Classification of Diseases (ICD, 10th edition) codes C18-C20 from the Swedish Cancer Registry(39), and the histopathology code 096. The tumours were further classified based on their anatomical location as right-sided, left-sided, or overlapping/unspecified sites (ICD codes in Supplementary Table S1). The tumour-node-metastasis (TNM) classification of the American Joint Committee on Cancer 8th edition Staging of Colorectal Cancer was used to define tumour stage and grouped as follows: stage 0-I (Tis, N0, M0 for stage 0; T1-2, N0, M0 for stage I), II (T3-4, N0, M0), III (T1-4, N1-2, M0), and IV (any T, any N, M1)(40).

Exclusions

For peer review only - http://bmjopen.bmj.com/site/about/guidelines.xhtml

Individuals with any documented cancer diagnosis prior to the study period or prior to the first dispensing date of PPIs were excluded. Individuals who switched between maintenance use of PPIs and H_2RAs (defined as ≥180 days of accumulated use of either drug) or had an exposure period of less than one year were also excluded. (Supplementary Figure S1)

Covariates

Age at treatment initiation was categorised into 18-39, 40-49, 50-59, 60-69 and ≥ 70 years old, and calendar periods were categorised into 2005-2006, 2007-2009, and 2010-2012. The duration of time since drug initiation was categorised as 1-2, 2-4, 4-6, and ≥ 6 years. No missing values for these variables were found for any of the study participants.

The most common recorded indications of maintenance PPI use were extracted from the National Patient Registry, including *Helicobacter pylori* infection or eradication, gastroesophageal reflux, Barrett's oesophagus, peptic ulcer, Zollinger Ellison's syndrome, gastro-duodenitis, dyspepsia, maintenance use of low-dose aspirin, and maintenance use of NSAIDs(9,11,41) (ICD codes in Supplementary Table S1). We performed subgroup analyses on individuals with and without the listed indications, as well as on each indication separately when the statistical power was sufficient.

Statistical analyses

We calculated standardised incidence ratios (SIRs) with 95% confidence intervals (CIs) standardised by age, sex, and calendar period to assess the association between maintenance PPI use and CRA(42). In our main analysis, we compared the overall risk of CRA in the study population to the general population of Sweden, stratifying the main outcomes by tumour stage (0-I, II, III, IV, and missing values), tumour location (right-sided, left-sided), indications, and follow-up time since drug initiation (1-2, 2-4, 4-6, and ≥ 6 years). We extensively investigated the risk of different tumour locations by age, sex, and indications for maintenance PPI use. Additionally, we assessed the risk of CRA in maintenance PPI users with a maximal follow-up time from the first dispensed prescription date, considering age, sex, calendar period, tumour stage, and tumour location.

Furthermore, we conducted two supplementary analyses. First, we estimated SIRs for maintenance H_2RA users and conducted a duration analysis (1-2, 2-4, 4-6, and ≥ 6 years). Second, we used a multivariable Poisson regression model to estimate incidence rate ratios (IRRs) with 95% CIs to compare CRA risk between maintenance PPI users and H_2RA users, adjusting for age, sex and common therapy indications(43). Propensity score adjustment between maintenance PPI users and H_2RA users was performed in the model to better control for confounders. We considered *p* values less

than 0.05 statistically significant for all the analyses. Stata MP 14.2 was used for all calculations.

Results

1. Characteristics of the PPI cohort

This study included 754,118 adult maintenance PPI users with no prior history of cancer, with a median follow-up of 5.3 years (totalling 4,177,396 person-years) and a maximum follow-up of 7.5 years. Women (0.52%) had a lower cumulative incidence of CRA than men (0.62%). Maintenance PPI use was most common in individuals aged 60-69 and ≥70 years old (Tables 1 & 2). A total of 4,432 cases of CRA were identified, with the majority being left-sided (N=2,499, 56.4%), stage II (N=1,102, 24.9%) and III (N=1,017, 22.9%).

Table 1. Characteristics of all maintenance proton pump inhibitor (PPI) users in Sweden between 2005 and 2012

Characteristics of the study population	Maintenance PPI users	
	Number	%
Total	754,118	100.0
Age at starting the maintenance PPI use, years		
18-39 years	84,510	11.2
40-49 years	99,897	13.3
50-59 years	149,167	19.8
60-69 years	167,456	22.2
≥70 years	253,088	33.6
Sex		
Men	310,357	41.2
Women	443,761	58.9
Calendar period		
2005-2006	430,527	57.1
2007-2009	221,369	29.4
2010-2012	102,222	13.6
Colorectal adenocarcinoma	4,432	0.6
Right-sided	1,884	0.3
Left-sided	2,499	0.3
Overlapping or unspecified	49	0.0
Tumour stages of colorectal adenocarcinoma		
Stage 0-I	674	0.1
Stage II	1,102	0.1
Stage III	1,017	0.1
Stage IV	748	0.1

For peer review only

Missing	891	0.1
Indications for maintenance PPI use		
Helicobacter pylori infection or eradication	55,311	7.3
Gastroesophageal reflux	193,371	25.6
Barrett's oesophagus	5,717	0.8
Peptic ulcer	74,946	10.0
Zollinger Ellison's syndrome	28	0.0
Gastro-duodenitis	100,191	13.2
Dyspepsia	41,916	5.6
Aspirin maintenance use	262,242	34.8
Non-steroidal anti-inflammatory drugs maintenance use	231,792	30.7
Without any of these recorded indications	186,529	24.7
Maintenance PPI use based on daily drug usage		
Interquartile range	1810	-
Person-years	3,426,925	-
Follow-up time, years (median)	5.3	-

Table 2. Risk of colorectal adenocarcinomas in maintenance proton pump inhibitor (PPI) users, expressed as standardised incidence ratios (SIRs) and 95% confidence intervals (CIs) by age, sex, calendar period, tumour subsites and stages

	Maintenance PPI users	
	Number	SIRs (95% CI)
Total colorectal adenocarcinomas	4,432	1.10 (1.06-1.13)
Sex		
Men	2,113	1.13 (1.08-1.18)
Women	2,319	1.10 (1.06-1.15)
Age at starting the maintenance PPI use, years		
18-39	38	2.79 (1.62-4.47)
40-49	145	2.02 (1.65-2.45)
50-59	531	1.27 (1.13-1.41)
60-69	1,277	1.26 (1.19-1.34)
≥70	2,441	1.04 (1.00-1.07)
Calendar year period		
2005-2006	3,215	1.29 (1.09-1.51)
2007-2009	1,049	1.17 (1.12-1.22)
2010-2012	168	1.07 (1.02-1.11)

For peer review only

Subsites of colorectal adenocarcinoma		
Right-sided	1,884	1.26 (1.20-1.32)
Left-sided	2,499	0.99 (0.96-1.03)
Stages of colorectal adenocarcinoma		
Stage 0-I	674	1.22 (1.13-1.32)
Stage II	1,102	1.10 (1.03-1.16)
Stage III	1,017	1.07 (1.00-1.13)
Stage IV	748	0.95 (0.88-1.02)
Missing	891	1.19 (1.11-1.27)

2. Risk of colorectal adenocarcinoma

Compared to the general population, the overall risk of CRA was increased in maintenance PPI users (SIR 1.10, 95% CI 1.06-1.13). This risk was particularly affected by age, with the highest estimates present in those aged 18-39 years (SIR 2.79, 95% CI 1.62-4.47) and 40-49 years (SIR 2.02, 95% CI 1.65-2.45), while no convincing association was found in individuals ≥70 years old (SIR 1.04, 95% CI 1.00-1.07). In maintenance PPI users, the diagnosed CRA was particularly associated with an increased risk of early stage (Stage 0-I, SIR 1.12, 95% CI 1.04-1.21) (Table 2). In terms of tumour location, maintenance PPI users had a higher risk of right-sided CRA than the general population, regardless of age and sex (Tables 2&3). Meanwhile, no association between PPI use and CRA risk was found in left-sided CRC (Tables 2&3).

Table 3. Sex and age disparities in the risk of right-sided and left-sided colorectal adenocarcinomas in maintenance proton pump inhibitor (PPI) users expressed as standardised incidence ratios (SIRs) and 95% confidence intervals (CIs)

Cancer in categories	Right-sided colorectal adenocarcinoma			Left-sided colorectal adenocarcinoma		
	Number	SIR (95 % CI)	Forest plot	Number	SIR (95 % CI)	Forest plot
Sex						
Men	736	1.25 (1.16-1.34)		1,359	1.04 (0.98-1.10)	
Women	1,148	1.27 (1.19-1.34)		1,14	0.94 (0.89-1.00)	
Age at starting the maintenance PPI, years						
18-39	13	2.78 (1.02-6.06)		24	2.81 (1.40-5.03)	
40-49	37	1.98 (1.31-2.89)		103	1.95 (1.53-2.46)	
50-59	167	1.42 (1.14-1.75)		359	1.20 (1.05-1.37)	
60-69	528	1.58 (1.43-1.75)		732	1.12 (1.03-1.21)	
≥70	1,139	1.17 (1.11-1.24)		1,281	0.90 (0.86-0.95)	

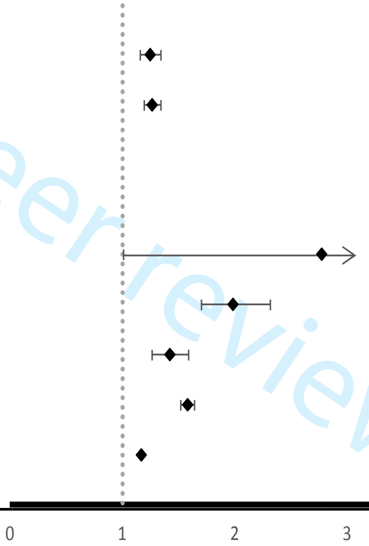

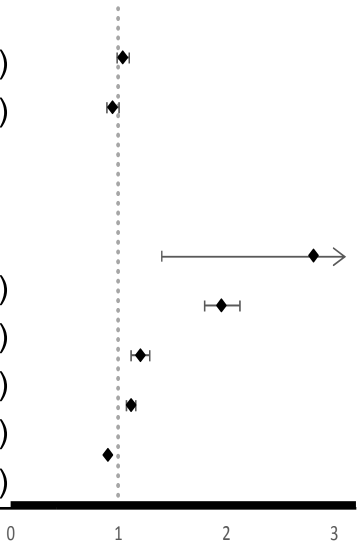

For peer review only

3. Common indications for maintenance PPI use

Indications for maintenance PPI use did not show the same trend for the drug-cancer-risk relationship in the exposed population. PPI use was not related to an increased risk of CRA among patients with Barrett's oesophagus. A marginally increased risk of CRA among PPI users was observed in participants with the indications of *Helicobacter pylori* infection or eradication (SIR 1.29, 95% CI 1.15-1.44), dyspepsia (SIR 1.29, 95% CI 1.15-1.44), gastro-duodenitis (SIR 1.28, 95% CI 1.19-1.38), gastroesophageal reflux (SIR 1.26, 95% CI 1.19-1.33), and peptic ulcer (SIR 1.16, 95% CI 1.07-1.26). Among individuals with the combined use of non-steroidal anti-inflammatory drugs or low-dose aspirin, maintenance PPI use was not significantly associated with the risk of CRA (Table 4**a**). Compared with the general population, most of the indications were related to an increased risk of right-sided CRAs in maintenance PPI users (Table 4**b**).

For peer review only

Table 4. Risk of (**a**) colorectal adenocarcinomas (**b**) right-sided and left-sided colorectal adenocarcinomas according to indications for maintenance proton pump inhibitor (PPI) users as expressed as standardised incidence ratios (SIRs) and 95% confidence intervals (CIs)

(**a**) risk of colorectal adenocarcinomas

Indications for maintenance of PPI use	Colorectal adenocarcinoma		
	Number of cases (‰ in maintenance PPI users)	SIR (95 % CI)	Forest Plot
Without any below indication	879 (4.7)	1.13 (1.06-1.21)	
Helicobacter pylori infection or eradication	328 (5.9)	1.29 (1.15-1.44)	
Gastroesophageal reflux	1,352 (7)	1.26 (1.19-1.33)	
Barrett's oesophagus	39 (6.8)	1.16 (0.83-1.59)	
Peptic ulcer	625 (8.3)	1.16 (1.07-1.26)	
Zollinger Ellison's syndrome	0	-	
Gastro-duodenitis	739 (7.4)	1.28 (1.19-1.38)	
Dyspepsia	368 (8.8)	1.29 (1.16-1.42)	
Maintenance use of low-dose aspirin	2,026 (7.7)	1.00 (0.96-1.05)	
Maintenance use of non-steroidal anti-inflammatory drugs	1,096 (4.7)	1.00 (0.94-1.06)	
With any of the above indications	3,553 (6.3)	1.09 (1.05-1.12)	

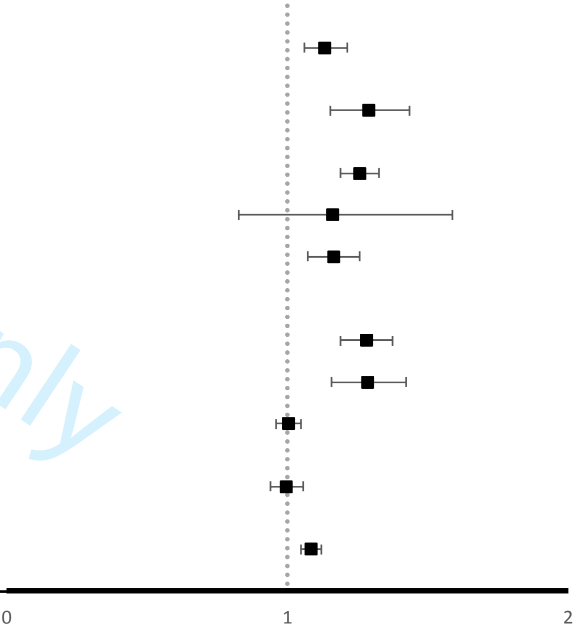

For peer review only

(**b**) risk of right-sided and left-sided colorectal adenocarcinomas

Indications for maintenance of PPI use	Right-sided colorectal adenocarcinoma		Left-sided colorectal adenocarcinoma	
	Number of cases (‰ in maintenance PPI users)	SIR (95 % CI)	Number of cases (‰ in maintenance PPI users)	SIR (95 % CI)
Without any below indication	360 (1.9)	1.27 (1.15-1.41)	511 (2.7)	1.05 (0.97-1.15)
Helicobacter pylori infection or eradication	148 (2.7)	1.63(1.37-1.91)	176 (3.2)	1.09 (0.94-1.27)
Gastroesophageal reflux	616 (3.2)	1.58 (1.45-1.70)	718 (3.7)	1.06 (0.99-1.15)
Barrett's oesophagus	17 (3)	1.53 (0.89-2.45)	21 (3.7)	0.95 (0.59-1.45)
Peptic ulcer	288 (3.8)	1.45 (1.28-1.62)	333 (4.4)	1.00 (0.90-1.11)
Zollinger Ellison's syndrome	0	-	0	-
Gastro-duodenitis	361 (3.6)	1.70 (1.53-1.88)	370 (3.7)	1.03 (0.93-1.14)
Dyspepsia	186 (4.4)	1.74 (1.50-2.01)	179 (4.3)	1.01 (0.87-1.17)
Maintenance use of low-dose aspirin	900 (3.4)	1.18 (1.10-1.26)	1,110 (4.2)	0.90 (0.85-0.95)
Maintenance use of non-steroidal anti-inflammatory drugs	397 (1.7)	0.98 (0.89-1.09)	685 (3)	1.00 (0.92-1.08)
With any of the above indications	1,524 (2.7)	1.26(1.19-1.32)	1,988 (3.5)	0.98 (0.94-1.02)

Forest plot

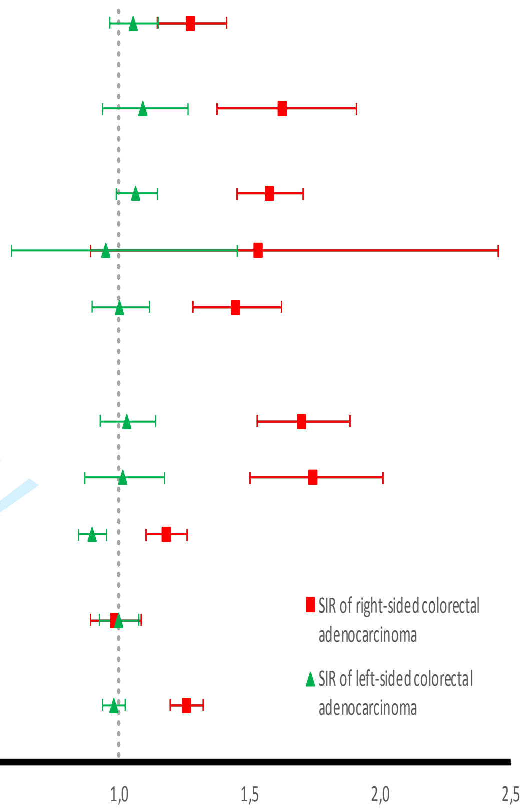

For peer review only

4. Duration of follow-up time

There was an elevated risk of CRA observed between the exposed population and the general population during the 1-2 years of follow-up (SIR 1.24, 95% CI 1.17-1.32). Thereafter, the results suggested slightly decreased risks of CRA in the 2-4 years (SIR 0.91, 95% CI 0.87-0.96), 4-6 years (SIR 0.65, 95% CI 0.61-0.68) and ≥6 years (SIR 0.85, 95% CI 0.77-0.92) of follow-up (Figure 1**a**). There was no obvious difference in CRA risks observed between the maintenance H_2RA users and the general population during the follow-up time (Figure 1**a**). An increased risk of right-sided CRA was also observed during the 1-2 years of follow-up (Figure 1**b**).

5. Comparison with maintenance H_2RA use

With a total of 19,795 individuals and 130,810 person-years of follow-up, the risk of CRA was not found to be associated with maintenance H_2RA use compared to the background population (N=115, SIR 0.85, 95% CI 0.71-1.03). The multivariable Poisson regression model of maintenance PPI versus H_2RA use showed an IRR of 1.05 (95% CI 0.87-1.27, P<0.05).

Discussion

In this large population-based study investigating the association between maintenance PPI use and CRC risk, we found a potential 10% higher risk of CRA compared with the general population. SIRs showed that maintenance PPI use might be associated with an increased risk of right-sided CRA compared with that in the general population. Young to middle-aged adult (i.e., 18-39 and 40-49 years old) participants showed a higher risk of CRA than the general population.

Our findings are in line with an estimated exposure effect of 2.03 (95% CI 1.56-2.63) between PPI users and CRC risk reported by a Taiwanese cohort(18), where immortal time bias could not be ruled out. In our study, we excluded 41,697 individuals with less than one year of follow-up to avoid detection bias. As CRA typically takes several to 20 years to develop from polyp to adenocarcinoma(44), cancer diagnoses or deaths within one year after new incident PPI prescriptions should be excluded because they could result in a biological effect indicating that PPI initiation preceded or increased the cancer risks. We have further carefully addressed the possibility that our strict exclusion of the first year of follow-up introduced immortal time bias. Therefore, we estimated the overall risk of CRA within the same study population starting follow-up from the first dispensed prescription date of PPIs. Supplementary Table S2 showed that the results were consistent with our main analysis, suggesting that immortal time bias might not be a problem; otherwise, the overestimates of healthy individuals should have led to an opposite observed effect.

In the duration analysis, maintenance PPI use was associated with an increased risk of CRA than the general population during the 1-2 years of follow-up. Following that, the exposed population showed a slightly lower risk of CRA than the general population. Then, the exposed population had a lower but less decreased risk of CRA compared to the general population with ≥6 years of follow-up. A similar pattern was observed for the risk of right-sided CRA. This may be because of potential confounding introduced by ever users who received maintenance PPI use long before the study period, as we do not have information on prescribed drug use prior to the start of the study (July 2005) when the National Prescribed Drug Registry was not fully constructed. A longer follow-up (i.e., more than 10 years) and more extensive wash-out of ever users may be needed, so that an increasing risk of CRA would be observed. Meanwhile, reverse causality (i.e., the observed effect explained by reversely linking initiating PPI to subclinical symptoms of CRC) should also be considered during the follow-up. If CRC patients were prone to be prescribed maintenance-used PPIs, substantial positive effects on the exposure estimation might have influenced each stratum of the exposed population during our analysis. However, the different trends of right- vs left-sided CRC risk during different time periods (Figure 1**b**) and with different indications (Table 4**b**) indicated that the reverse causality due to the prevalent use of maintenance PPIs might be less concerning.

Among the indication subgroup analyses, only maintenance PPI users with the combined use of NSAIDs or low-dose aspirin showed a potential preventive effect against colorectal tumour progression. A previous American cohort study concluded that long-term use of NSAIDs and low-dose aspirin reduced the risk of CRC(45). Our study also provided evidence that long-term NSAID or low-dose aspirin use appeared to be beneficial in concomitant use with PPIs. Nevertheless, an increased risk of CRA was observed among the *Helicobacter pylori* infection or eradication, gastroesophageal reflux, gastro-duodenitis, and dyspepsia groups, which have not been established as the risk factors for CRA. The elevated risk could be explained by the fact that acid-related disorders facilitate changes in the upper-intestinal microbiome barrier owing to gastric acid over secretion(46,47). Gut dysbiosis and hypergastrinaemia may also contribute to the association between PPI use and colorectal carcinogenesis(15,48,49)].

The strengths of our study include not only the high number of individuals and person-years but also the well-designed method for calculating cancer risks. The high-quality, high-coverage national registries allowed the identification of eligible individuals with a lengthy follow-up. Compared to previous studies, we constructed this cohort with many individuals and long follow-up times to minimise time-related bias. Moreover, the results are generalisable to other Nordic nations because comparable recording systems are maintained in other Nordic nations. The method enables us to compare large populations with different levels without considering migrations and drop-offs. In addition, we accounted for an alternative indicator of H_2RA to reduce the residual

confounding caused by PPI indications, which could lead to bidirectional results in the association between the exposure and outcome.

Our study has certain limitations, including a lack of additional data on lifestyle risk factors such as smoking, alcohol, diet, and body mass index. Moreover, although we followed the participants up to a maximum duration of 7.5 years, a median follow-up time of 5.3 years may be insufficient. A longer median follow-up permits a more extended observation of all naturally evoked outcomes and a more extensive exclusion of prevalent use without harming the sample size and power too much. Nevertheless, during the long progression period, the clinical diagnosis of CRA can be delayed following the onset of cancerous symptoms, which will dilute the association between the PPI and CRC risk.

Inappropriate PPI prescriptions are increasing among adults(50,51). Meanwhile, early onset CRC (diagnosed at <50 years of age) has been reported to be rising, especially as young populations at increased risk of CRC tend to have an unhealthy diet, a lack of exercise, obesity and a low acceptance of colonoscopy(52–54). The message to clinics is that young to middle-aged people should pay attention to the potential risk of CRA associated with maintenance PPI use. Moreover, combining low-dose aspirin or other NSAIDs with maintenance PPI treatment could be meaningful to PPI users with common indications, although further studies are needed before any firm conclusions can be made. Finally, right-sided CRA is more clinically insidious, more advanced at diagnosis, and worse in prognosis than left-sided CRA(30,55,56). Early chemoprevention for maintenance PPI users should be considered to prevent poorer outcomes of right-sided CRA because of the significantly increased risk of right-sided CRA following maintenance PPI use observed in our study than the general population.

In conclusion, our study showed that maintenance PPI use may be associated with an increased risk of CRA. Nevertheless, we need more population-based studies with extended observation to confirm the association.

Acknowledgement

We wish to address our gratitude to the Swedish National Board of Health and Welfare (Socialstyrelsen) for collecting all the data and to all the participants who contributed to the data collection.

Declaration of interest

The authors declare no conflict of interest.

Funding

This study was sponsored by Svenska Läkaresallskapet (SLS-788731, SLS-788751, SLS-783091) and the Swedish Research Council (2020-01058). QL was supported by

a China Scholarship Council Grant (201700260302). None of these sponsors had any role in the study design, the collection, the analysis, or the interpretation of data.

Author Contribution Statement

All authors designed the study and approved the final version of the manuscript (QL, XW, LE, OS, KF, NB). QL drafted the protocol, conducted the statistical analysis, and wrote the draft of the manuscript, which was revised and approved by all the other authors. NB was the guarantor of the study. QL and NB had full access to all the data and codes in the study.

Data availability statement

The datasets analysed during the current study are not publicly available due to the sharing agreement at Karolinska Institutet and the National Board of Health and Welfare but are available from the corresponding authors on reasonable request.

For peer review only

References:

1. Shin JM, Sachs G. Pharmacology of proton pump inhibitors. Vol. 10, Current Gastroenterology Reports. NIH Public Access; 2008. p. 528–34.
2. Yang YX, Hennessy S, Propert K, Hwang WT, Sedarat A, Lewis JD. Chronic Proton Pump Inhibitor Therapy and the Risk of Colorectal Cancer. Gastroenterology. 2007 Sep 1;133(3):748–54.
3. Hálfdánarson ÓÖ, Pottegård A, Björnsson ES, Lund SH, Ogmundsdottir MH, Steingrímsson E, et al. Proton-pump inhibitors among adults: a nationwide drug-utilization study. Therap Adv Gastroenterol. 2018 Jan 1;11.
4. Lassalle M, Le Tri T, Bardou M, Biour M, Kirchgesner J, Rouby F, et al. Use of proton pump inhibitors in adults in France: a nationwide drug utilization study. Eur J Clin Pharmacol. 2020;76(3):449–57.
5. Freedberg DE, Kim LS, Yang YX. The Risks and Benefits of Long-term Use of Proton Pump Inhibitors: Expert Review and Best Practice Advice From the American Gastroenterological Association. Gastroenterology. 2017 Mar 1;152(4):706–15.
6. Katz PO, Gerson LB, Vela MF. Guidelines for the diagnosis and management of gastroesophageal reflux disease. American Journal of Gastroenterology. 2013;108(3):308–28.
7. Targownik LE, Fisher DA, Saini SD. AGA Clinical Practice Update on De-Prescribing of Proton Pump Inhibitors: Expert Review. Gastroenterology. 2022 Apr 1;162(4):1334–42.
8. Kurlander JE, Gonzalez JJ, Saini SD. Deprescribing Proton Pump Inhibitors. JAMA Intern Med. 2020;180(12):1711–2.
9. Brusselaers N, Engstrand L, Lagergren J. Maintenance proton pump inhibition therapy and risk of oesophageal cancer. Cancer Epidemiol. 2018 Apr 1;53(November 2017):172–7.
10. Schneider JL, Kolitsopoulos F, Corley DA. Risk of gastric cancer, gastrointestinal cancers and other cancers: A comparison of treatment with pantoprazole and other proton pump inhibitors. Aliment Pharmacol Ther. 2016 Jan 1;43(1):73–82.
11. Brusselaers N, Sadr-Azodi O, Engstrand L. Long-term proton pump inhibitor usage and the association with pancreatic cancer in Sweden. J Gastroenterol. 2020 Apr 1;55(4):453–61.
12. Imhann F, Bonder MJ, Vila AV, Fu J, Mujagic Z, Vork L, et al. Proton pump inhibitors affect the gut microbiome. Gut. 2016 May;65(5):740–8.
13. Vich Vila A, Collij V, Sanna S, Sinha T, Imhann F, Bourgonje AR, et al. Impact of commonly used drugs on the composition and metabolic function of the gut microbiota. Nature Communications 2020 11:1. 2020 Jan 17;11(1):1–11.
14. Dekkers KF, Sayols-Baixeras S, Baldanzi G, Nowak C, Hammar U, Nguyen D, et al. An online atlas of human plasma metabolite signatures of gut microbiome composition. Nature Communications 2022 13:1. 2022 Sep 23;13(1):1–12.

For peer review only

15. Bruno G, Zaccari P, Rocco G, Scalese G, Panetta C, Porowska B, et al. Proton pump inhibitors and dysbiosis: Current knowledge and aspects to be clarified. World J Gastroenterol. 2019 Jun 6;25(22):2706.
16. Chen CC, Liou JM, Lee YC, Hong TC, El-Omar EM, Wu MS. The interplay between Helicobacter pylori and gastrointestinal microbiota. Gut Microbes. 2021;13(1):1–22.
17. Srivastava A, Gupta J, Kumar S, Kumar A. Gut biofilm forming bacteria in inflammatory bowel disease. Vol. 112, Microbial Pathogenesis. Academic Press; 2017. p. 5–14.
18. Lei WY, Wang JH, Yi CH, Liu TT, Hung JS, Wong MW, et al. Association between use of proton pump inhibitors and colorectal cancer: A nationwide population-based study. Clin Res Hepatol Gastroenterol. 2021 Jan 1;45(1):101397.
19. Lai SW, Liao KF, Lai HC, Lin CL, Sung FC. Use of proton pump inhibitors correlates with increased risk of colorectal cancer in Taiwan. Asia Pac J Clin Oncol. 2013 Jun 1;9(2):192–3.
20. Robertson DJ, Larsson H, Friis S, Pedersen L, Baron JA, Sørensen HT. Proton Pump Inhibitor Use and Risk of Colorectal Cancer: A Population-Based, Case-Control Study. Gastroenterology. 2007 Sep 1;133(3):755–60.
21. Yang YX, Hennessy S, Propert K, Hwang WT, Sedarat A, Lewis JD. Chronic Proton Pump Inhibitor Therapy and the Risk of Colorectal Cancer. Gastroenterology. 2007 Sep 1;133(3):748–54.
22. Chubak J, Boudreau DM, Rulyak SJ, Mandelson MT. Colorectal cancer risk in relation to use of acid suppressive medications. Pharmacoepidemiol Drug Saf. 2009 Jul 1;18(7):540–4.
23. Van Soest EM, Van Rossum LGM, Dieleman JP, Van Oijen MGH, Siersema PD, Sturkenboom MCJM, et al. Proton pump inhibitors and the risk of colorectal cancer. Am J Gastroenterol. 2008 Apr;103(4):966–73.
24. Lee JK, Merchant SA, Schneider JL, Jensen CD, Fireman BH, Quesenberry CP, et al. Proton pump inhibitor use and risk of gastric, colorectal, liver, and pancreatic cancers in a community-based population. American Journal of Gastroenterology. 2020;115(5):706–15.
25. Kuiper JG, Herk-Sukel MPP, Lemmens VEPP, Kuipers EJ, Herings RMC. Proton pump inhibitors are not associated with an increased risk of colorectal cancer. GastroHep. 2020 Jul;2(4):165–70.
26. Abrahami D, McDonald EG, Schnitzer ME, Barkun AN, Suissa S, Azoulay L. Proton pump inhibitors and risk of colorectal cancer. Gut. 2022;71(1):111–8.
27. Lai SW, Liao KF, Lai HC, Lin CL, Sung FC. Use of proton pump inhibitors correlates with increased risk of colorectal cancer in Taiwan. Asia Pac J Clin Oncol. 2013 Jun 1;9(2):192–3.
28. Hwang IC, Chang J, Park SM. Emerging hazard effects of proton pump inhibitor on the risk of colorectal cancer in low-risk populations: A Korean nationwide prospective cohort study. PLoS One. 2017 Dec 1;12(12):e0189114.

For peer review only

29. Thrumurthy SG, Thrumurthy SSD, Gilbert CE, Ross P, Haji A. Colorectal adenocarcinoma: risks, prevention and diagnosis. BMJ [Internet]. 2016 Jul 14 [cited 2022 Oct 4];354. Available from: https://www-bmj-com.proxy.kib.ki.se/content/354/bmj.i3590
30. Baran B, Mert Ozupek N, Yerli Tetik N, Acar E, Bekcioglu O, Baskin Y. Difference Between Left-Sided and Right-Sided Colorectal Cancer: A Focused Review of Literature. Gastroenterology Res. 2018;11(4):264–73.
31. Strand DS, Kim D, Peura DA. 25 Years of Proton Pump Inhibitors: A Comprehensive Review. Gut Liver. 2017 Jan 1;11(1):27.
32. Socialstyrelsen. The National Patient Register - Socialstyrelsen [Internet]. 2019 [cited 2020 Oct 29]. Available from: https://www.socialstyrelsen.se/en/statistics-and-data/registers/register-information/the-national-patient-register/
33. Swedish Cancer Registry Quality [Internet]. Available from: https://www.socialstyrelsen.se/en/statistics-and-data/registers/register-information/swedish-cancer-register/
34. Ludvigsson JF, Almqvist C, Bonamy AKE, Ljung R, Michaëlsson K, Neovius M, et al. Registers of the Swedish total population and their use in medical research. Eur J Epidemiol. 2016 Feb 1;31(2):125–36.
35. Brooke HL, Talbäck M, Hörnblad J, Johansson LA, Ludvigsson JF, Druid H, et al. The Swedish cause of death register. Eur J Epidemiol. 2017 Sep 5;32(9):765–73.
36. Wallerstedt SM, Wettermark B, Hoffmann M. The First Decade with the Swedish Prescribed Drug Register – A Systematic Review of the Output in the Scientific Literature. Basic Clin Pharmacol Toxicol. 2016 Nov 24;119(5):464–9.
37. von Elm E, Altman DG, Egger M, Pocock SJ, Gøtzsche PC, Vandenbroucke JP. The Strengthening the Reporting of Observational Studies in Epidemiology (STROBE) statement: guidelines for reporting observational studies. The Lancet. 2007 Oct 20;370(9596):1453–7.
38. FASS General - Home [Internet]. [cited 2021 Feb 2]. Available from: https://www.fass.se/LIF/startpage
39. Barlow L, Westergren K, Holmberg L, Talbäck M. The completeness of the Swedish Cancer Register – a sample survey for year 1998. Acta Oncol (Madr). 2009 Jan 8;48(1):27–33.
40. Vogel JD, Eskicioglu C, Weiser MR, Feingold DL, Steele SR. The American society of colon and rectal surgeons clinical practice guidelines for the treatment of colon cancer. Dis Colon Rectum. 2017;60(10):999–1017.
41. Brusselaers N, Lagergren J. Maintenance use of non-steroidal anti-inflammatory drugs and risk of gastrointestinal cancer in a nationwide population-based cohort study in Sweden. BMJ Open. 2018;8(7).
42. D. Machin , N. E. Breslow NED. Statistical Methods in Cancer Research, Vol. II: The Design and Analysis of Cohort Studies. International Biometric Society. 1990. Vol. 46, No. 4 (Dec., 1990), pp. 1243–1244.
43. Bender R. Introduction to the use of regression models in epidemiology. Methods in Molecular Biology. 2009;471:179–95.

For peer review only

44. Simon K. Colorectal cancer development and advances in screening. Clin Interv Aging. 2016;11:967–76.
45. Chan AT, Giovannucci EL, Meyerhardt JA, Schernhammer ES, Curhan GC, Fuchs CS. Long-term use of aspirin and nonsteroidal anti-inflammatory drugs and risk of colorectal cancer. JAMA. 2005 Aug 1;294(8):914–23.
46. Zhong L, Shanahan ER, Raj A, Koloski NA, Fletcher L, Morrison M, et al. Dyspepsia and the microbiome: time to focus on the small intestine. Vol. 66, Gut. England; 2017. p. 1168–9.
47. Minalyan A, Gabrielyan L, Scott D, Jacobs J, Pisegna JR. The Gastric and Intestinal Microbiome: Role of Proton Pump Inhibitors. Curr Gastroenterol Rep. 2017 Aug;19(8):42.
48. Macke L, Schulz C, Koletzko L, Malfertheiner P. Systematic review: the effects of proton pump inhibitors on the microbiome of the digestive tract-evidence from next-generation sequencing studies. Aliment Pharmacol Ther. 2020 Mar;51(5):505–26.
49. Robertson DJ, Sandler RS, Ahnen DJ, Greenberg ER, Mott LA, Cole BF, et al. Gastrin, Helicobacter pylori, and Colorectal Adenomas. Clinical Gastroenterology and Hepatology. 2009 Feb;7(2):163–7.
50. Calvo LLJ, García Cámara P, Llorente Barrio M, Sierra Gabarda O, Monzón Baez R, Arbonés Mainar JM, et al. Successful deprescribing of proton pump inhibitors with a patient-centered process: the DESPIBP Project. Eur J Clin Pharmacol. 2021;77(12):1927–33.
51. Muheim L, Signorell A, Markun S, Chmiel C, Neuner-Jehle S, Blozik E, et al. Potentially inappropriate proton-pump inhibitor prescription in the general population: a claims-based retrospective time trend analysis. Therap Adv Gastroenterol. 2021 Jun;14(6):1756284821998928.
52. Brenner DR, Ruan Y, Shaw E, De P, Heitman SJ, Hilsden RJ. Increasing colorectal cancer incidence trends among younger adults in Canada. Prev Med. 2017 Dec 1;105:345–9.
53. Vuik FER, Nieuwenburg SAV, Bardou M, Lansdorp-Vogelaar I, Dinis-Ribeiro M, Bento MJ, et al. Increasing incidence of colorectal cancer in young adults in Europe over the last 25 years. Gut. 2019;
54. Potter JD. Rising rates of colorectal cancer in younger adults. BMJ. 2019 Jun 24;365.
55. Meguid RA, Slidell MB, Wolfgang CL, Chang DC, Ahuja N. Is there a difference in survival between right- versus left-sided colon cancers? Ann Surg Oncol. 2008 Sep;15(9):2388–94.
56. Mik M, Berut M, Dziki L, Trzcinski R, Dziki A. Right-and left-sided colon cancer-clinical and pathological differences of the disease entity in one organ. Archives of Medical Science. 2017 Feb 1;13(1):157–62.

For peer review only

Figure 1. Duration of follow-up and risk of colorectal adenocarcinomas among (**a**) maintenance proton pump inhibitor (PPI) users and maintenance histamine-2 receptor antagonist (H_2RA) users (**b**) overall maintenance PPI users and users diagnosed with right-sided or left-sided colorectal adenocarcinomas, expressed as standardised incidence ratios (SIRs) and 95% confidence intervals (CIs)

(**a**) maintenance PPI users and H_2RA users

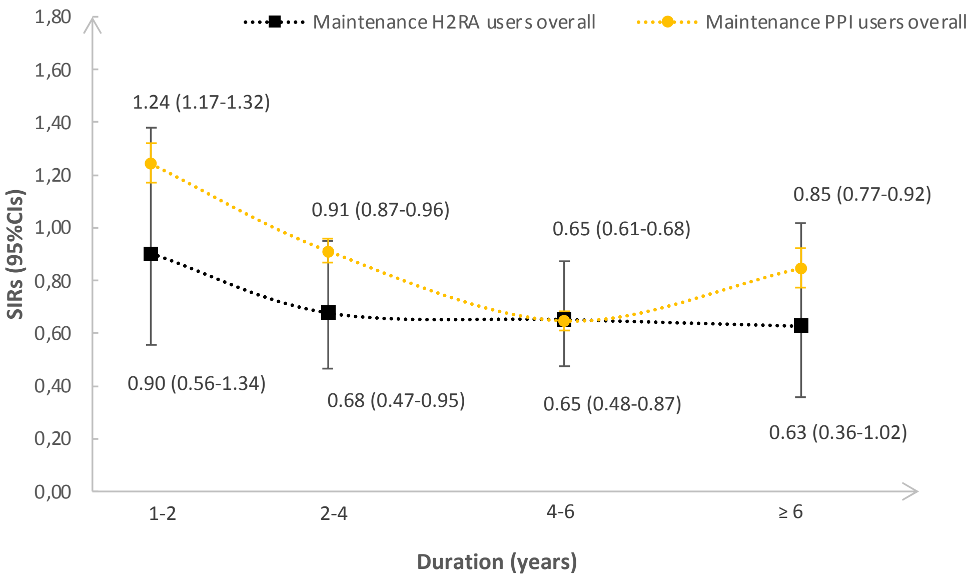

(**b**) overall maintenance PPI users and users diagnosed with right-sided or left-sided colorectal adenocarcinomas

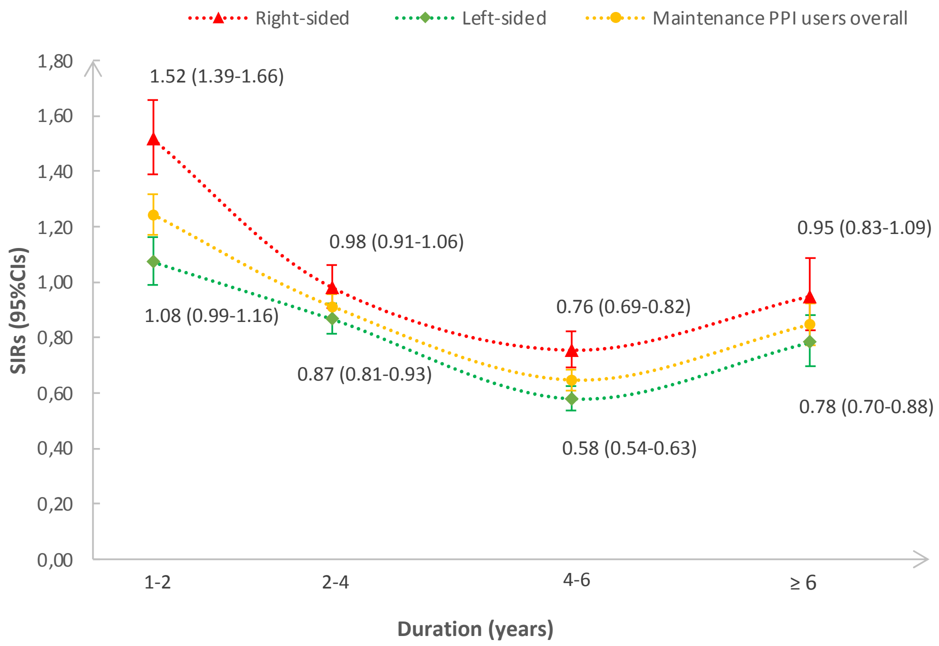

Supplementary Materials:

Table S1. International Classification of Diseases code for oncology and Anatomical Therapeutic Chemical codes used in this study

Table S2. Risk of colorectal adenocarcinomas in maintenance proton pump inhibitor (PPI) users followed up from enrolment, expressed as standardised incidence ratios (SIRs) and 95% confidence intervals (CIs) by age, sex, calendar period, tumour subsites and stages

Figure S1. Flow chart illustrating the enrolment of the study population of (**a**) maintenance proton pump inhibitor (PPI) users (**b**) maintenance histamine-2 receptor antagonist (H_2RA) users

For peer review only

Table S1. International Classification of Diseases code for oncology and Anatomical Therapeutic Chemical codes used in this study

Diseases	International Classification of Diseases code for oncology, 10th edition	Dispensed drugs	Anatomical Therapeutic Chemical code
Colorectal cancer	C18 -C20	Proton pump inhibitor	A02BC
Adenocarcinoma	096	Non-steroidal anti-inflammatory drug	M01A
Right-sided colorectal cancer	C18.0 -C18.4	Low-dose aspirin	B01AC06
Left-sided colorectal cancer	C18.5 - C18.7, C19, C20	Histamine-2 receptor antagonist	A02BA
Overlapping or unspecified sites of colorectal cancer	C18.8 C18.9		

For peer review only

Table S2. Risk of colorectal adenocarcinomas in maintenance proton pump inhibitor (PPI) users followed from enrolment, expressed as standardised incidence ratios (SIRs) and 95% confidence intervals (CIs) by age, sex, calendar period, tumour subsites and stages

	Maintenance PPI users with maximum follow-up time	
	Number	SIRs (95% CI)
Total colorectal adenocarcinomas	6,887	1.44 (1.40-1.47)
Sex		
Men	3,341	1.48 (1.43-1.53)
Women	3,546	1.40 (1.35-1.44)
Age at starting the maintenance PPI use, years		
18-39	67	5.53 (4.03-7.39)
40-49	218	2.75 (2.35-3.19)
50-59	775	1.78 (1.64-1.94)
60-69	1,870	1.60 (1.52-1.68)
≥70	3,957	1.32 (1.28-1.36)
Calendar year period		
2005-2006	4,126	1.76 (1.65-1.88)
2007-2009	1,982	1.55 (1.49-1.60)
2010-2012	779	1.28 (1.24-1.33)
Subsites of colorectal adenocarcinoma		
Right-sided	3,043	1.74 (1.68-1.81)
Left-sided	3,710	1.25 (1.21-1.29)
Stages of colorectal adenocarcinoma		
Stage 0-I	966	1.49 (1.40-1.59)
Stage II	1,657	1.41 (1.34-1.48)
Stage III	1,561	1.40 (1.33-1.47)
Stage IV	1,172	1.27 (1.19-1.34)
Missing	1,531	1.62 (1.54-1.70)
Follow-up time, years (median)	6.1	-

For peer review only

Figure S1. Flow chart illustrating the enrolment of the study population of (**a**) maintenance proton pump inhibitor (PPI) users (**b**) maintenance histamine-2 receptor antagonist (H_2RA) users

(**a**) maintenance PPI users

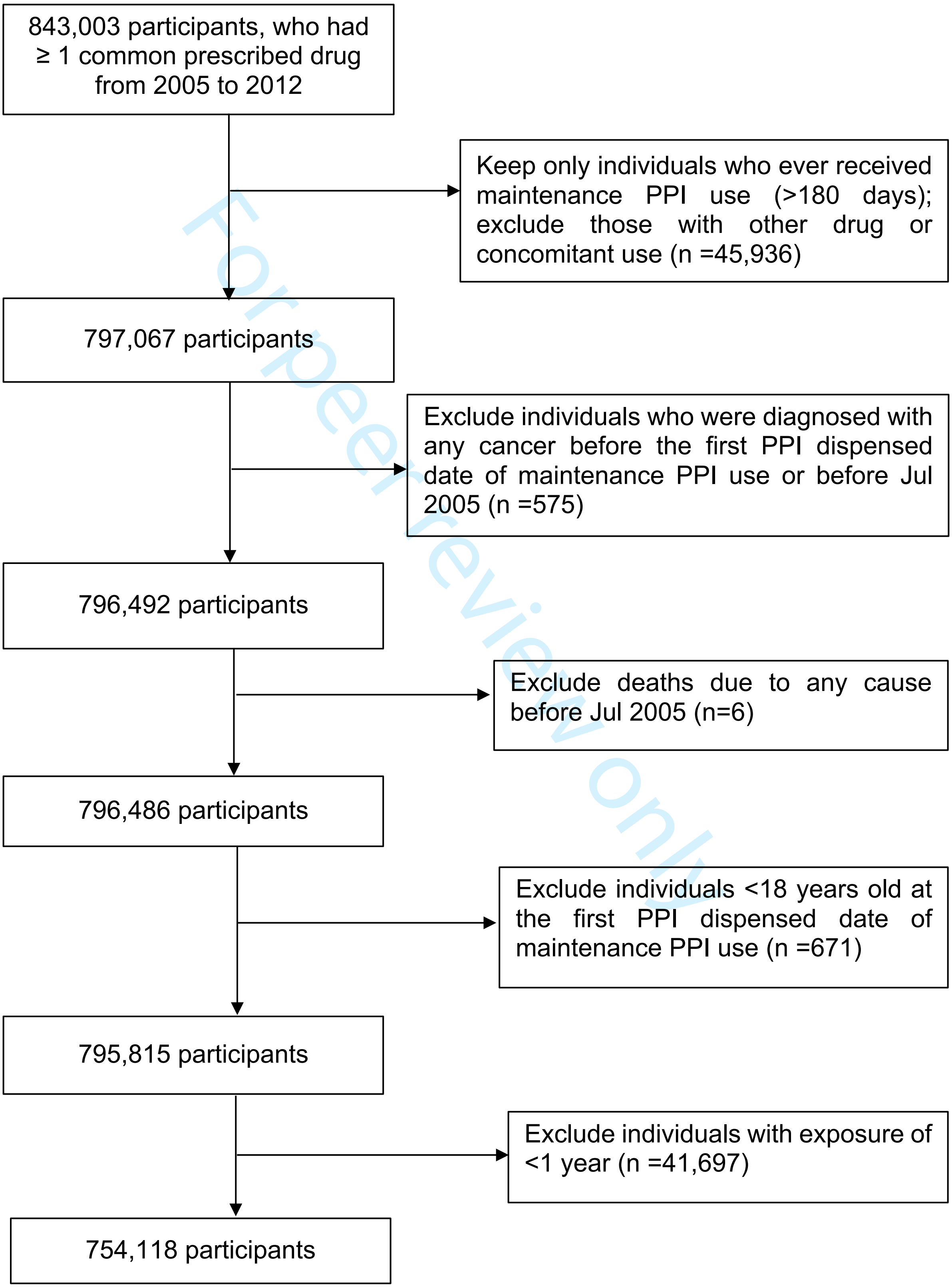

For peer review only

(**b**) maintenance H_2RA users

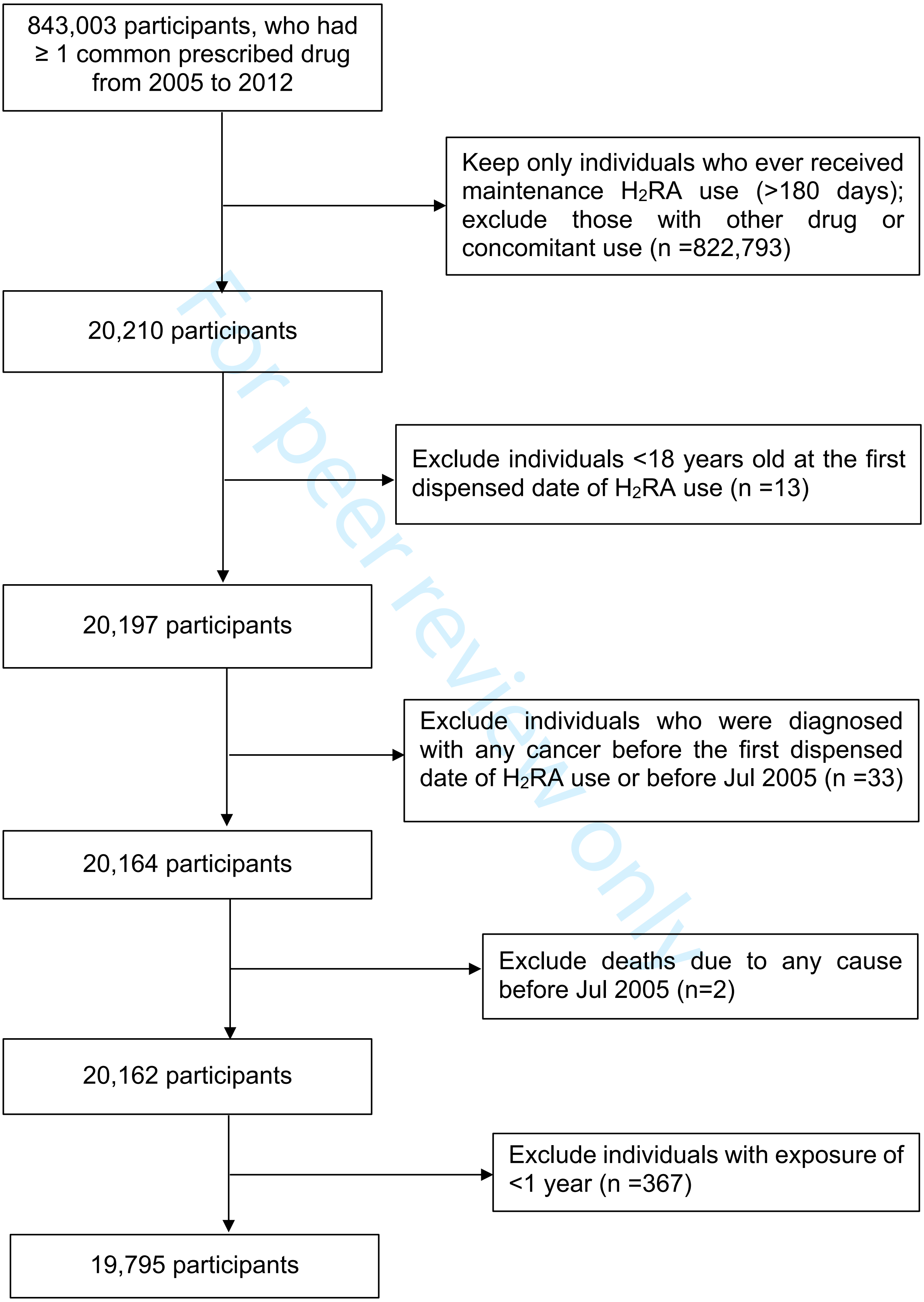

For peer review only

STROBE Statement—Checklist of items that should be included in reports of ***cohort studies***

	Item No	Recommendation	Page No
**Title and abstract**	1	(*a*) Indicate the study's design with a commonly used term in the title or the abstract<br>(*b*) Provide in the abstract an informative and balanced summary of what was done and what was found	1,2
**Introduction**			
Background/rationale	2	Explain the scientific background and rationale for the investigation being reported	4
Objectives	3	State specific objectives, including any prespecified hypotheses	4
**Methods**			
Study design	4	Present key elements of study design early in the paper	4,5
Setting	5	Describe the setting, locations, and relevant dates, including periods of recruitment, exposure, follow-up, and data collection	5
Participants	6	(*a*) Give the eligibility criteria, and the sources and methods of selection of participants. Describe methods of follow-up<br>(*b*) For matched studies, give matching criteria and number of exposed and unexposed	5
Variables	7	Clearly define all outcomes, exposures, predictors, potential confounders, and effect modifiers. Give diagnostic criteria, if applicable	5
Data sources/ measurement	8*	For each variable of interest, give sources of data and details of methods of assessment (measurement). Describe comparability of assessment methods if there is more than one group	5
Bias	9	Describe any efforts to address potential sources of bias	3,4,5
Study size	10	Explain how the study size was arrived at	5
Quantitative variables	11	Explain how quantitative variables were handled in the analyses. If applicable, describe which groupings were chosen and why	5,6
Statistical methods	12	(*a*) Describe all statistical methods, including those used to control for confounding<br>(*b*) Describe any methods used to examine subgroups and interactions<br>(*c*) Explain how missing data were addressed<br>(*d*) If applicable, explain how loss to follow-up was addressed<br>(*e*) Describe any sensitivity analyses	6,7
**Results**			
Participants	13*	(a) Report numbers of individuals at each stage of study—eg numbers potentially eligible, examined for eligibility, confirmed eligible, included in the study, completing follow-up, and analysed<br>(b) Give reasons for non-participation at each stage<br>(c) Consider use of a flow diagram	7
Descriptive data	14*	(a) Give characteristics of study participants (eg demographic, clinical, social) and information on exposures and potential confounders<br>(b) Indicate number of participants with missing data for each variable of interest<br>(c) Summarise follow-up time (eg, average and total amount)	7-13
Outcome data	15*	Report numbers of outcome events or summary measures over time	13

For peer review only - http://bmjopen.bmj.com/site/about/guidelines.xhtml

Main results	16	(*a*) Give unadjusted estimates and, if applicable, confounder-adjusted estimates and their precision (eg, 95% confidence interval). Make clear which confounders were adjusted for and why they were included<br>(*b*) Report category boundaries when continuous variables were categorized<br>(*c*) If relevant, consider translating estimates of relative risk into absolute risk for a meaningful time period	7-13
Other analyses	17	Report other analyses done—eg analyses of subgroups and interactions, and sensitivity analyses	13
**Discussion**			
Key results	18	Summarise key results with reference to study objectives	14
Limitations	19	Discuss limitations of the study, taking into account sources of potential bias or imprecision. Discuss both direction and magnitude of any potential bias	16
Interpretation	20	Give a cautious overall interpretation of results considering objectives, limitations, multiplicity of analyses, results from similar studies, and other relevant evidence	14,15
Generalisability	21	Discuss the generalisability (external validity) of the study results	15
**Other information**			
Funding	22	Give the source of funding and the role of the funders for the present study and, if applicable, for the original study on which the present article is based	16,17

*Give information separately for exposed and unexposed groups.

Note: An Explanation and Elaboration article discusses each checklist item and gives methodological background and published examples of transparent reporting. The STROBE checklist is best used in conjunction with this article (freely available on the Web sites of PLoS Medicine at http://www.plosmedicine.org/, Annals of Internal Medicine at http://www.annals.org/, and Epidemiology at http://www.epidem.com/). Information on the STROBE Initiative is available at http://www.strobe-statement.org.

For peer review only

For peer review only - http://bmjopen.bmj.com/site/about/guidelines.xhtml

BMJ Open

Maintenance proton pump inhibitor use and risk of colorectal cancer: A Swedish retrospective cohort study

Journal:	*BMJ Open*
Manuscript ID	bmjopen-2023-079591.R1
Article Type:	Original research
Date Submitted by the Author:	17-Apr-2024
Complete List of Authors:	Liu, Qing; Karolinska Institutet, Department of Microbiology, Tumour and Cell Biology<br>WANG, Xinchen; Karolinska Institutet, Department of Clinical Neuroscience<br>Engstrand, Lars; Karolinska Institutet, Microbiology, Tumour and Cell Biology<br>Sadr-Azodi, Omid; Karolinska Institutet<br>Fall, Katja; Karolinska Institutet, Dept of Medical Epidemiology and Biostatistic<br>Brusselaers, Nele; Karolinska Institutet, Molecular medicine and Surgery
**Primary Subject Heading**:	Epidemiology
Secondary Subject Heading:	Oncology, Pharmacology and therapeutics, Public health, Gastroenterology and hepatology
Keywords:	CLINICAL PHARMACOLOGY, EPIDEMIOLOGIC STUDIES, GASTROENTEROLOGY, Gastrointestinal tumours < GASTROENTEROLOGY, REGISTRIES, Risk Factors

SCHOLARONE™
Manuscripts

I, the Submitting Author has the right to grant and does grant on behalf of all authors of the Work (as defined in the below author licence), an exclusive licence and/or a non-exclusive licence for contributions from authors who are: i) UK Crown employees; ii) where BMJ has agreed a CC-BY licence shall apply, and/or iii) in accordance with the terms applicable for US Federal Government officers or employees acting as part of their official duties; on a worldwide, perpetual, irrevocable, royalty-free basis to BMJ Publishing Group Ltd ("BMJ") its licensees and where the relevant Journal is co-owned by BMJ to the co-owners of the Journal, to publish the Work in this journal and any other BMJ products and to exploit all rights, as set out in our licence.

The Submitting Author accepts and understands that any supply made under these terms is made by BMJ to the Submitting Author unless you are acting as an employee on behalf of your employer or a postgraduate student of an affiliated institution which is paying any applicable article publishing charge ("APC") for Open Access articles. Where the Submitting Author wishes to make the Work available on an Open Access basis (and intends to pay the relevant APC), the terms of reuse of such Open Access shall be governed by a Creative Commons licence – details of these licences and which Creative Commons licence will apply to this Work are set out in our licence referred to above.

Other than as permitted in any relevant BMJ Author's Self Archiving Policies, I confirm this Work has not been accepted for publication elsewhere, is not being considered for publication elsewhere and does not duplicate material already published. I confirm all authors consent to publication of this Work and authorise the granting of this licence.

For peer review only

Title: Maintenance proton pump inhibitor use and risk of colorectal cancer: A Swedish retrospective cohort study

Authors:
Qing LIU [1*], Xinchen WANG [2], Lars ENGSTRAND [1], Omid SADR-AZODI [3,4], Katja FALL[5,6], Nele BRUSSELAERS [1,7,8*]

Affiliations:
1 Centre for Translational Microbiome Research, Department of Microbiology, Tumour and Cell Biology, Karolinska Institutet, Stockholm, Sweden
2 Centre for Psychiatry Research, Department of Clinical Neuroscience, Karolinska Institutet, Stockholm, Sweden
3 Department of Clinical Sciences, Intervention and Technology, Karolinska Institutet, Stockholm, Sweden
4 Department of Surgery, Capio Saint Göran Hospital, Stockholm, Sweden
5 Clinical Epidemiology and Biostatistics School of Medical Sciences, Örebro University, Örebro, Sweden
6 Institute of Environmental Medicine, Karolinska Institutet, Stockholm, Sweden
7 Department of Head and Skin, Ghent University, Ghent, Belgium
8 Global Health Institute, Antwerp University, Antwerp, Belgium

Corresponding author: Qing Liu, qing.liu2017@outlook.com. Centre for Translational Microbiome Research, Department of Microbiology, Tumour and Cell biology, Karolinska Institutet. Biomedicum, kvarter 8a. Solnavägen 9, SE-171 65 Stockholm. Telephone: +46 (0) 8-524 845 97.

For peer review only

Abstract

Objectives: We aimed to evaluate the risk of colorectal adenocarcinoma (CRA) associated with long-term use of proton pump inhibitors (PPIs) in a large nationwide cohort.

Design: Retrospective cohort study.

Setting: This research was conducted at the national level, encompassing the entire population of Sweden.

Participants: This study utilized Swedish national registries to identify all adults who had ≥180 days of cumulative PPI use between July 2005 and December 2012, excluding participants who were followed up for less than one year. A total of 754,118 maintenance PPI users were included, with a maximum follow-up of 7.5 years.

Interventions: Maintenance PPI use (cumulative ≥180 days), with a comparator of maintenance histamine-2 receptor antagonist (H_2RA) use.

Primary and Secondary Outcome Measures: The primary outcome measure was the risk of CRA, presented as standardised incidence ratios (SIRs) with 95% confidence intervals (CIs). Subgroup analyses were performed to explore the impact of indications, tumour locations, tumour stages, and the duration of follow-up. A multivariable Poisson regression model was fitted to estimate the incidence rate ratios (IRRs) and 95% CIs of PPI versus H_2RA use.

Results: Maintenance PPI users exhibited a slightly elevated risk of CRA compared to the general population (SIR 1.10, 95% CI= 1.06-1.13) for both men and women. Individuals aged 18-39 (SIR 2.79, 95% CI=1.62-4.47) and 40-49 (SIR 2.02, 95% CI=1.65-2.45) had significantly higher risks than the general population. Right-sided CRA showed a higher risk compared to the general population (SIR 1.26, 95% CI=1.20-1.32). There was no significant difference in the risk of CRA between maintenance PPI users and maintenance H_2RA users (IRR 1.05, 95% CI=0.87-1.27, $P<0.05$).

Conclusions: Maintenance PPI use may be associated with an increased risk of CRA, but a prolonged observation time is needed.

Keywords: Cancer risk, Colorectal cancer, Proton pump inhibitors, Carcinogenesis, Histamine-2 receptor antagonist

Strengths and limitations of this study

- High number of enrolled individuals and person-years.
- High external validity and reduced residual confounding by the active comparator.
- Lack of additional data on some lifestyle risk factors.
- Potential confounding introduced by ever users who received maintenance PPI use long before the study period.
- Longer follow-up (i.e., more than 10 years) may be needed.

For peer review only

Introduction

Proton pump inhibitors (PPIs) are one of the most commonly prescribed drugs globally and are widely used for the treatment and prevention of various gastric acid-related disorders(1,2). The use of both prescription and over-the-counter PPIs has increased in many countries, with an estimated 10-30% of adults regularly using PPIs(3,4). Although PPIs have been deemed safe for short-term use and are recommended for a broad spectrum of indications by current guidelines and expert reviews(5–7), only a few indications justify long-term use exceeding 4 weeks. Despite this, PPIs are often overprescribed(7,8), which highlights the need for further investigation into the potential long-term side effects and public health implications of their widespread use.

Long-term PPI use has been associated with an increased risk of gastrointestinal cancers, including gastric, oesophageal and pancreatic cancers(9–11). Previous studies have demonstrated that PPI use significantly impacts the gut microbiome and contributes to dysbiosis(12–14), which has been linked to oncogenesis in the gut epithelium(15,16). Enteric infections and the trophic effects of PPI-induced hypergastrinaemia may also serve as potential cancerogenic factors(17). While some cohort studies have reported elevated relative risks of colorectal cancer (CRC) following PPI use(18,19), others have found no association between CRC and PPI use(20–26). However, potential methodological limitations in some of these studies have impacted the results. For example, some case-control studies introduced selection bias in exposure effect estimation by enrolling prevalent users rather than new users(22,23,25,27), and latency bias and time-window bias were present in some previous cohort studies due to the selection of narrow observation time ranges (19,22,28).

To clarify the relationship between PPI use and CRC, we conducted a large cohort study using high-coverage national registries to control for immortal time bias and time-window bias. We aimed to assess the association between maintenance PPI use and the risk of colorectal adenocarcinoma (CRA), which accounts for >95% of all CRC cases(29). We focused on right-sided and left-sided CRAs due to their chronological exposure to the drug-effects based on the anatomical locations(29,30). Additionally, we used the less popular histamine-2 receptor antagonists (H_2RAs) as active comparators in the supplementary analysis to minimise confounding-by-indication, as they share similar indications with PPIs and were used for treating gastric acid-related disorders before the introduction of PPIs as more potent acid suppressants(31).

Methods

Study design

This was a register-based cohort study that enrolled all Swedish adults (aged 18 years old) who were on maintenance PPI therapy between July 2005 and December 2012.

The study population was compared to the general population (i.e., the Swedish total population) stratified by the same age, sex, and calendar period. Participants were followed from one year after the first dispensed prescription date until the first diagnosis of CRA, death or the end of the study period (December 31, 2012). Data were obtained from the Swedish Cancer Registry, the National Patient Registry, the Cause of Death Registry, the Prescribed Drug Registry, and the Swedish Total Population Registry(32–36). Cancer and demographic information of the total population can be downloaded from the National Board of Health and Welfare's statistical database (32). The study protocol was established a priori, and the study was conducted in accordance with the Strengthening the Reporting of Observational Studies in Epidemiology (STROBE) statement(37).

Patient and public involvement

Due to its data-driven nature, this retrospective study had no patient or public directly involved in the study's design, data analysis, method selection or dissemination planning. Research questions and outcome measures were derived from existing literature and clinical relevance without direct input from them. Recruitment and follow-up relied on existing records from national registries.

Exposure

PPI usage data were obtained from the Prescribed Drug Registry, labelled by the (ATC)/defined daily dose (DDD) system. This system annotated the DDD per prescribed package with ATC codes. PPIs are also available over the counter in Sweden but only in small quantities and at exorbitant prices in comparison to prescribed PPIs(38). Therefore, most PPI consumption of long-term users is likely recorded in this registry. Maintenance PPI use (ATC code A02BC) was defined as cumulative use for at least 180 days prior to the onset of any cancer, death, or the end of the study period. Maintenance H_2RA (ATC code A02BA) use served as an active comparator, and it was defined as using H_2RA for at least 180 days before the onset of any cancer, death, or the end of the study period.

Outcome

The primary outcome was CRA, defined using the International Classification of Diseases (ICD, 10th edition) codes C18-C20 from the Swedish Cancer Registry(39), and the histopathology code 096. The tumours were further classified based on their anatomical location as right-sided, left-sided, or overlapping/unspecified sites (ICD codes in Supplementary Table S1). The tumour-node-metastasis (TNM) classification of the American Joint Committee on Cancer 8th edition Staging of Colorectal Cancer was used to define tumour stage and grouped as follows: stage 0-I (Tis, N0, M0 for stage 0; T1-2, N0, M0 for stage I), II (T3-4, N0, M0), III (T1-4, N1-2, M0), and IV (any T, any N, M1)(40).

For peer review only - http://bmjopen.bmj.com/site/about/guidelines.xhtml

Exclusions

Individuals with any documented cancer diagnosis prior to the study period or prior to the first dispensing date of PPIs were excluded. Individuals who switched between maintenance use of PPIs and H_2RAs (defined as ≥180 days of accumulated use of either drug) or had an exposure period of less than one year were also excluded. (Supplementary Figure S1)

Covariates

Age at treatment initiation was categorised into 18-39, 40-49, 50-59, 60-69 and ≥ 70 years old, and calendar periods were categorised into 2005-2006, 2007-2009, and 2010-2012. The duration of time since drug initiation was categorised as 1-2, 2-4, 4-6, and ≥ 6 years. No missing values for these variables were found for any of the study participants.

The most common recorded indications of maintenance PPI use were extracted from the National Patient Registry, including *Helicobacter pylori* infection or eradication, gastroesophageal reflux, Barrett's oesophagus, peptic ulcer, Zollinger Ellison's syndrome, gastro-duodenitis, dyspepsia, maintenance use (> 180 days during the study period) of low-dose aspirin, and maintenance use of NSAIDs(9,11,41) (ICD codes in Supplementary Table S1). We performed subgroup analyses on individuals with and without the listed indications, as well as on each indication separately when the statistical power was sufficient.

Statistical analyses

We calculated standardised incidence ratios (SIRs) with 95% confidence intervals (CIs) standardised by age, sex, and calendar period to assess the association between maintenance PPI use and CRA(42). The information on the incidence of CRA and the demographic characteristics of the total population can be downloaded from the National Board of Health and Welfare's statistical database (summarised in the Supplementary Table S2)(43). In our main analysis, we compared the overall risk of CRA in the study population to the general population of Sweden, stratifying the main outcomes by tumour stage (0-I, II, III, IV, and missing values), tumour location (right-sided, left-sided), indications, and follow-up time since drug initiation (1-2, 2-4, 4-6, and ≥ 6 years). We extensively investigated the risk of different tumour locations by age, sex, and indications for maintenance PPI use. Additionally, we assessed the risk of CRA in maintenance PPI users with a maximal follow-up time from the first dispensed prescription date, considering age, sex, calendar period, tumour stage, and tumour location.

Furthermore, we conducted two supplementary analyses. First, we estimated SIRs for maintenance H_2RA users and conducted a duration analysis (1-2, 2-4, 4-6, and ≥ 6 years). Second, we used a multivariable Poisson regression model to estimate

incidence rate ratios (IRRs) with 95% CIs to compare CRA risk between maintenance PPI users and H_2RA users, adjusting for age, sex and common therapy indications(44). The number of patients analysed for the Poisson regression model equals the summarized number of maintenance PPI users and maintenance H_2RA users. Propensity score adjustment between maintenance PPI users and H_2RA users was performed in the model to better control for confounders. Propensity scores were estimated using multivariable logistic regression, including age, sex, and indications. We applied *p* values less than 0.05 statistically significant for all the analyses. Stata MP 14.2 was used for all calculations.

Results

1. Characteristics of the PPI cohort

This study included 754,118 adult maintenance PPI users with no prior history of cancer, with a median follow-up of 5.3 years (totalling 4,177,396 person-years) and a maximum follow-up of 7.5 years. Women (0.52%) had a lower cumulative incidence of CRA than men (0.62%). Maintenance PPI use was most common in individuals aged 60-69 and ≥70 years old (Tables 1 & 2). A total of 4,432 cases of CRA were identified, with the majority being left-sided (N=2,499, 56.4%), stage II (N=1,102, 24.9%) and III (N=1,017, 22.9%).

Table 1. Characteristics of all maintenance proton pump inhibitor (PPI) users in Sweden between 2005 and 2012

Characteristics of the study population		Maintenance PPI users	
		Number	%
Total		754,118	100.0
Age at starting the maintenance PPI use, years			
	18-39 years	84,510	11.2
	40-49 years	99,897	13.3
	50-59 years	149,167	19.8
	60-69 years	167,456	22.2
	≥70 years	253,088	33.6
Sex			
	Men	310,357	41.2
	Women	443,761	58.9
Calendar period			
	2005-2006	430,527	57.1
	2007-2009	221,369	29.4
	2010-2012	102,222	13.6
Colorectal adenocarcinoma		4,432	0.6
	Right-sided	1,884	0.3
	Left-sided	2,499	0.3

Overlapping or unspecified	49	0.0
Tumour stages of colorectal adenocarcinoma		
Stage 0-I	674	0.1
Stage II	1,102	0.1
Stage III	1,017	0.1
Stage IV	748	0.1
Missing	891	0.1
Indications for maintenance PPI use		
Helicobacter pylori infection or eradication	55,311	7.3
Gastroesophageal reflux	193,371	25.6
Barrett's oesophagus	5,717	0.8
Peptic ulcer	74,946	10.0
Zollinger Ellison's syndrome	28	0.0
Gastro-duodenitis	100,191	13.2
Dyspepsia	41,916	5.6
Aspirin maintenance use	262,242	34.8
Non-steroidal anti-inflammatory drugs maintenance use	231,792	30.7
Without any of these recorded indications	186,529	24.7
Maintenance PPI use based on daily drug usage		
Interquartile range	1810	-
Person-years	3,426,925	-
Follow-up time, years (median)	5.3	-

Table 2. Risk of colorectal adenocarcinomas in maintenance proton pump inhibitor (PPI) users, expressed as standardised incidence ratios (SIRs) and 95% confidence intervals (CIs) by age, sex, calendar period, tumour subsites and stages

	Maintenance PPI users	
	Number	SIRs (95% CI)
Total colorectal adenocarcinomas	4,432	1.10 (1.06-1.13)
Sex		
Men	2,113	1.13 (1.08-1.18)
Women	2,319	1.10 (1.06-1.15)
Age at starting the maintenance PPI use, years		
18-39	38	2.79 (1.62-4.47)
40-49	145	2.02 (1.65-2.45)
50-59	531	1.27 (1.13-1.41)
60-69	1,277	1.26 (1.19-1.34)

For peer review only

	≥70	2,441	1.04 (1.00-1.07)
Calendar year period			
	2005-2006	3,215	1.29 (1.09-1.51)
	2007-2009	1,049	1.17 (1.12-1.22)
	2010-2012	168	1.07 (1.02-1.11)
Subsites of colorectal adenocarcinoma			
	Right-sided	1,884	1.26 (1.20-1.32)
	Left-sided	2,499	0.99 (0.96-1.03)
Stages of colorectal adenocarcinoma			
	Stage 0-I	674	1.22 (1.13-1.32)
	Stage II	1,102	1.10 (1.03-1.16)
	Stage III	1,017	1.07 (1.00-1.13)
	Stage IV	748	0.95 (0.88-1.02)
	Missing	891	1.19 (1.11-1.27)

For peer

2. Risk of colorectal adenocarcinoma

Compared to the general population, the overall risk of CRA was increased in maintenance PPI users (SIR 1.10, 95% CI 1.06-1.13). This risk was particularly affected by age, with the highest estimates present in those aged 18-39 years (SIR 2.79, 95% CI 1.62-4.47) and 40-49 years (SIR 2.02, 95% CI 1.65-2.45), while no convincing association was found in individuals ≥70 years old (SIR 1.04, 95% CI 1.00-1.07). In maintenance PPI users, the diagnosed CRA was particularly associated with an increased risk of early stage (Stage 0-I, SIR 1.12, 95% CI 1.04-1.21) (Table 2). In terms of tumour location, maintenance PPI users had a higher risk of right-sided CRA than the general population, regardless of age and sex (Tables 2&3). Meanwhile, no association between PPI use and CRA risk was found in left-sided CRC (Tables 2&3).

For peer review only - http://bmjopen.bmj.com/site/about/guidelines.xhtml

Table 3. Sex and age disparities in the risk of right-sided and left-sided colorectal adenocarcinomas in maintenance proton pump inhibitor (PPI) users expressed as standardised incidence ratios (SIRs) and 95% confidence intervals (CIs)

Cancer in categories	Right-sided colorectal adenocarcinoma		Left-sided colorectal adenocarcinoma	
	Number	SIR (95 % CI)	Number	SIR (95 % CI)
Sex				
Men	736	1.25 (1.16-1.34)	1,359	1.04 (0.98-1.10)
Women	1,148	1.27 (1.19-1.34)	1,14	0.94 (0.89-1.00)
Age at starting the maintenance PPI, years				
18-39	13	2.78 (1.02-6.06)	24	2.81 (1.40-5.03)
40-49	37	1.98 (1.31-2.89)	103	1.95 (1.53-2.46)
50-59	167	1.42 (1.14-1.75)	359	1.20 (1.05-1.37)
60-69	528	1.58 (1.43-1.75)	732	1.12 (1.03-1.21)
≥70	1,139	1.17 (1.11-1.24)	1,281	0.90 (0.86-0.95)

For peer review only

3. Common indications for maintenance PPI use

Indications for maintenance PPI use did not show the same trend for the drug-cancer-risk relationship in the exposed population. PPI use was not related to an increased risk of CRA among patients with Barrett's oesophagus. A marginally increased risk of CRA among PPI users was observed in participants with the indications of *Helicobacter pylori* infection or eradication (SIR 1.29, 95% CI 1.15-1.44), dyspepsia (SIR 1.29, 95% CI 1.16-1.42), gastro-duodenitis (SIR 1.28, 95% CI 1.19-1.38), gastroesophageal reflux (SIR 1.26, 95% CI 1.19-1.33), and peptic ulcer (SIR 1.16, 95% CI 1.07-1.26). Among individuals with the combined use of non-steroidal anti-inflammatory drugs or low-dose aspirin, maintenance PPI use was not significantly associated with the risk of CRA (Table 4**a**). Compared with the general population, most of the indications were related to an increased risk of right-sided CRAs in maintenance PPI users (Table 4**b**).

For peer review only

Table 4. Risk of (**a**) colorectal adenocarcinomas (**b**) right-sided and left-sided colorectal adenocarcinomas according to indications for maintenance proton pump inhibitor (PPI) users as expressed as standardised incidence ratios (SIRs) and 95% confidence intervals (CIs)

(**a**) risk of colorectal adenocarcinomas

Indications for maintenance of PPI use	Colorectal adenocarcinoma	
	Number of cases (‰ in maintenance PPI users)	SIR (95 % CI)
Without any below indication	879 (4.7)	1.13 (1.06-1.21)
Helicobacter pylori infection or eradication	328 (5.9)	1.29 (1.15-1.44)
Gastroesophageal reflux	1,352 (7)	1.26 (1.19-1.33)
Barrett's oesophagus	39 (6.8)	1.16 (0.83-1.59)
Peptic ulcer	625 (8.3)	1.16 (1.07-1.26)
Zollinger Ellison's syndrome	0	-
Gastro-duodenitis	739 (7.4)	1.28 (1.19-1.38)
Dyspepsia	368 (8.8)	1.29 (1.16-1.42)
Maintenance use of low-dose aspirin	2,026 (7.7)	1.00 (0.96-1.05)
Maintenance use of non-steroidal anti-inflammatory drugs	1,096 (4.7)	1.00 (0.94-1.06)
With any of the above indications	3,553 (6.3)	1.09 (1.05-1.12)

For peer review only

(**b**) risk of right-sided and left-sided colorectal adenocarcinomas

Indications for maintenance of PPI use	Right-sided colorectal adenocarcinoma		Left-sided colorectal adenocarcinoma	
	Number of cases (‰ in maintenance PPI users)	SIR (95 % CI)	Number of cases (‰ in maintenance PPI users)	SIR (95 % CI)
Without any below indication	360 (1.9)	1.27 (1.15-1.41)	511 (2.7)	1.05 (0.97-1.15)
Helicobacter pylori infection or eradication	148 (2.7)	1.63(1.37-1.91)	176 (3.2)	1.09 (0.94-1.27)
Gastroesophageal reflux	616 (3.2)	1.58 (1.45-1.70)	718 (3.7)	1.06 (0.99-1.15)
Barrett's oesophagus	17 (3)	1.53 (0.89-2.45)	21 (3.7)	0.95 (0.59-1.45)
Peptic ulcer	288 (3.8)	1.45 (1.28-1.62)	333 (4.4)	1.00 (0.90-1.11)
Zollinger Ellison's syndrome	0	-	0	-
Gastro-duodenitis	361 (3.6)	1.70 (1.53-1.88)	370 (3.7)	1.03 (0.93-1.14)
Dyspepsia	186 (4.4)	1.74 (1.50-2.01)	179 (4.3)	1.01 (0.87-1.17)
Maintenance use of low-dose aspirin	900 (3.4)	1.18 (1.10-1.26)	1,110 (4.2)	0.90 (0.85-0.95)
Maintenance use of non-steroidal anti-inflammatory drugs	397 (1.7)	0.98 (0.89-1.09)	685 (3)	1.00 (0.92-1.08)
With any of the above indications	1,524 (2.7)	1.26(1.19-1.32)	1,988 (3.5)	0.98 (0.94-1.02)

For peer review only

4. Duration of follow-up time

There was an elevated risk of CRA observed between the exposed population and the general population during the 1-2 years of follow-up (SIR 1.24, 95% CI 1.17-1.32). Thereafter, the results suggested slightly decreased risks of CRA in the 2-4 years (SIR 0.91, 95% CI 0.87-0.96), 4-6 years (SIR 0.65, 95% CI 0.61-0.68) and ≥6 years (SIR 0.85, 95% CI 0.77-0.92) of follow-up (Figure 1**a**). There was no obvious difference in CRA risks observed between the maintenance H_2RA users and the general population during the follow-up time (Figure 1**a**). An increased risk of right-sided CRA was also observed during the 1-2 years of follow-up (Figure 1**b**).

5. Comparison with maintenance H_2RA use

With a total of 19,795 individuals and 130,810 person-years of follow-up, the risk of CRA was not found to be associated with maintenance H_2RA use compared to the background population (N=115, SIR 0.85, 95% CI 0.71-1.03). The multivariable Poisson regression model of maintenance PPI versus H_2RA use showed an IRR of 1.05 (95% CI 0.87-1.27, P<0.05).

Discussion

In this large population-based study investigating the association between maintenance PPI use and CRC risk, we found a potential 10% higher risk of CRA compared with the general population. SIRs showed that maintenance PPI use might be associated with an increased risk of right-sided CRA compared with that in the general population. Young to middle-aged adult (i.e., 18-39 and 40-49 years old) participants showed a higher risk of CRA than the general population.

Our findings are in line with an estimated exposure effect of 2.03 (95% CI 1.56-2.63) between PPI users and CRC risk reported by a Taiwanese cohort(18), where immortal time bias could not be ruled out. In our study, we excluded 41,697 individuals with less than one year of follow-up to avoid detection bias. As CRA typically takes several to 20 years to develop from polyp to adenocarcinoma(45), cancer diagnoses or deaths within one year after new incident PPI prescriptions should be excluded because they could result in a biological effect indicating that PPI initiation preceded or increased the cancer risks. We have further carefully addressed the possibility that our strict exclusion of the first year of follow-up introduced immortal time bias. Therefore, we estimated the overall risk of CRA within the same study population starting follow-up from the first dispensed prescription date of PPIs. Supplementary Table S3 showed that the results were consistent with our main analysis, suggesting that immortal time bias might not be a problem; otherwise, the overestimates of healthy individuals should have led to an opposite observed effect.

In the duration analysis, maintenance PPI use was associated with an increased risk of CRA than the general population during the 1-2 years of follow-up. Following that,

the exposed population showed a slightly lower risk of CRA than the general population. Then, the exposed population had a lower but less decreased risk of CRA compared to the general population with ≥6 years of follow-up. A similar pattern was observed for the risk of right-sided CRA. This may be because of potential confounding introduced by ever users who received maintenance PPI use long before the study period, as we do not have information on prescribed drug use prior to the start of the study (July 2005) when the National Prescribed Drug Registry was not fully constructed. A longer follow-up (i.e., more than 10 years) and more extensive wash-out of ever users may be needed, so that an increasing risk of CRA would be observed. Meanwhile, reverse causality (i.e., the observed effect explained by reversely linking initiating PPI to subclinical symptoms of CRC) should also be considered during the follow-up. If CRC patients were prone to be prescribed maintenance-used PPIs, substantial positive effects on the exposure estimation might have influenced each stratum of the exposed population during our analysis. However, the different trends of right- vs left-sided CRC risk during different time periods (Figure 1**b**) and with different indications (Table 4**b**) indicated that the reverse causality due to the prevalent use of maintenance PPIs might be less concerning.

Among the indication subgroup analyses, only maintenance PPI users with the combined use of NSAIDs or low-dose aspirin showed a potential preventive effect against colorectal tumour progression. A previous American cohort study concluded that long-term use of NSAIDs and low-dose aspirin reduced the risk of CRC(46). Our study also provided evidence that long-term NSAID or low-dose aspirin use appeared to be beneficial in concomitant use with PPIs. While we acknowledged the existing literature of concomitant drug use of PPIs, the necessity for continued evaluation in light of the increased focus on combined PPI use with NSAIDs or low-dose aspirin still needs to be emphasized in assessing CRC preventive potential. Nevertheless, an increased risk of CRA was observed among the *Helicobacter pylori* infection or eradication, gastroesophageal reflux, gastro-duodenitis, and dyspepsia groups, which have not been established as the risk factors for CRA. The elevated risk could be explained by the fact that acid-related disorders facilitate changes in the upper-intestinal microbiome barrier owing to gastric acid over secretion(47,48). Gut dysbiosis and hypergastrinaemia may also contribute to the association between PPI use and colorectal carcinogenesis(15,49,50).

The strengths of our study include not only the high number of individuals and person-years but also the well-designed method for calculating cancer risks. The high-quality, high-coverage national registries allowed the identification of eligible individuals with a lengthy follow-up. Compared to previous studies, we constructed this cohort with many individuals and long follow-up times to minimise time-related bias. Moreover, the results are generalisable to other Nordic nations because comparable recording systems are maintained in other Nordic nations. The method enables us to compare large populations with different levels without considering migrations and drop-offs. In addition, we accounted for an alternative indicator of H_2RA to reduce the residual

confounding caused by PPI indications, which could lead to bidirectional results in the association between the exposure and outcome. H_2RAs are also used as acid suppressants to treat stomach acid production conditions like peptic ulcers, Zollinger-Ellison syndrome, and provide relief from excessive acid secretion symptoms(51). Similar to PPI, prolonged use of H_2RAs can lead to elevated systemic levels of gastrin, a hormone known to stimulate the proliferation of colorectal epithelium and contribute to colon adenoma progression(51,52). Yet, our study showed that H_2RAs may not be associated with CRA/CRC risks, in accordance with previous population-based studies(22,53). Thus, the residual confounding by indication was minimised when comparing maintenance PPI and H_2RA use.

Our study has certain limitations, including a lack of additional data on lifestyle risk factors such as smoking, alcohol, diet, and body mass index. Moreover, although we followed the participants up to a maximum duration of 7.5 years, a median follow-up time of 5.3 years may be insufficient. A longer median follow-up permits a more extended observation of all naturally evoked outcomes and a more extensive exclusion of prevalent use without harming the sample size and power too much. Nevertheless, during the long progression period, the clinical diagnosis of CRA can be delayed following the onset of cancerous symptoms, which will dilute the association between the PPI and CRC risk.

In our study, maintenance PPI users account for less than 11% of the Swedish population (7.1-7.6 million people)(54,55). The prevalence is lower than that of other Nordic countries, such as 15.5% in Denmark(56). But inappropriate PPI prescriptions are increasing among adults(57,58). Meanwhile, early onset CRC (diagnosed at <50 years of age) has been reported to be rising, especially as young populations at increased risk of CRC tend to have an unhealthy diet, a lack of exercise, obesity, and a low acceptance of colonoscopy(59–61). The message to clinics is that young to middle-aged people should pay attention to the potential risk of CRA associated with maintenance PPI use. Moreover, combining low-dose aspirin or other NSAIDs with maintenance PPI treatment could be meaningful to PPI users with common indications, although further studies are needed before any firm conclusions can be made. Finally, right-sided CRA is more clinically insidious, more advanced at diagnosis, and worse in prognosis than left-sided CRA(30,62,63). Early chemoprevention for maintenance PPI users should be considered to prevent poorer outcomes of right-sided CRA because of the significantly increased risk of right-sided CRA following maintenance PPI use observed in our study than the general population.

In conclusion, our study showed that maintenance PPI use may be associated with an increased risk of CRA. Nevertheless, we need more population-based studies with extended observation to confirm the association.

Acknowledgement

We wish to address our gratitude to the Swedish National Board of Health and Welfare (Socialstyrelsen) for collecting all the data and to all the participants who contributed to the data collection.

Declaration of interest

The authors declare no conflict of interest.

Funding

This study was sponsored by Svenska Läkaresallskapet (SLS-788731, SLS-788751, SLS-783091) and the Swedish Research Council (2020-01058). QL was supported by a China Scholarship Council Grant (201700260302). None of these sponsors had any role in the study design, the collection, the analysis, or the interpretation of data.

Author Contribution Statement

All authors designed the study and approved the final version of the manuscript (QL, XW, LE, OS, KF, NB). QL drafted the protocol, conducted the statistical analysis, and wrote the draft of the manuscript, which was revised and approved by all the other authors. NB was the guarantor of the study. QL and NB had full access to all the data and codes in the study.

Data availability statement

The datasets analysed during the current study are not publicly available due to the sharing agreement at Karolinska Institutet and the National Board of Health and Welfare but are available from the corresponding authors on reasonable request.

Ethics Approval Statement

No individual informed consent was required due to the registry-based nature of the data. Ethical approval was obtained from the Regional Ethical Review Board in Stockholm (2014/1291-31/4).

References:

1. Shin JM, Sachs G. Pharmacology of proton pump inhibitors. Curr Gastroenterol Rep [Internet]. 2008 [cited 2020 Oct 30];10(6):528–34. Available from: /pmc/articles/PMC2855237/?report=abstract
2. Yang YX, Hennessy S, Propert K, Hwang WT, Sedarat A, Lewis JD. Chronic Proton Pump Inhibitor Therapy and the Risk of Colorectal Cancer. Gastroenterology [Internet]. 2007 Sep 1 [cited 2019 May 2];133(3):748–54. Available from: http://www.gastrojournal.org/article/S0016508507011638/fulltext
3. Hálfdánarson ÓÖ, Pottegård A, Björnsson ES, Lund SH, Ogmundsdottir MH, Steingrímsson E, et al. Proton-pump inhibitors among adults: a nationwide drug-utilization study. Therap Adv Gastroenterol. 2018 Jan 1;11.
4. Lassalle M, Le Tri T, Bardou M, Biour M, Kirchgesner J, Rouby F, et al. Use of proton pump inhibitors in adults in France: a nationwide drug utilization study. Eur J Clin Pharmacol. 2020;76(3):449–57.
5. Freedberg DE, Kim LS, Yang YX. The Risks and Benefits of Long-term Use of Proton Pump Inhibitors: Expert Review and Best Practice Advice From the American Gastroenterological Association. Gastroenterology [Internet]. 2017 Mar 1 [cited 2022 Aug 31];152(4):706–15. Available from: https://pubmed.ncbi.nlm.nih.gov/28257716/
6. Katz PO, Gerson LB, Vela MF. Guidelines for the diagnosis and management of gastroesophageal reflux disease. American Journal of Gastroenterology [Internet]. 2013 [cited 2022 Sep 6];108(3):308–28. Available from: https://journals-lww-com.proxy.kib.ki.se/ajg/Fulltext/2013/03000/Guidelines_for_the_Diagnosis_and_Management_of.6.aspx
7. Targownik LE, Fisher DA, Saini SD. AGA Clinical Practice Update on De-Prescribing of Proton Pump Inhibitors: Expert Review. Gastroenterology [Internet]. 2022 Apr 1 [cited 2022 Sep 6];162(4):1334–42. Available from: https://pubmed.ncbi.nlm.nih.gov/35183361/
8. Kurlander JE, Gonzalez JJ, Saini SD. Deprescribing Proton Pump Inhibitors. JAMA Intern Med. 2020;180(12):1711–2.
9. Brusselaers N, Engstrand L, Lagergren J. Maintenance proton pump inhibition therapy and risk of oesophageal cancer. Cancer Epidemiol [Internet]. 2018 Apr 1 [cited 2018 Oct 18];53(November 2017):172–7. Available from: https://doi.org/10.1016/j.canep.2018.02.004
10. Schneider JL, Kolitsopoulos F, Corley DA. Risk of gastric cancer, gastrointestinal cancers and other cancers: A comparison of treatment with pantoprazole and other proton pump inhibitors. Aliment Pharmacol Ther. 2016 Jan 1;43(1):73–82.
11. Brusselaers N, Sadr-Azodi O, Engstrand L. Long-term proton pump inhibitor usage and the association with pancreatic cancer in Sweden. J Gastroenterol [Internet]. 2020 Apr 1 [cited 2021 Aug 31];55(4):453–61. Available from: https://pubmed.ncbi.nlm.nih.gov/31811561/
12. Imhann F, Bonder MJ, Vila AV, Fu J, Mujagic Z, Vork L, et al. Proton pump inhibitors affect the gut microbiome. Gut [Internet]. 2016 May [cited 2018 Oct 18];65(5):740–8. Available from: http://www.ncbi.nlm.nih.gov/pubmed/26657899
13. Vich Vila A, Collij V, Sanna S, Sinha T, Imhann F, Bourgonje AR, et al. Impact of commonly used drugs on the composition and metabolic function of the gut

For peer review only

microbiota. Nature Communications 2020 11:1 [Internet]. 2020 Jan 17 [cited 2022 Sep 29];11(1):1–11. Available from: https://www.nature.com/articles/s41467-019-14177-z
14. Dekkers KF, Sayols-Baixeras S, Baldanzi G, Nowak C, Hammar U, Nguyen D, et al. An online atlas of human plasma metabolite signatures of gut microbiome composition. Nature Communications 2022 13:1 [Internet]. 2022 Sep 23 [cited 2022 Sep 29];13(1):1–12. Available from: https://www.nature.com/articles/s41467-022-33050-0
15. Bruno G, Zaccari P, Rocco G, Scalese G, Panetta C, Porowska B, et al. Proton pump inhibitors and dysbiosis: Current knowledge and aspects to be clarified. World J Gastroenterol [Internet]. 2019 Jun 6 [cited 2022 Jun 21];25(22):2706. Available from: /pmc/articles/PMC6580352/
16. Chen CC, Liou JM, Lee YC, Hong TC, El-Omar EM, Wu MS. The interplay between Helicobacter pylori and gastrointestinal microbiota. Gut Microbes [Internet]. 2021 [cited 2022 Jun 21];13(1):1–22. Available from: /pmc/articles/PMC8096336/
17. Srivastava A, Gupta J, Kumar S, Kumar A. Gut biofilm forming bacteria in inflammatory bowel disease. Vol. 112, Microbial Pathogenesis. Academic Press; 2017. p. 5–14.
18. Lei WY, Wang JH, Yi CH, Liu TT, Hung JS, Wong MW, et al. Association between use of proton pump inhibitors and colorectal cancer: A nationwide population-based study. Clin Res Hepatol Gastroenterol. 2021 Jan 1;45(1):101397.
19. Lai SW, Liao KF, Lai HC, Lin CL, Sung FC. Use of proton pump inhibitors correlates with increased risk of colorectal cancer in Taiwan. Asia Pac J Clin Oncol. 2013 Jun 1;9(2):192–3.
20. Robertson DJ, Larsson H, Friis S, Pedersen L, Baron JA, Sørensen HT. Proton Pump Inhibitor Use and Risk of Colorectal Cancer: A Population-Based, Case-Control Study. Gastroenterology [Internet]. 2007 Sep 1 [cited 2019 May 2];133(3):755–60. Available from: http://www.gastrojournal.org/article/S0016508507011559/fulltext
21. Yang YX, Hennessy S, Propert K, Hwang WT, Sedarat A, Lewis JD. Chronic Proton Pump Inhibitor Therapy and the Risk of Colorectal Cancer. Gastroenterology. 2007 Sep 1;133(3):748–54.
22. Chubak J, Boudreau DM, Rulyak SJ, Mandelson MT. Colorectal cancer risk in relation to use of acid suppressive medications. Pharmacoepidemiol Drug Saf [Internet]. 2009 Jul 1 [cited 2022 Aug 25];18(7):540–4. Available from: https://onlinelibrary.wiley.com/doi/full/10.1002/pds.1749
23. Van Soest EM, Van Rossum LGM, Dieleman JP, Van Oijen MGH, Siersema PD, Sturkenboom MCJM, et al. Proton pump inhibitors and the risk of colorectal cancer. Am J Gastroenterol [Internet]. 2008 Apr [cited 2022 Aug 25];103(4):966–73. Available from: https://pubmed.ncbi.nlm.nih.gov/18070237/
24. Lee JK, Merchant SA, Schneider JL, Jensen CD, Fireman BH, Quesenberry CP, et al. Proton pump inhibitor use and risk of gastric, colorectal, liver, and pancreatic cancers in a community-based population. American Journal of Gastroenterology [Internet]. 2020 [cited 2022 Aug 26];115(5):706–15. Available from: https://journals.lww.com/ajg/Fulltext/2020/05000/Proton_Pump_Inhibitor_Use_and_Risk_of_Gastric,.18.aspx

For peer review only

25. Kuiper JG, Herk-Sukel MPP, Lemmens VEPP, Kuipers EJ, Herings RMC. Proton pump inhibitors are not associated with an increased risk of colorectal cancer. GastroHep. 2020 Jul;2(4):165–70.
26. Abrahami D, McDonald EG, Schnitzer ME, Barkun AN, Suissa S, Azoulay L. Proton pump inhibitors and risk of colorectal cancer. Gut. 2022;71(1):111–8.
27. Lai SW, Liao KF, Lai HC, Lin CL, Sung FC. Use of proton pump inhibitors correlates with increased risk of colorectal cancer in Taiwan. Asia Pac J Clin Oncol [Internet]. 2013 Jun 1 [cited 2022 Aug 26];9(2):192–3. Available from: https://onlinelibrary.wiley.com/doi/full/10.1111/ajco.12054
28. Hwang IC, Chang J, Park SM. Emerging hazard effects of proton pump inhibitor on the risk of colorectal cancer in low-risk populations: A Korean nationwide prospective cohort study. PLoS One. 2017 Dec 1;12(12):e0189114.
29. Thrumurthy SG, Thrumurthy SSD, Gilbert CE, Ross P, Haji A. Colorectal adenocarcinoma: risks, prevention and diagnosis. BMJ [Internet]. 2016 Jul 14 [cited 2022 Oct 4];354. Available from: https://www-bmj-com.proxy.kib.ki.se/content/354/bmj.i3590
30. Baran B, Mert Ozupek N, Yerli Tetik N, Acar E, Bekcioglu O, Baskin Y. Difference Between Left-Sided and Right-Sided Colorectal Cancer: A Focused Review of Literature. Gastroenterology Res [Internet]. 2018 [cited 2020 Oct 29];11(4):264–73. Available from: /pmc/articles/PMC6089587/?report=abstract
31. Strand DS, Kim D, Peura DA. 25 Years of Proton Pump Inhibitors: A Comprehensive Review. Gut Liver [Internet]. 2017 Jan 1 [cited 2022 Oct 24];11(1):27. Available from: /pmc/articles/PMC5221858/
32. Socialstyrelsen. The National Patient Register - Socialstyrelsen [Internet]. 2019 [cited 2020 Oct 29]. Available from: https://www.socialstyrelsen.se/en/statistics-and-data/registers/register-information/the-national-patient-register/
33. Swedish Cancer Registry Quality [Internet]. Available from: https://www.socialstyrelsen.se/en/statistics-and-data/registers/register-information/swedish-cancer-register/
34. Ludvigsson JF, Almqvist C, Bonamy AKE, Ljung R, Michaëlsson K, Neovius M, et al. Registers of the Swedish total population and their use in medical research. Eur J Epidemiol. 2016 Feb 1;31(2):125–36.
35. Brooke HL, Talbäck M, Hörnblad J, Johansson LA, Ludvigsson JF, Druid H, et al. The Swedish cause of death register. Eur J Epidemiol [Internet]. 2017 Sep 5 [cited 2020 Oct 29];32(9):765–73. Available from: http://link.springer.com/10.1007/s10654-017-0316-1
36. Wallerstedt SM, Wettermark B, Hoffmann M. The First Decade with the Swedish Prescribed Drug Register – A Systematic Review of the Output in the Scientific Literature. Basic Clin Pharmacol Toxicol [Internet]. 2016 Nov 24 [cited 2020 Oct 29];119(5):464–9. Available from: https://onlinelibrary.wiley.com/doi/10.1111/bcpt.12613
37. von Elm E, Altman DG, Egger M, Pocock SJ, Gøtzsche PC, Vandenbroucke JP. The Strengthening the Reporting of Observational Studies in Epidemiology (STROBE) statement: guidelines for reporting observational studies. The Lancet [Internet]. 2007 Oct 20 [cited 2022 Jun 28];370(9596):1453–7. Available from: http://www.thelancet.com/article/S014067360761602X/fulltext
38. FASS General - Home [Internet]. [cited 2021 Feb 2]. Available from: https://www.fass.se/LIF/startpage
39. Barlow L, Westergren K, Holmberg L, Talbäck M. The completeness of the Swedish Cancer Register – a sample survey for year 1998. Acta Oncol (Madr)

For peer review only

[Internet]. 2009 Jan 8 [cited 2020 Oct 30];48(1):27–33. Available from: http://www.tandfonline.com/doi/full/10.1080/02841860802247664
40. Vogel JD, Eskicioglu C, Weiser MR, Feingold DL, Steele SR. The American society of colon and rectal surgeons clinical practice guidelines for the treatment of colon cancer. Dis Colon Rectum. 2017;60(10):999–1017.
41. Brusselaers N, Lagergren J. Maintenance use of non-steroidal anti-inflammatory drugs and risk of gastrointestinal cancer in a nationwide population-based cohort study in Sweden. BMJ Open. 2018;8(7).
42. Machin D, Breslow NE, Day NE. Statistical Methods in Cancer Research, Vol. II: The Design and Analysis of Cohort Studies. Biometrics [Internet]. 1990 Dec;46(4):1243. Available from: https://www.jstor.org/stable/2532481?origin=crossref
43. Statistics database - National Board of Health and Welfare [Internet]. [cited 2024 Apr 2]. Available from: https://www.socialstyrelsen.se/statistik-och-data/statistik/statistikdatabasen/
44. Bender R. Introduction to the use of regression models in epidemiology. Methods in Molecular Biology. 2009;471:179–95.
45. Simon K. Colorectal cancer development and advances in screening. Clin Interv Aging. 2016;11:967–76.
46. Chan AT, Giovannucci EL, Meyerhardt JA, Schernhammer ES, Curhan GC, Fuchs CS. Long-term use of aspirin and nonsteroidal anti-inflammatory drugs and risk of colorectal cancer. JAMA [Internet]. 2005 Aug 1 [cited 2022 Sep 6];294(8):914–23. Available from: https://europepmc.org/articles/PMC1550973
47. Zhong L, Shanahan ER, Raj A, Koloski NA, Fletcher L, Morrison M, et al. Dyspepsia and the microbiome: time to focus on the small intestine. Vol. 66, Gut. England; 2017. p. 1168–9.
48. Minalyan A, Gabrielyan L, Scott D, Jacobs J, Pisegna JR. The Gastric and Intestinal Microbiome: Role of Proton Pump Inhibitors. Curr Gastroenterol Rep. 2017 Aug;19(8):42.
49. Macke L, Schulz C, Koletzko L, Malfertheiner P. Systematic review: the effects of proton pump inhibitors on the microbiome of the digestive tract-evidence from next-generation sequencing studies. Aliment Pharmacol Ther. 2020 Mar;51(5):505–26.
50. Robertson DJ, Sandler RS, Ahnen DJ, Greenberg ER, Mott LA, Cole BF, et al. Gastrin, Helicobacter pylori, and Colorectal Adenomas. Clinical Gastroenterology and Hepatology [Internet]. 2009 Feb [cited 2018 Dec 19];7(2):163–7. Available from: http://linkinghub.elsevier.com/retrieve/pii/S1542356508009415
51. Effects of three H2-receptor antagonists (cimetidine, famotidine, ranitidine) on serum gastrin level - PubMed [Internet]. [cited 2024 Apr 2]. Available from: https://pubmed.ncbi.nlm.nih.gov/12503773/
52. Baldwin GS, Shulkes A. Gastrin as an autocrine growth factor in colorectal carcinoma: implications for therapy. World J Gastroenterol [Internet]. 1998 Dec 12 [cited 2023 Sep 20];4(6):461. Available from: /pmc/articles/PMC4723428/
53. Babic A, Zhang X, Morales-Oyarvide V, Yuan C, Khalaf N, Khalili H, et al. Acid-suppressive medications and risk of colorectal cancer: results from three large prospective cohort studies. Br J Cancer [Internet]. 2020 Sep 9 [cited 2022 Aug 26];123(5):844–51. Available from: /pmc/articles/PMC7462971/

For peer review only

54. Population statistics [Internet]. [cited 2023 Nov 13]. Available from: https://www.scb.se/en/finding-statistics/statistics-by-subject-area/population/population-composition/population-statistics/
55. Brusselaers N, Wahlin K, Engstrand L, Lagergren J. Maintenance therapy with proton pump inhibitors and risk of gastric cancer: A nationwide population-based cohort study in Sweden. BMJ Open [Internet]. 2017 Oct 30 [cited 2019 Feb 7];7(10):e017739. Available from: http://www.ncbi.nlm.nih.gov/pubmed/29084798
56. Hálfdánarson Ó, Pottegård A, Björnsson ES, Lund SH, Ogmundsdottir MH, Steingrímsson E, et al. Proton-pump inhibitors among adults: a nationwide drug-utilization study. Therap Adv Gastroenterol. 2018;11:1–11.
57. Calvo LLJ, García Cámara P, Llorente Barrio M, Sierra Gabarda O, Monzón Baez R, Arbonés Mainar JM, et al. Successful deprescribing of proton pump inhibitors with a patient-centered process: the DESPIBP Project. Eur J Clin Pharmacol [Internet]. 2021;77(12):1927–33. Available from: https://doi.org/10.1007/s00228-021-03186-x
58. Muheim L, Signorell A, Markun S, Chmiel C, Neuner-Jehle S, Blozik E, et al. Potentially inappropriate proton-pump inhibitor prescription in the general population: a claims-based retrospective time trend analysis. Therap Adv Gastroenterol [Internet]. 2021 Jun;14(6):1756284821998928. Available from: http://www.ncbi.nlm.nih.gov/pubmed/28086035
59. Brenner DR, Ruan Y, Shaw E, De P, Heitman SJ, Hilsden RJ. Increasing colorectal cancer incidence trends among younger adults in Canada. Prev Med. 2017 Dec 1;105:345–9.
60. Vuik FER, Nieuwenburg SAV, Bardou M, Lansdorp-Vogelaar I, Dinis-Ribeiro M, Bento MJ, et al. Increasing incidence of colorectal cancer in young adults in Europe over the last 25 years. Gut. 2019;
61. Potter JD. Rising rates of colorectal cancer in younger adults. BMJ [Internet]. 2019 Jun 24 [cited 2022 Sep 15];365. Available from: https://www.bmj.com/content/365/bmj.l4280
62. Meguid RA, Slidell MB, Wolfgang CL, Chang DC, Ahuja N. Is there a difference in survival between right- versus left-sided colon cancers? Ann Surg Oncol [Internet]. 2008 Sep [cited 2020 Oct 29];15(9):2388–94. Available from: https://pubmed.ncbi.nlm.nih.gov/18622647/
63. Mik M, Berut M, Dziki L, Trzcinski R, Dziki A. Right-and left-sided colon cancer-clinical and pathological differences of the disease entity in one organ. Archives of Medical Science [Internet]. 2017 Feb 1 [cited 2020 Oct 29];13(1):157–62. Available from: /pmc/articles/PMC5206358/?report=abstract

For peer review only

Figure 1. Duration of follow-up and risk of colorectal adenocarcinomas among (**a**) maintenance proton pump inhibitor (PPI) users and maintenance histamine-2 receptor antagonist (H_2RA) users (**b**) overall maintenance PPI users and users diagnosed with right-sided or left-sided colorectal adenocarcinomas, expressed as standardised incidence ratios (SIRs) and 95% confidence intervals (CIs)

For peer review only

Figure 1. Duration of follow-up and risk of colorectal adenocarcinomas among (**a**) maintenance proton pump inhibitor (PPI) users and maintenance histamine-2 receptor antagonist (H_2RA) users (**b**) overall maintenance PPI users and users diagnosed with right-sided or left-sided colorectal adenocarcinomas, expressed as standardised incidence ratios (SIRs) and 95% confidence intervals (CIs)

(**a**) maintenance PPI users and H_2RA users

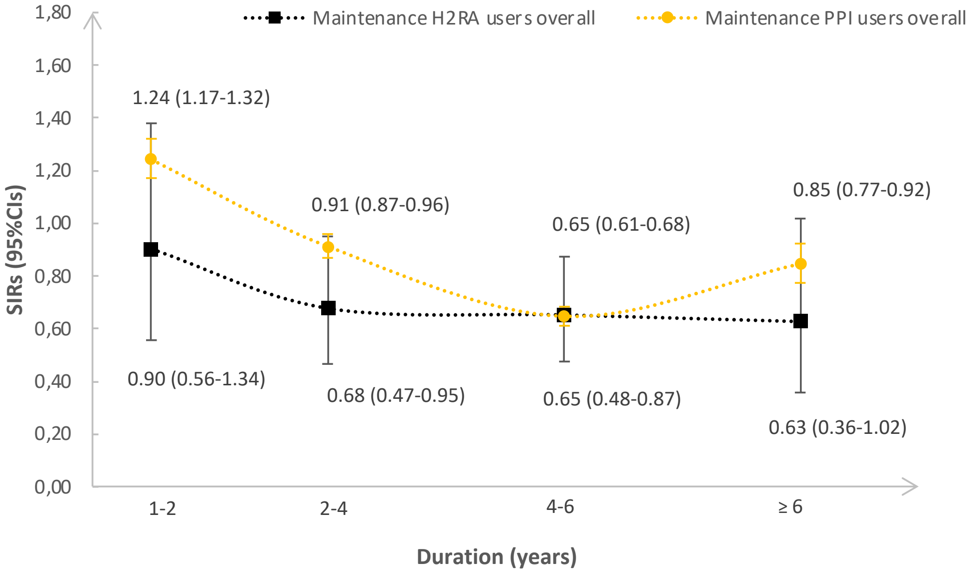

(**b**) overall maintenance PPI users and users diagnosed with right-sided or left-sided colorectal adenocarcinomas

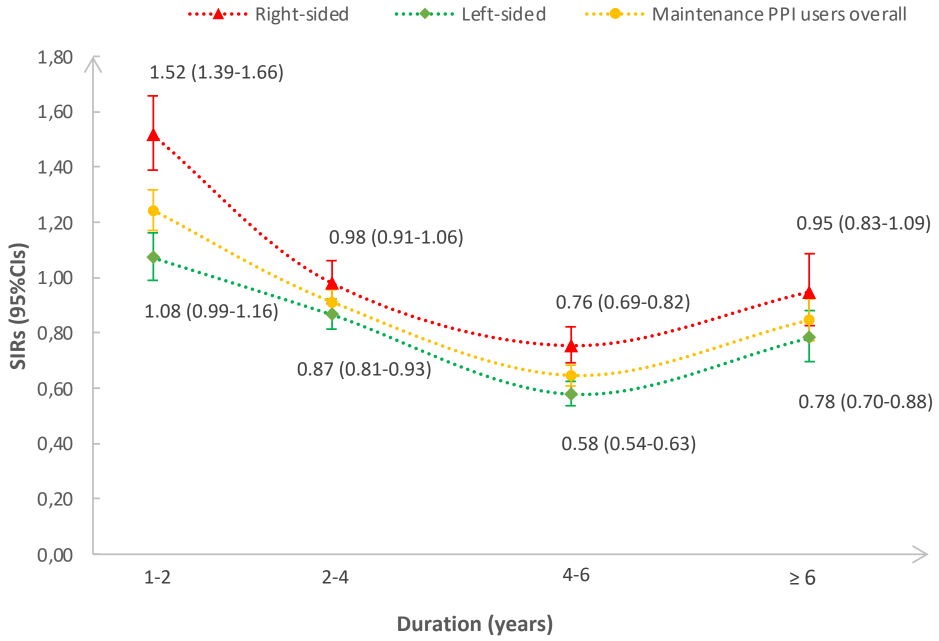

Supplementary Materials:

Table S1. International Classification of Diseases code for oncology and Anatomical Therapeutic Chemical codes used in this study

Table S2. Information of colorectal adenocarcinoma in the Swedish total population between 2005 and 2012

Table S3. Risk of colorectal adenocarcinomas in maintenance proton pump inhibitor (PPI) users followed up from enrolment, expressed as standardised incidence ratios (SIRs) and 95% confidence intervals (CIs) by age, sex, calendar period, tumour subsites and stages

Figure S1. Flow chart illustrating the enrolment of the study population of (**a**) maintenance proton pump inhibitor (PPI) users (**b**) maintenance histamine-2 receptor antagonist (H_2RA) users

For peer review only

Table S1. International Classification of Diseases code for oncology and Anatomical Therapeutic Chemical codes used in this study

Diseases	International Classification of Diseases code for oncology, 10th edition	Dispensed drugs	Anatomical Therapeutic Chemical code
Colorectal cancer	C18 -C20	Proton pump inhibitor	A02BC
Adenocarcinoma	096	Non-steroidal anti-inflammatory drug	M01A
Right-sided colorectal cancer	C18.0 -C18.4	Low-dose aspirin	B01AC06
Left-sided colorectal cancer	C18.5 - C18.7, C19, C20	Histamine-2 receptor antagonist	A02BA
Overlapping or unspecified sites of colorectal cancer	C18.8 C18.9		

For peer review only

Table S2. Information of colorectal adenocarcinoma in the Swedish total population between 2005 and 2012

Characteristics	Colorectal adenocarcinoma
Total	34,765
Age at entry the cohort, years	
18-39 years	532
40-49 years	1,324
50-59 years	3,974
60-69 years	9,298
≥70 years	19,637
Sex	
Men	18,601
Women	16,164
Calendar period	
2005-2006	7,998
2007-2009	13,044
2010-2012	13,723
Colorectal adenocarcinoma	
Right-sided	11,743
Left-sided	22,229
Overlapping or unspecified	793

For peer review only

Table S3. Risk of colorectal adenocarcinomas in maintenance proton pump inhibitor (PPI) users followed from enrolment, expressed as standardised incidence ratios (SIRs) and 95% confidence intervals (CIs) by age, sex, calendar period, tumour subsites and stages

	Maintenance PPI users with maximum follow-up time	
	Number	SIRs (95% CI)
Total colorectal adenocarcinomas	6,887	1.44 (1.40-1.47)
Sex		
Men	3,341	1.48 (1.43-1.53)
Women	3,546	1.40 (1.35-1.44)
Age at starting the maintenance PPI use, years		
18-39	67	5.53 (4.03-7.39)
40-49	218	2.75 (2.35-3.19)
50-59	775	1.78 (1.64-1.94)
60-69	1,870	1.60 (1.52-1.68)
≥70	3,957	1.32 (1.28-1.36)
Calendar year period		
2005-2006	4,126	1.76 (1.65-1.88)
2007-2009	1,982	1.55 (1.49-1.60)
2010-2012	779	1.28 (1.24-1.33)
Subsites of colorectal adenocarcinoma		
Right-sided	3,043	1.74 (1.68-1.81)
Left-sided	3,710	1.25 (1.21-1.29)
Stages of colorectal adenocarcinoma		
Stage 0-I	966	1.49 (1.40-1.59)
Stage II	1,657	1.41 (1.34-1.48)
Stage III	1,561	1.40 (1.33-1.47)
Stage IV	1,172	1.27 (1.19-1.34)
Missing	1,531	1.62 (1.54-1.70)
Follow-up time, years (median)	6.1	-

For peer review only

Figure S1. Flow chart illustrating the enrolment of the study population of (**a**) maintenance proton pump inhibitor (PPI) users (**b**) maintenance histamine-2 receptor antagonist (H_2RA) users

(**a**) maintenance PPI users

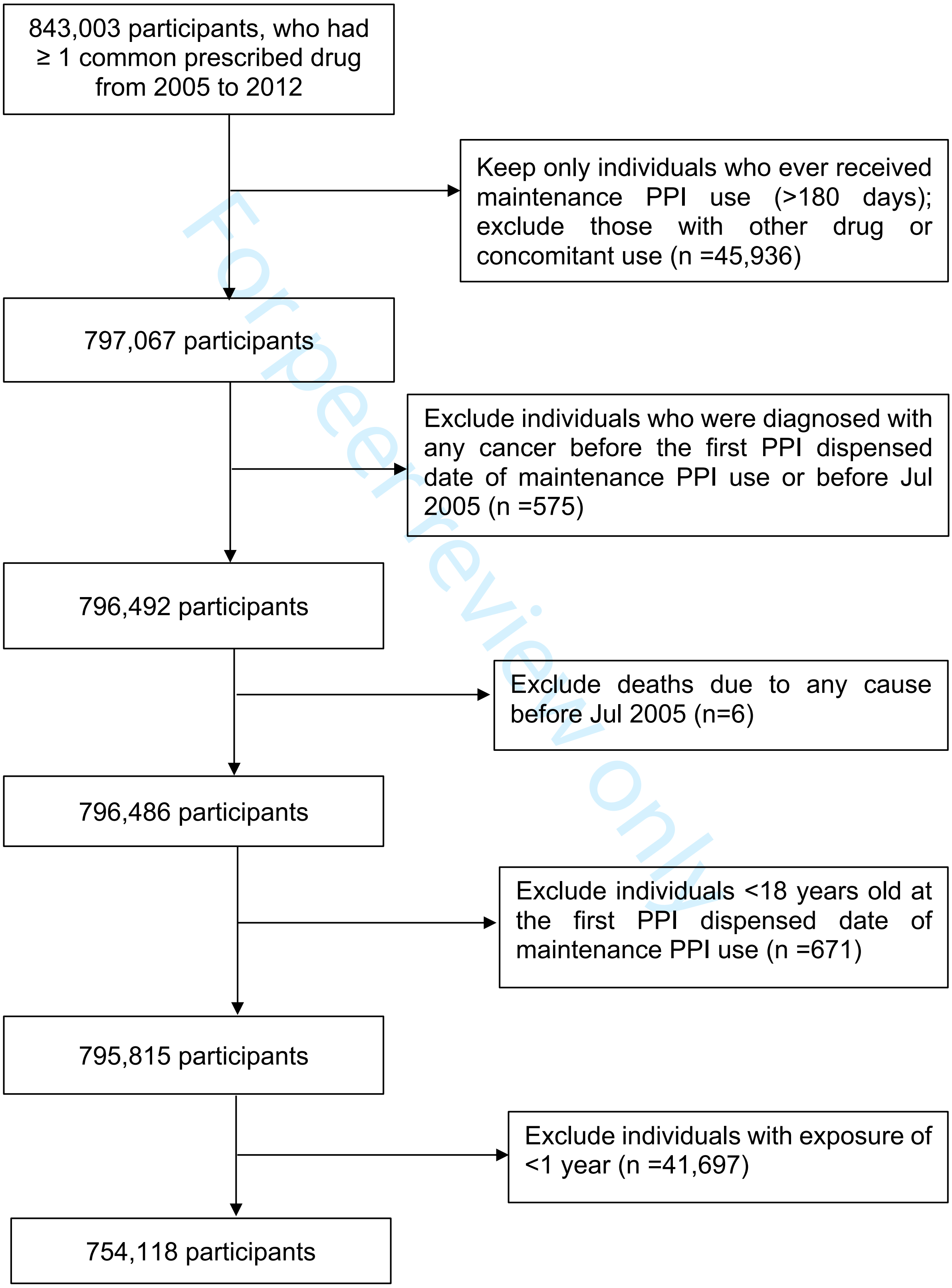

For peer review only

(**b**) maintenance H_2RA users

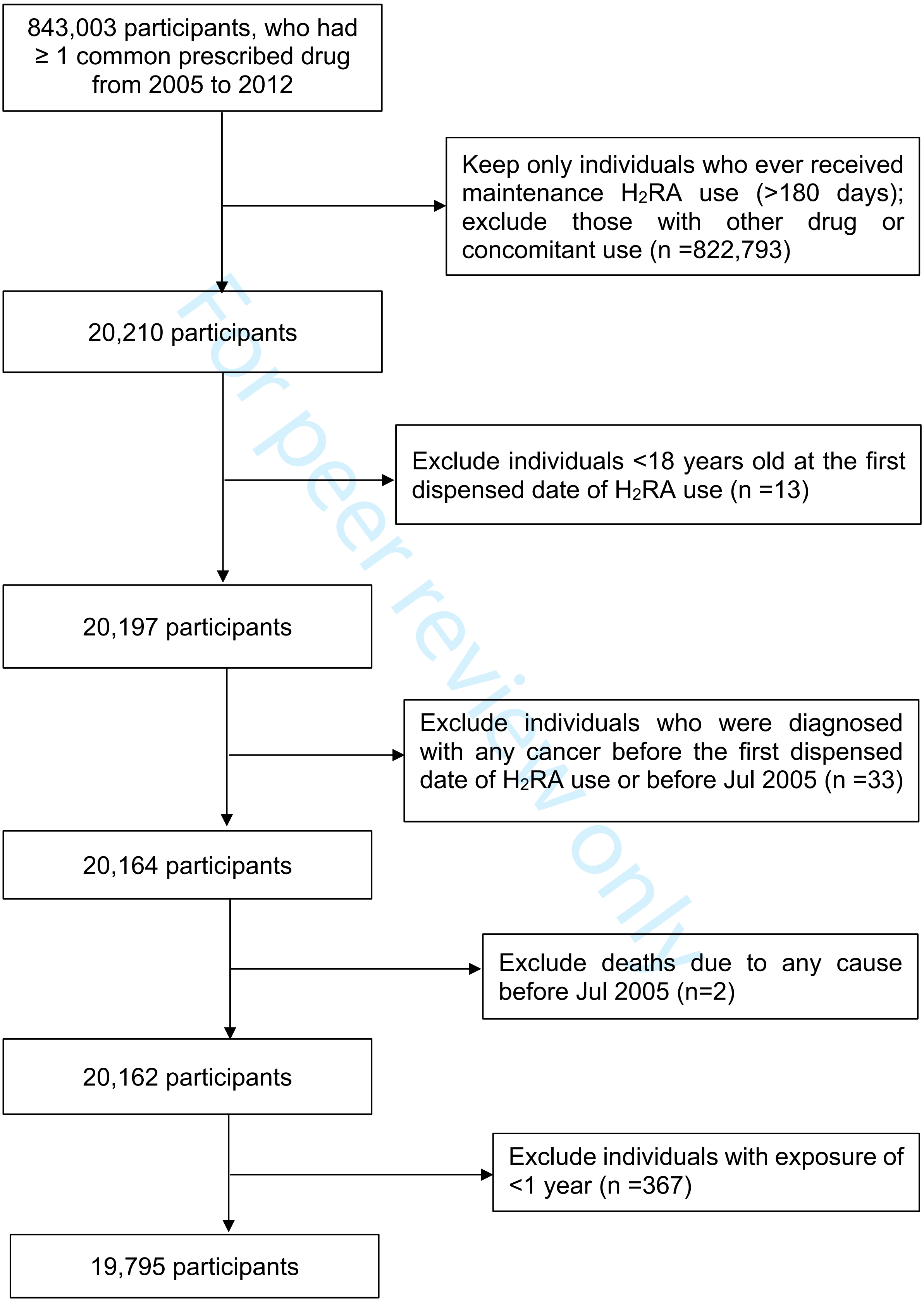

For peer review only

STROBE Statement—Checklist of items that should be included in reports of ***cohort studies***

	Item No	Recommendation	Page No
**Title and abstract**	1	(*a*) Indicate the study's design with a commonly used term in the title or the abstract<br>(*b*) Provide in the abstract an informative and balanced summary of what was done and what was found	1,2
**Introduction**			
Background/rationale	2	Explain the scientific background and rationale for the investigation being reported	4
Objectives	3	State specific objectives, including any prespecified hypotheses	4
**Methods**			
Study design	4	Present key elements of study design early in the paper	5
Setting	5	Describe the setting, locations, and relevant dates, including periods of recruitment, exposure, follow-up, and data collection	5
Participants	6	(*a*) Give the eligibility criteria, and the sources and methods of selection of participants. Describe methods of follow-up<br>(*b*) For matched studies, give matching criteria and number of exposed and unexposed	5
Variables	7	Clearly define all outcomes, exposures, predictors, potential confounders, and effect modifiers. Give diagnostic criteria, if applicable	6
Data sources/ measurement	8*	For each variable of interest, give sources of data and details of methods of assessment (measurement). Describe comparability of assessment methods if there is more than one group	5
Bias	9	Describe any efforts to address potential sources of bias	3,4,5
Study size	10	Explain how the study size was arrived at	5
Quantitative variables	11	Explain how quantitative variables were handled in the analyses. If applicable, describe which groupings were chosen and why	5,6
Statistical methods	12	(*a*) Describe all statistical methods, including those used to control for confounding<br>(*b*) Describe any methods used to examine subgroups and interactions<br>(*c*) Explain how missing data were addressed<br>(*d*) If applicable, explain how loss to follow-up was addressed<br>(*e*) Describe any sensitivity analyses	6,7
**Results**			
Participants	13*	(a) Report numbers of individuals at each stage of study—eg numbers potentially eligible, examined for eligibility, confirmed eligible, included in the study, completing follow-up, and analysed<br>(b) Give reasons for non-participation at each stage<br>(c) Consider use of a flow diagram	S1
Descriptive data	14*	(a) Give characteristics of study participants (eg demographic, clinical, social) and information on exposures and potential confounders<br>(b) Indicate number of participants with missing data for each variable of interest<br>(c) Summarise follow-up time (eg, average and total amount)	7-15
Outcome data	15*	Report numbers of outcome events or summary measures over time	7-15

For peer review only

For peer review only - http://bmjopen.bmj.com/site/about/guidelines.xhtml

Main results	16	(*a*) Give unadjusted estimates and, if applicable, confounder-adjusted estimates and their precision (eg, 95% confidence interval). Make clear which confounders were adjusted for and why they were included<br>(*b*) Report category boundaries when continuous variables were categorized<br>(*c*) If relevant, consider translating estimates of relative risk into absolute risk for a meaningful time period	7-15
Other analyses	17	Report other analyses done—eg analyses of subgroups and interactions, and sensitivity analyses	15
**Discussion**			
Key results	18	Summarise key results with reference to study objectives	15
Limitations	19	Discuss limitations of the study, taking into account sources of potential bias or imprecision. Discuss both direction and magnitude of any potential bias	17
Interpretation	20	Give a cautious overall interpretation of results considering objectives, limitations, multiplicity of analyses, results from similar studies, and other relevant evidence	15,16
Generalisability	21	Discuss the generalisability (external validity) of the study results	17
**Other information**			
Funding	22	Give the source of funding and the role of the funders for the present study and, if applicable, for the original study on which the present article is based	18

*Give information separately for exposed and unexposed groups.

Note: An Explanation and Elaboration article discusses each checklist item and gives methodological background and published examples of transparent reporting. The STROBE checklist is best used in conjunction with this article (freely available on the Web sites of PLoS Medicine at http://www.plosmedicine.org/, Annals of Internal Medicine at http://www.annals.org/, and Epidemiology at http://www.epidem.com/). Information on the STROBE Initiative is available at http://www.strobe-statement.org.

For peer review only

Maintenance proton pump inhibitor use and risk of colorectal cancer: A Swedish retrospective cohort study

Journal:	*BMJ Open*
Manuscript ID	bmjopen-2023-079591.R2
Article Type:	Original research
Date Submitted by the Author:	15-May-2024
Complete List of Authors:	Liu, Qing; Karolinska Institutet, Department of Microbiology, Tumour and Cell Biology<br>WANG, Xinchen; Karolinska Institutet, Department of Clinical Neuroscience<br>Engstrand, Lars; Karolinska Institutet, Microbiology, Tumour and Cell Biology<br>Sadr-Azodi, Omid; Karolinska Institutet<br>Fall, Katja; Karolinska Institutet, Dept of Medical Epidemiology and Biostatistic<br>Brusselaers, Nele; Karolinska Institutet, Molecular medicine and Surgery
**Primary Subject Heading**:	Epidemiology
Secondary Subject Heading:	Oncology, Pharmacology and therapeutics, Public health, Gastroenterology and hepatology
Keywords:	CLINICAL PHARMACOLOGY, EPIDEMIOLOGIC STUDIES, GASTROENTEROLOGY, Gastrointestinal tumours < GASTROENTEROLOGY, REGISTRIES, Risk Factors

SCHOLARONE™
Manuscripts

I, the Submitting Author has the right to grant and does grant on behalf of all authors of the Work (as defined in the below author licence), an exclusive licence and/or a non-exclusive licence for contributions from authors who are: i) UK Crown employees; ii) where BMJ has agreed a CC-BY licence shall apply, and/or iii) in accordance with the terms applicable for US Federal Government officers or employees acting as part of their official duties; on a worldwide, perpetual, irrevocable, royalty-free basis to BMJ Publishing Group Ltd ("BMJ") its licensees and where the relevant Journal is co-owned by BMJ to the co-owners of the Journal, to publish the Work in this journal and any other BMJ products and to exploit all rights, as set out in our licence.

The Submitting Author accepts and understands that any supply made under these terms is made by BMJ to the Submitting Author unless you are acting as an employee on behalf of your employer or a postgraduate student of an affiliated institution which is paying any applicable article publishing charge ("APC") for Open Access articles. Where the Submitting Author wishes to make the Work available on an Open Access basis (and intends to pay the relevant APC), the terms of reuse of such Open Access shall be governed by a Creative Commons licence – details of these licences and which Creative Commons licence will apply to this Work are set out in our licence referred to above.

Other than as permitted in any relevant BMJ Author's Self Archiving Policies, I confirm this Work has not been accepted for publication elsewhere, is not being considered for publication elsewhere and does not duplicate material already published. I confirm all authors consent to publication of this Work and authorise the granting of this licence.

For peer review only

Title: Maintenance proton pump inhibitor use and risk of colorectal cancer: A Swedish retrospective cohort study

Authors:

Qing LIU [1*], Xinchen WANG [2], Lars ENGSTRAND [1], Omid SADR-AZODI [3,4], Katja FALL[5,6], Nele BRUSSELAERS [1,7,8*]

Affiliations:

1 Centre for Translational Microbiome Research, Department of Microbiology, Tumour and Cell Biology, Karolinska Institutet, Stockholm, Sweden

2 Centre for Psychiatry Research, Department of Clinical Neuroscience, Karolinska Institutet, Stockholm, Sweden

3 Department of Clinical Sciences, Intervention and Technology, Karolinska Institutet, Stockholm, Sweden

4 Department of Surgery, Capio Saint Göran Hospital, Stockholm, Sweden

5 Clinical Epidemiology and Biostatistics School of Medical Sciences, Örebro University, Örebro, Sweden

6 Institute of Environmental Medicine, Karolinska Institutet, Stockholm, Sweden

7 Department of Head and Skin, Ghent University, Ghent, Belgium

8 Global Health Institute, Antwerp University, Antwerp, Belgium

Corresponding author: Qing Liu, qing.liu2017@outlook.com. Centre for Translational Microbiome Research, Department of Microbiology, Tumour and Cell biology, Karolinska Institutet. Biomedicum, kvarter 8a. Solnavägen 9, SE-171 65 Stockholm. Telephone: +46 (0) 8-524 845 97.

For peer review only

Abstract

Objectives: We aimed to evaluate the risk of colorectal adenocarcinoma (CRA) associated with long-term use of proton pump inhibitors (PPIs) in a large nationwide cohort.

Design: Retrospective cohort study.

Setting: This research was conducted at the national level, encompassing the entire population of Sweden.

Participants: This study utilized Swedish national registries to identify all adults who had ≥180 days of cumulative PPI use between July 2005 and December 2012, excluding participants who were followed up for less than one year. A total of 754,118 maintenance PPI users were included, with a maximum follow-up of 7.5 years.

Interventions: Maintenance PPI use (cumulative ≥180 days), with a comparator of maintenance histamine-2 receptor antagonist (H_2RA) use.

Primary and Secondary Outcome Measures: The primary outcome measure was the risk of CRA, presented as standardised incidence ratios (SIRs) with 95% confidence intervals (CIs). Subgroup analyses were performed to explore the impact of indications, tumour locations, tumour stages, and the duration of follow-up. A multivariable Poisson regression model was fitted to estimate the incidence rate ratios (IRRs) and 95% CIs of PPI versus H_2RA use.

Results: Maintenance PPI users exhibited a slightly elevated risk of CRA compared to the general population (SIR 1.10, 95% CI= 1.06-1.13) for both men and women. Individuals aged 18-39 (SIR 2.79, 95% CI=1.62-4.47) and 40-49 (SIR 2.02, 95% CI=1.65-2.45) had significantly higher risks than the general population. Right-sided CRA showed a higher risk compared to the general population (SIR 1.26, 95% CI=1.20-1.32). There was no significant difference in the risk of CRA between maintenance PPI users and maintenance H_2RA users (IRR 1.05, 95% CI=0.87-1.27, $P<0.05$).

Conclusions: Maintenance PPI use may be associated with an increased risk of CRA, but a prolonged observation time is needed.

Keywords: Cancer risk, Colorectal cancer, Proton pump inhibitors, Carcinogenesis, Histamine-2 receptor antagonist

Strengths and limitations of this study

- High number of enrolled individuals and person-years.
- High external validity and reduced residual confounding by the active comparator.
- Lack of additional data on some lifestyle risk factors.
- Potential confounding introduced by ever users who received maintenance PPI use long before the study period.
- Longer follow-up (i.e., more than 10 years) may be needed.

For peer review only

Introduction

Proton pump inhibitors (PPIs) are one of the most commonly prescribed drugs globally and are widely used for the treatment and prevention of various gastric acid-related disorders(1,2). The use of both prescription and over-the-counter PPIs has increased in many countries, with an estimated 10-30% of adults regularly using PPIs(3,4). Although PPIs have been deemed safe for short-term use and are recommended for a broad spectrum of indications by current guidelines and expert reviews(5–7), only a few indications justify long-term use exceeding 4 weeks. Despite this, PPIs are often overprescribed(7,8), which highlights the need for further investigation into the potential long-term side effects and public health implications of their widespread use.

Long-term PPI use has been associated with an increased risk of gastrointestinal cancers, including gastric, oesophageal and pancreatic cancers(9–11). Previous studies have demonstrated that PPI use significantly impacts the gut microbiome and contributes to dysbiosis(12–14), which has been linked to oncogenesis in the gut epithelium(15,16). Enteric infections and the trophic effects of PPI-induced hypergastrinaemia may also serve as potential cancerogenic factors(17). While some cohort studies have reported elevated relative risks of colorectal cancer (CRC) following PPI use(18,19), others have found no association between CRC and PPI use(20–26). However, potential methodological limitations in some of these studies have impacted the results. For example, some case-control studies introduced selection bias in exposure effect estimation by enrolling prevalent users rather than new users(22,23,25,27), and latency bias and time-window bias were present in some previous cohort studies due to the selection of narrow observation time ranges (19,22,28).

To clarify the relationship between PPI use and CRC, we conducted a large cohort study using high-coverage national registries to control for immortal time bias and time-window bias. We aimed to assess the association between maintenance PPI use and the risk of colorectal adenocarcinoma (CRA), which accounts for >95% of all CRC cases(29). We focused on right-sided and left-sided CRAs due to their chronological exposure to the drug-effects based on the anatomical locations(29,30). Additionally, we used the less popular histamine-2 receptor antagonists (H_2RAs) as active comparators in the supplementary analysis to minimise confounding-by-indication, as they share similar indications with PPIs and were used for treating gastric acid-related disorders before the introduction of PPIs as more potent acid suppressants(31).

Methods

Study design

This was a register-based cohort study that enrolled all Swedish adults (aged 18 years old) who were on maintenance PPI therapy between July 2005 and December 2012.

The study population was compared to the general population (i.e., the Swedish total population) stratified by the same age, sex, and calendar period. Participants were followed from one year after the first dispensed prescription date until the first diagnosis of CRA, death or the end of the study period (December 31, 2012). Data were obtained from the Swedish Cancer Registry, the National Patient Registry, the Cause of Death Registry, the Prescribed Drug Registry, and the Swedish Total Population Registry(32–36). Cancer and demographic information of the total population can be downloaded from the National Board of Health and Welfare's statistical database (32). The study protocol was established a priori, and the study was conducted in accordance with the Strengthening the Reporting of Observational Studies in Epidemiology (STROBE) statement(37).

Patient and public involvement

Due to its data-driven nature, this retrospective study had no patient or public directly involved in the study's design, data analysis, method selection or dissemination planning. Research questions and outcome measures were derived from existing literature and clinical relevance without direct input from them. Recruitment and follow-up relied on existing records from national registries.

Exposure

PPI usage data were obtained from the Prescribed Drug Registry, labelled by the (ATC)/defined daily dose (DDD) system. This system annotated the DDD per prescribed package with ATC codes. PPIs are also available over the counter in Sweden but only in small quantities and at exorbitant prices in comparison to prescribed PPIs(38). Therefore, most PPI consumption of long-term users is likely recorded in this registry. Maintenance PPI use (ATC code A02BC) was defined as cumulative use for at least 180 days prior to the onset of any cancer, death, or the end of the study period. Maintenance H_2RA (ATC code A02BA) use served as an active comparator, and it was defined as using H_2RA for at least 180 days before the onset of any cancer, death, or the end of the study period.

Outcome

The primary outcome was CRA, defined using the International Classification of Diseases (ICD, 10th edition) codes C18-C20 from the Swedish Cancer Registry(39), and the histopathology code 096. The tumours were further classified based on their anatomical location as right-sided, left-sided, or overlapping/unspecified sites (ICD codes in Supplementary Table S1). The tumour-node-metastasis (TNM) classification of the American Joint Committee on Cancer 8th edition Staging of Colorectal Cancer was used to define tumour stage and grouped as follows: stage 0-I (Tis, N0, M0 for stage 0; T1-2, N0, M0 for stage I), II (T3-4, N0, M0), III (T1-4, N1-2, M0), and IV (any T, any N, M1)(40).

Exclusions

Individuals with any documented cancer diagnosis prior to the study period or prior to the first dispensing date of PPIs were excluded. Individuals who switched between maintenance use of PPIs and H_2RAs (defined as ≥180 days of accumulated use of either drug) or had an exposure period of less than one year were also excluded. (Supplementary Figure S1)

Covariates

Age at treatment initiation was categorised into 18-39, 40-49, 50-59, 60-69 and ≥ 70 years old, and calendar periods were categorised into 2005-2006, 2007-2009, and 2010-2012. The duration of time since drug initiation was categorised as 1-2, 2-4, 4-6, and ≥ 6 years. No missing values for these variables were found for any of the study participants.

The most common recorded indications of maintenance PPI use were extracted from the National Patient Registry, including *Helicobacter pylori* infection or eradication, gastroesophageal reflux, Barrett's oesophagus, peptic ulcer, Zollinger Ellison's syndrome, gastro-duodenitis, dyspepsia, maintenance use (> 180 days during the study period) of low-dose aspirin, and maintenance use of NSAIDs(9,11,41) (ICD codes in Supplementary Table S1). We performed subgroup analyses on individuals with and without the listed indications, as well as on each indication separately when the statistical power was sufficient.

Statistical analyses

We calculated standardised incidence ratios (SIRs) with 95% confidence intervals (CIs) to assess the association between maintenance PPI use and CRA. Standardisation was performed according to Breslow and Day's method, and the allocation of person-years followed Clayton's algorithm (42). SIRs were obtained by calculating the ratio of observed CRA incidence rate compared to the expected incidence rate derived from the incidence among the Swedish total population of the same age, sex and calendar period. The information on the incidence of CRA (summarised in Supplementary Table S2) and the demographic characteristics of the total population can be downloaded from the National Board of Health and Welfare's statistical database (43). During the study period (2005-2012), the overall incidence of CRA in men ranged between 81.8-89.9/100,000 individuals and between 74.8-79.7/100,000 individuals in women (presented in Supplementary Figure 2). In our main analysis, we compared the overall risk of CRA in the study population to the general population of Sweden, stratifying the main outcomes by tumour stage (0-I, II, III, IV, and missing values), tumour location (right-sided, left-sided), indications, and follow-up time since drug initiation (1-2, 2-4, 4-6, and ≥ 6 years). We extensively investigated the risk of different tumour locations by age, sex, and indications for maintenance PPI use. Additionally, we assessed the

risk of CRA in maintenance PPI users with a maximal follow-up time from the first dispensed prescription date, considering age, sex, calendar period, tumour stage, and tumour location.

Furthermore, we conducted two supplementary analyses. First, we estimated SIRs for maintenance H_2RA users and conducted a duration analysis (1-2, 2-4, 4-6, and ≥ 6 years). Second, we used a multivariable Poisson regression model to estimate incidence rate ratios (IRRs) with 95% CIs to compare CRA risk between maintenance PPI users and H_2RA users, adjusting for age, sex and common therapy indications(44). The number of patients analysed for the Poisson regression model equals the summarized number of maintenance PPI users and maintenance H_2RA users. Propensity score adjustment between maintenance PPI users and H_2RA users was performed in the model to better control for confounders. Propensity scores were estimated using multivariable logistic regression, including age, sex, and indications. We applied *p* values less than 0.05 statistically significant for all the analyses. Stata MP 14.2 was used for all calculations.

Results

1. Characteristics of the PPI cohort

This study included 754,118 adult maintenance PPI users with no prior history of cancer, with a median follow-up of 5.3 years (totalling 4,177,396 person-years) and a maximum follow-up of 7.5 years. Women (0.52%) had a lower cumulative incidence of CRA than men (0.62%). Maintenance PPI use was most common in individuals aged 60-69 and ≥70 years old (Tables 1 & 2). A total of 4,432 cases of CRA were identified, with the majority being left-sided (N=2,499, 56.4%), stage II (N=1,102, 24.9%) and III (N=1,017, 22.9%).

Table 1. Characteristics of all maintenance proton pump inhibitor (PPI) users in Sweden between 2005 and 2012

Characteristics of the study population	Maintenance PPI users	
	Number	%
Total	754,118	100.0
Age at starting the maintenance PPI use, years		
18-39 years	84,510	11.2
40-49 years	99,897	13.3
50-59 years	149,167	19.8
60-69 years	167,456	22.2
≥70 years	253,088	33.6
Sex		
Men	310,357	41.2
Women	443,761	58.9

only

Calendar period		
2005-2006	430,527	57.1
2007-2009	221,369	29.4
2010-2012	102,222	13.6
Colorectal adenocarcinoma	4,432	0.6
Right-sided	1,884	0.3
Left-sided	2,499	0.3
Overlapping or unspecified	49	0.0
Tumour stages of colorectal adenocarcinoma		
Stage 0-I	674	0.1
Stage II	1,102	0.1
Stage III	1,017	0.1
Stage IV	748	0.1
Missing	891	0.1
Indications for maintenance PPI use		
Helicobacter pylori infection or eradication	55,311	7.3
Gastroesophageal reflux	193,371	25.6
Barrett's oesophagus	5,717	0.8
Peptic ulcer	74,946	10.0
Zollinger Ellison's syndrome	28	0.0
Gastro-duodenitis	100,191	13.2
Dyspepsia	41,916	5.6
Aspirin maintenance use	262,242	34.8
Non-steroidal anti-inflammatory drugs maintenance use	231,792	30.7
Without any of these recorded indications	186,529	24.7
Maintenance PPI use based on daily drug usage		
Interquartile range	1810	-
Person-years	3,426,925	-
Follow-up time, years (median)	5.3	-

Table 2. Risk of colorectal adenocarcinomas in maintenance proton pump inhibitor (PPI) users, expressed as cumulative incidence, standardised incidence ratios (SIRs) and 95% confidence

	Maintenance PPI users	
	Number	SIRs (95% CI)
Total colorectal adenocarcinomas	4,432	1.10 (1.06-1.13)
Sex		

For peer review only

Men	2,113	1.13 (1.08-1.18)
Women	2,319	1.10 (1.06-1.15)
Age at starting the maintenance PPI use, years		
18-39	38	2.79 (1.62-4.47)
40-49	145	2.02 (1.65-2.45)
50-59	531	1.27 (1.13-1.41)
60-69	1,277	1.26 (1.19-1.34)
≥70	2,441	1.04 (1.00-1.07)
Calendar year period		
2005-2006	3,215	1.29 (1.09-1.51)
2007-2009	1,049	1.17 (1.12-1.22)
2010-2012	168	1.07 (1.02-1.11)
Subsites of colorectal adenocarcinoma		
Right-sided	1,884	1.26 (1.20-1.32)
Left-sided	2,499	0.99 (0.96-1.03)
Stages of colorectal adenocarcinoma		
Stage 0-I	674	1.22 (1.13-1.32)
Stage II	1,102	1.10 (1.03-1.16)
Stage III	1,017	1.07 (1.00-1.13)
Stage IV	748	0.95 (0.88-1.02)
Missing	891	1.19 (1.11-1.27)

For peer review only

2. Risk of colorectal adenocarcinoma

Compared to the general population, the overall risk of CRA was increased in maintenance PPI users (SIR 1.10, 95% CI 1.06-1.13). This risk was particularly affected by age, with the highest estimates present in those aged 18-39 years (SIR 2.79, 95% CI 1.62-4.47) and 40-49 years (SIR 2.02, 95% CI 1.65-2.45), while no convincing association was found in individuals ≥70 years old (SIR 1.04, 95% CI 1.00-1.07). In maintenance PPI users, the diagnosed CRA was particularly associated with an increased risk of early stage (Stage 0-I, SIR 1.12, 95% CI 1.04-1.21) (Table 2). In terms of tumour location, maintenance PPI users had a higher risk of right-sided CRA than the general population, regardless of age and sex (Tables 2&3). Meanwhile, no association between PPI use and CRA risk was found in left-sided CRC (Tables 2&3).

For peer review only

Table 3. Sex and age disparities in the risk of right-sided and left-sided colorectal adenocarcinomas in maintenance proton pump inhibitor (PPI) users expressed as standardised incidence ratios (SIRs) and 95% confidence intervals (CIs)

Cancer in categories	Right-sided colorectal adenocarcinoma		Left-sided colorectal adenocarcinoma	
	Number	SIR (95 % CI)	Number	SIR (95 % CI)
Sex				
Men	736	1.25 (1.16-1.34)	1,359	1.04 (0.98-1.10)
Women	1,148	1.27 (1.19-1.34)	1,14	0.94 (0.89-1.00)
Age at starting the maintenance PPI, years				
18-39	13	2.78 (1.02-6.06)	24	2.81 (1.40-5.03)
40-49	37	1.98 (1.31-2.89)	103	1.95 (1.53-2.46)
50-59	167	1.42 (1.14-1.75)	359	1.20 (1.05-1.37)
60-69	528	1.58 (1.43-1.75)	732	1.12 (1.03-1.21)
≥70	1,139	1.17 (1.11-1.24)	1,281	0.90 (0.86-0.95)

For peer review only

3. Common indications for maintenance PPI use

Indications for maintenance PPI use did not show the same trend for the drug-cancer-risk relationship in the exposed population. PPI use was not related to an increased risk of CRA among patients with Barrett's oesophagus. A marginally increased risk of CRA among PPI users was observed in participants with the indications of *Helicobacter pylori* infection or eradication (SIR 1.29, 95% CI 1.15-1.44), dyspepsia (SIR 1.29, 95% CI 1.16-1.42), gastro-duodenitis (SIR 1.28, 95% CI 1.19-1.38), gastroesophageal reflux (SIR 1.26, 95% CI 1.19-1.33), and peptic ulcer (SIR 1.16, 95% CI 1.07-1.26). Among individuals with the combined use of non-steroidal anti-inflammatory drugs or low-dose aspirin, maintenance PPI use was not significantly associated with the risk of CRA (Table 4**a**). Compared with the general population, most of the indications were related to an increased risk of right-sided CRAs in maintenance PPI users (Table 4**b**).

peer review only

Table 4. Risk of (**a**) colorectal adenocarcinomas (**b**) right-sided and left-sided colorectal adenocarcinomas according to indications for maintenance proton pump inhibitor (PPI) users as expressed as standardised incidence ratios (SIRs) and 95% confidence intervals (CIs)

(**a**) risk of colorectal adenocarcinomas

Indications for maintenance of PPI use	Colorectal adenocarcinoma	
	Number of cases (‰ in maintenance PPI users)	SIR (95 % CI)
Without any below indication	879 (4.7)	1.13 (1.06-1.21)
Helicobacter pylori infection or eradication	328 (5.9)	1.29 (1.15-1.44)
Gastroesophageal reflux	1,352 (7)	1.26 (1.19-1.33)
Barrett's oesophagus	39 (6.8)	1.16 (0.83-1.59)
Peptic ulcer	625 (8.3)	1.16 (1.07-1.26)
Zollinger Ellison's syndrome	0	-
Gastro-duodenitis	739 (7.4)	1.28 (1.19-1.38)
Dyspepsia	368 (8.8)	1.29 (1.16-1.42)
Maintenance use of low-dose aspirin	2,026 (7.7)	1.00 (0.96-1.05)
Maintenance use of non-steroidal anti-inflammatory drugs	1,096 (4.7)	1.00 (0.94-1.06)
With any of the above indications	3,553 (6.3)	1.09 (1.05-1.12)

For peer review only

(**b**) risk of right-sided and left-sided colorectal adenocarcinomas

Indications for maintenance of PPI use	Right-sided colorectal adenocarcinoma		Left-sided colorectal adenocarcinoma	
	Number of cases (‰ in maintenance PPI users)	SIR (95 % CI)	Number of cases (‰ in maintenance PPI users)	SIR (95 % CI)
Without any below indication	360 (1.9)	1.27 (1.15-1.41)	511 (2.7)	1.05 (0.97-1.15)
Helicobacter pylori infection or eradication	148 (2.7)	1.63(1.37-1.91)	176 (3.2)	1.09 (0.94-1.27)
Gastroesophageal reflux	616 (3.2)	1.58 (1.45-1.70)	718 (3.7)	1.06 (0.99-1.15)
Barrett's oesophagus	17 (3)	1.53 (0.89-2.45)	21 (3.7)	0.95 (0.59-1.45)
Peptic ulcer	288 (3.8)	1.45 (1.28-1.62)	333 (4.4)	1.00 (0.90-1.11)
Zollinger Ellison's syndrome	0	-	0	-
Gastro-duodenitis	361 (3.6)	1.70 (1.53-1.88)	370 (3.7)	1.03 (0.93-1.14)
Dyspepsia	186 (4.4)	1.74 (1.50-2.01)	179 (4.3)	1.01 (0.87-1.17)
Maintenance use of low-dose aspirin	900 (3.4)	1.18 (1.10-1.26)	1,110 (4.2)	0.90 (0.85-0.95)
Maintenance use of non-steroidal anti-inflammatory drugs	397 (1.7)	0.98 (0.89-1.09)	685 (3)	1.00 (0.92-1.08)
With any of the above indications	1,524 (2.7)	1.26(1.19-1.32)	1,988 (3.5)	0.98 (0.94-1.02)

For peer review only

4. Duration of follow-up time

There was an elevated risk of CRA observed between the exposed population and the general population during the 1-2 years of follow-up (SIR 1.24, 95% CI 1.17-1.32). Thereafter, the results suggested slightly decreased risks of CRA in the 2-4 years (SIR 0.91, 95% CI 0.87-0.96), 4-6 years (SIR 0.65, 95% CI 0.61-0.68) and ≥6 years (SIR 0.85, 95% CI 0.77-0.92) of follow-up (Figure 1**a**). There was no obvious difference in CRA risks observed between the maintenance H_2RA users and the general population during the follow-up time (Figure 1**a**). An increased risk of right-sided CRA was also observed during the 1-2 years of follow-up (Figure 1**b**).

5. Comparison with maintenance H_2RA use

With a total of 19,795 individuals and 130,810 person-years of follow-up, the risk of CRA was not found to be associated with maintenance H_2RA use compared to the background population (N=115, SIR 0.85, 95% CI 0.71-1.03). The multivariable Poisson regression model of maintenance PPI versus H_2RA use showed an IRR of 1.05 (95% CI 0.87-1.27, $P<0.05$).

Discussion

In this large population-based study investigating the association between maintenance PPI use and CRC risk, we found a potential 10% higher risk of CRA compared with the general population. SIRs showed that maintenance PPI use might be associated with an increased risk of right-sided CRA compared with that in the general population. Young to middle-aged adult (i.e., 18-39 and 40-49 years old) participants showed a higher risk of CRA than the general population.

Our findings are in line with an estimated exposure effect of 2.03 (95% CI 1.56-2.63) between PPI users and CRC risk reported by a Taiwanese cohort(18), where immortal time bias could not be ruled out. In our study, we excluded 41,697 individuals with less than one year of follow-up to avoid detection bias. As CRA typically takes several to 20 years to develop from polyp to adenocarcinoma(45), cancer diagnoses or deaths within one year after new incident PPI prescriptions should be excluded because they could result in a biological effect indicating that PPI initiation preceded or increased the cancer risks. We have further carefully addressed the possibility that our strict exclusion of the first year of follow-up introduced immortal time bias. Therefore, we estimated the overall risk of CRA within the same study population starting follow-up from the first dispensed prescription date of PPIs. Supplementary Table S3 showed that the results were consistent with our main analysis, suggesting that immortal time bias might not be a problem; otherwise, the overestimates of healthy individuals should have led to an opposite observed effect.

In the duration analysis, maintenance PPI use was associated with an increased risk of CRA than the general population during the 1-2 years of follow-up. Following that, the exposed population showed a slightly lower risk of CRA than the general population. Then, the exposed population had a lower but less decreased risk of CRA compared to the general population with ≥6 years of follow-up. A similar pattern was observed for the risk of right-sided CRA. This may be because of potential confounding introduced by ever users who received maintenance PPI use long before the study period, as we do not have information on prescribed drug use prior to the start of the study (July 2005) when the National Prescribed Drug Registry was not fully constructed. A longer follow-up (i.e., more than 10 years) and more extensive wash-out of ever users may be needed, so that an increasing risk of CRA would be observed. Meanwhile, reverse causality (i.e., the observed effect explained by reversely linking initiating PPI to subclinical symptoms of CRC) should also be considered during the follow-up. If CRC patients were prone to be prescribed maintenance-used PPIs, substantial positive effects on the exposure estimation might have influenced each stratum of the exposed population during our analysis. However, the different trends of right- vs left-sided CRC risk during different time periods (Figure 1**b**) and with different indications (Table 4**b**) indicated that the reverse causality due to the prevalent use of maintenance PPIs might be less concerning.

Among the indication subgroup analyses, only maintenance PPI users with the combined use of NSAIDs or low-dose aspirin showed a potential preventive effect against colorectal tumour progression. A previous American cohort study concluded that long-term use of NSAIDs and low-dose aspirin reduced the risk of CRC(46). Our study also provided evidence that long-term NSAID or low-dose aspirin use appeared to be beneficial in concomitant use with PPIs. While we acknowledged the existing literature of concomitant drug use of PPIs, the necessity for continued evaluation in light of the increased focus on combined PPI use with NSAIDs or low-dose aspirin still needs to be emphasized in assessing CRC preventive potential. Nevertheless, an increased risk of CRA was observed among the *Helicobacter pylori* infection or eradication, gastroesophageal reflux, gastro-duodenitis, and dyspepsia groups, which have not been established as the risk factors for CRA. The elevated risk could be explained by the fact that acid-related disorders facilitate changes in the upper-intestinal microbiome barrier owing to gastric acid over secretion(47,48). Gut dysbiosis and hypergastrinaemia may also contribute to the association between PPI use and colorectal carcinogenesis(15,49,50).

The strengths of our study include not only the high number of individuals and person-years but also the well-designed method for calculating cancer risks. The high-quality, high-coverage national registries allowed the identification of eligible individuals with a lengthy follow-up. Compared to previous studies, we constructed this cohort with many individuals and long follow-up times to minimise time-related bias. Moreover, the results are generalisable to other Nordic nations because comparable recording systems are maintained in other Nordic nations. The method enables us to compare

large populations with different levels without considering migrations and drop-offs. In addition, we accounted for an alternative indicator of H_2RA to reduce the residual confounding caused by PPI indications, which could lead to bidirectional results in the association between the exposure and outcome. H_2RAs are also used as acid suppressants to treat stomach acid production conditions like peptic ulcers, Zollinger-Ellison syndrome, and provide relief from excessive acid secretion symptoms(51). Similar to PPI, prolonged use of H_2RAs can lead to elevated systemic levels of gastrin, a hormone known to stimulate the proliferation of colorectal epithelium and contribute to colon adenoma progression(51,52). Yet, our study showed that H_2RAs may not be associated with CRA/CRC risks, in accordance with previous population-based studies(22,53). Thus, the residual confounding by indication was minimised when comparing maintenance PPI and H_2RA use.

Our study has certain limitations, including a lack of additional data on lifestyle risk factors such as smoking, alcohol, diet, and body mass index. Moreover, although we followed the participants up to a maximum duration of 7.5 years, a median follow-up time of 5.3 years may be insufficient. A longer median follow-up permits a more extended observation of all naturally evoked outcomes and a more extensive exclusion of prevalent use without harming the sample size and power too much. Nevertheless, during the long progression period, the clinical diagnosis of CRA can be delayed following the onset of cancerous symptoms, which will dilute the association between the PPI and CRC risk.

In our study, maintenance PPI users account for less than 11% of the Swedish population (7.1-7.6 million people)(54,55). The prevalence is lower than that of other Nordic countries, such as 15.5% in Denmark(56). But inappropriate PPI prescriptions are increasing among adults(57,58). Meanwhile, early onset CRC (diagnosed at <50 years of age) has been reported to be rising, especially as young populations at increased risk of CRC tend to have an unhealthy diet, a lack of exercise, obesity, and a low acceptance of colonoscopy(59–61). The message to clinics is that young to middle-aged people should pay attention to the potential risk of CRA associated with maintenance PPI use. Moreover, combining low-dose aspirin or other NSAIDs with maintenance PPI treatment could be meaningful to PPI users with common indications, although further studies are needed before any firm conclusions can be made. Finally, right-sided CRA is more clinically insidious, more advanced at diagnosis, and worse in prognosis than left-sided CRA(30,62,63). Early chemoprevention for maintenance PPI users should be considered to prevent poorer outcomes of right-sided CRA because of the significantly increased risk of right-sided CRA following maintenance PPI use observed in our study than the general population.

In conclusion, our study showed that maintenance PPI use may be associated with an increased risk of CRA. Nevertheless, we need more population-based studies with extended observation to confirm the association.

For peer review only - http://bmjopen.bmj.com/site/about/guidelines.xhtml

Acknowledgement

We wish to address our gratitude to the Swedish National Board of Health and Welfare (Socialstyrelsen) for collecting all the data and to all the participants who contributed to the data collection.

Declaration of interest

The authors declare no conflict of interest.

Funding

This study was sponsored by Svenska Läkaresallskapet (SLS-788731, SLS-788751, SLS-783091) and the Swedish Research Council (2020-01058). QL was supported by a China Scholarship Council Grant (201700260302). None of these sponsors had any role in the study design, the collection, the analysis, or the interpretation of data.

Author Contribution Statement

All authors designed the study and approved the final version of the manuscript (QL, XW, LE, OS, KF, NB). QL drafted the protocol, conducted the statistical analysis, and wrote the draft of the manuscript, which was revised and approved by all the other authors. NB was the guarantor of the study. QL and NB had full access to all the data and codes in the study.

Data availability statement

The datasets analysed during the current study are not publicly available due to the sharing agreement at Karolinska Institutet and the National Board of Health and Welfare but are available from the corresponding authors on reasonable request.

Ethics Approval Statement

No individual informed consent was required due to the registry-based nature of the data. Ethical approval was obtained from the Regional Ethical Review Board in Stockholm (2014/1291-31/4).

References:

1. Shin JM, Sachs G. Pharmacology of proton pump inhibitors. Curr Gastroenterol Rep [Internet]. 2008 [cited 2020 Oct 30];10(6):528–34. Available from: /pmc/articles/PMC2855237/?report=abstract
2. Yang YX, Hennessy S, Propert K, Hwang WT, Sedarat A, Lewis JD. Chronic Proton Pump Inhibitor Therapy and the Risk of Colorectal Cancer. Gastroenterology [Internet]. 2007 Sep 1 [cited 2019 May 2];133(3):748–54. Available from: http://www.gastrojournal.org/article/S0016508507011638/fulltext
3. Hálfdánarson ÓÖ, Pottegård A, Björnsson ES, Lund SH, Ogmundsdottir MH, Steingrímsson E, et al. Proton-pump inhibitors among adults: a nationwide drug-utilization study. Therap Adv Gastroenterol. 2018 Jan 1;11.
4. Lassalle M, Le Tri T, Bardou M, Biour M, Kirchgesner J, Rouby F, et al. Use of proton pump inhibitors in adults in France: a nationwide drug utilization study. Eur J Clin Pharmacol. 2020;76(3):449–57.
5. Freedberg DE, Kim LS, Yang YX. The Risks and Benefits of Long-term Use of Proton Pump Inhibitors: Expert Review and Best Practice Advice From the American Gastroenterological Association. Gastroenterology [Internet]. 2017 Mar 1 [cited 2022 Aug 31];152(4):706–15. Available from: https://pubmed.ncbi.nlm.nih.gov/28257716/
6. Katz PO, Gerson LB, Vela MF. Guidelines for the diagnosis and management of gastroesophageal reflux disease. American Journal of Gastroenterology [Internet]. 2013 [cited 2022 Sep 6];108(3):308–28. Available from: https://journals-lww-com.proxy.kib.ki.se/ajg/Fulltext/2013/03000/Guidelines_for_the_Diagnosis_and_Management_of.6.aspx
7. Targownik LE, Fisher DA, Saini SD. AGA Clinical Practice Update on De-Prescribing of Proton Pump Inhibitors: Expert Review. Gastroenterology [Internet]. 2022 Apr 1 [cited 2022 Sep 6];162(4):1334–42. Available from: https://pubmed.ncbi.nlm.nih.gov/35183361/
8. Kurlander JE, Gonzalez JJ, Saini SD. Deprescribing Proton Pump Inhibitors. JAMA Intern Med. 2020;180(12):1711–2.
9. Brusselaers N, Engstrand L, Lagergren J. Maintenance proton pump inhibition therapy and risk of oesophageal cancer. Cancer Epidemiol [Internet]. 2018 Apr 1 [cited 2018 Oct 18];53(November 2017):172–7. Available from: https://doi.org/10.1016/j.canep.2018.02.004
10. Schneider JL, Kolitsopoulos F, Corley DA. Risk of gastric cancer, gastrointestinal cancers and other cancers: A comparison of treatment with pantoprazole and other proton pump inhibitors. Aliment Pharmacol Ther. 2016 Jan 1;43(1):73–82.
11. Brusselaers N, Sadr-Azodi O, Engstrand L. Long-term proton pump inhibitor usage and the association with pancreatic cancer in Sweden. J Gastroenterol [Internet]. 2020 Apr 1 [cited 2021 Aug 31];55(4):453–61. Available from: https://pubmed.ncbi.nlm.nih.gov/31811561/
12. Imhann F, Bonder MJ, Vila AV, Fu J, Mujagic Z, Vork L, et al. Proton pump inhibitors affect the gut microbiome. Gut [Internet]. 2016 May [cited 2018 Oct 18];65(5):740–8. Available from: http://www.ncbi.nlm.nih.gov/pubmed/26657899
13. Vich Vila A, Collij V, Sanna S, Sinha T, Imhann F, Bourgonje AR, et al. Impact of commonly used drugs on the composition and metabolic function of the gut microbiota. Nature Communications 2020 11:1 [Internet]. 2020 Jan 17 [cited 2022 Sep 29];11(1):1–11. Available from: https://www.nature.com/articles/s41467-019-14177-z
14. Dekkers KF, Sayols-Baixeras S, Baldanzi G, Nowak C, Hammar U, Nguyen D, et al. An online atlas of human plasma metabolite signatures of gut microbiome

For peer review only

composition. Nature Communications 2022 13:1 [Internet]. 2022 Sep 23 [cited 2022 Sep 29];13(1):1–12. Available from: https://www.nature.com/articles/s41467-022-33050-0
15. Bruno G, Zaccari P, Rocco G, Scalese G, Panetta C, Porowska B, et al. Proton pump inhibitors and dysbiosis: Current knowledge and aspects to be clarified. World J Gastroenterol [Internet]. 2019 Jun 6 [cited 2022 Jun 21];25(22):2706. Available from: /pmc/articles/PMC6580352/
16. Chen CC, Liou JM, Lee YC, Hong TC, El-Omar EM, Wu MS. The interplay between Helicobacter pylori and gastrointestinal microbiota. Gut Microbes [Internet]. 2021 [cited 2022 Jun 21];13(1):1–22. Available from: /pmc/articles/PMC8096336/
17. Srivastava A, Gupta J, Kumar S, Kumar A. Gut biofilm forming bacteria in inflammatory bowel disease. Vol. 112, Microbial Pathogenesis. Academic Press; 2017. p. 5–14.
18. Lei WY, Wang JH, Yi CH, Liu TT, Hung JS, Wong MW, et al. Association between use of proton pump inhibitors and colorectal cancer: A nationwide population-based study. Clin Res Hepatol Gastroenterol. 2021 Jan 1;45(1):101397.
19. Lai SW, Liao KF, Lai HC, Lin CL, Sung FC. Use of proton pump inhibitors correlates with increased risk of colorectal cancer in Taiwan. Asia Pac J Clin Oncol. 2013 Jun 1;9(2):192–3.
20. Robertson DJ, Larsson H, Friis S, Pedersen L, Baron JA, Sørensen HT. Proton Pump Inhibitor Use and Risk of Colorectal Cancer: A Population-Based, Case-Control Study. Gastroenterology [Internet]. 2007 Sep 1 [cited 2019 May 2];133(3):755–60. Available from: http://www.gastrojournal.org/article/S0016508507011559/fulltext
21. Yang YX, Hennessy S, Propert K, Hwang WT, Sedarat A, Lewis JD. Chronic Proton Pump Inhibitor Therapy and the Risk of Colorectal Cancer. Gastroenterology. 2007 Sep 1;133(3):748–54.
22. Chubak J, Boudreau DM, Rulyak SJ, Mandelson MT. Colorectal cancer risk in relation to use of acid suppressive medications. Pharmacoepidemiol Drug Saf [Internet]. 2009 Jul 1 [cited 2022 Aug 25];18(7):540–4. Available from: https://onlinelibrary.wiley.com/doi/full/10.1002/pds.1749
23. Van Soest EM, Van Rossum LGM, Dieleman JP, Van Oijen MGH, Siersema PD, Sturkenboom MCJM, et al. Proton pump inhibitors and the risk of colorectal cancer. Am J Gastroenterol [Internet]. 2008 Apr [cited 2022 Aug 25];103(4):966–73. Available from: https://pubmed.ncbi.nlm.nih.gov/18070237/
24. Lee JK, Merchant SA, Schneider JL, Jensen CD, Fireman BH, Quesenberry CP, et al. Proton pump inhibitor use and risk of gastric, colorectal, liver, and pancreatic cancers in a community-based population. American Journal of Gastroenterology [Internet]. 2020 [cited 2022 Aug 26];115(5):706–15. Available from: https://journals.lww.com/ajg/Fulltext/2020/05000/Proton_Pump_Inhibitor_Use_and_Risk_of_Gastric,.18.aspx
25. Kuiper JG, Herk-Sukel MPP, Lemmens VEPP, Kuipers EJ, Herings RMC. Proton pump inhibitors are not associated with an increased risk of colorectal cancer. GastroHep. 2020 Jul;2(4):165–70.
26. Abrahami D, McDonald EG, Schnitzer ME, Barkun AN, Suissa S, Azoulay L. Proton pump inhibitors and risk of colorectal cancer. Gut. 2022;71(1):111–8.
27. Lai SW, Liao KF, Lai HC, Lin CL, Sung FC. Use of proton pump inhibitors correlates with increased risk of colorectal cancer in Taiwan. Asia Pac J Clin Oncol [Internet]. 2013 Jun 1 [cited 2022 Aug 26];9(2):192–3. Available from: https://onlinelibrary.wiley.com/doi/full/10.1111/ajco.12054

For peer review only

28. Hwang IC, Chang J, Park SM. Emerging hazard effects of proton pump inhibitor on the risk of colorectal cancer in low-risk populations: A Korean nationwide prospective cohort study. PLoS One. 2017 Dec 1;12(12):e0189114.
29. Thrumurthy SG, Thrumurthy SSD, Gilbert CE, Ross P, Haji A. Colorectal adenocarcinoma: risks, prevention and diagnosis. BMJ [Internet]. 2016 Jul 14 [cited 2022 Oct 4];354. Available from: https://www-bmj-com.proxy.kib.ki.se/content/354/bmj.i3590
30. Baran B, Mert Ozupek N, Yerli Tetik N, Acar E, Bekcioglu O, Baskin Y. Difference Between Left-Sided and Right-Sided Colorectal Cancer: A Focused Review of Literature. Gastroenterology Res [Internet]. 2018 [cited 2020 Oct 29];11(4):264–73. Available from: /pmc/articles/PMC6089587/?report=abstract
31. Strand DS, Kim D, Peura DA. 25 Years of Proton Pump Inhibitors: A Comprehensive Review. Gut Liver [Internet]. 2017 Jan 1 [cited 2022 Oct 24];11(1):27. Available from: /pmc/articles/PMC5221858/
32. Socialstyrelsen. The National Patient Register - Socialstyrelsen [Internet]. 2019 [cited 2020 Oct 29]. Available from: https://www.socialstyrelsen.se/en/statistics-and-data/registers/register-information/the-national-patient-register/
33. Swedish Cancer Registry Quality [Internet]. Available from: https://www.socialstyrelsen.se/en/statistics-and-data/registers/register-information/swedish-cancer-register/
34. Ludvigsson JF, Almqvist C, Bonamy AKE, Ljung R, Michaëlsson K, Neovius M, et al. Registers of the Swedish total population and their use in medical research. Eur J Epidemiol. 2016 Feb 1;31(2):125–36.
35. Brooke HL, Talbäck M, Hörnblad J, Johansson LA, Ludvigsson JF, Druid H, et al. The Swedish cause of death register. Eur J Epidemiol [Internet]. 2017 Sep 5 [cited 2020 Oct 29];32(9):765–73. Available from: http://link.springer.com/10.1007/s10654-017-0316-1
36. Wallerstedt SM, Wettermark B, Hoffmann M. The First Decade with the Swedish Prescribed Drug Register – A Systematic Review of the Output in the Scientific Literature. Basic Clin Pharmacol Toxicol [Internet]. 2016 Nov 24 [cited 2020 Oct 29];119(5):464–9. Available from: https://onlinelibrary.wiley.com/doi/10.1111/bcpt.12613
37. von Elm E, Altman DG, Egger M, Pocock SJ, Gøtzsche PC, Vandenbroucke JP. The Strengthening the Reporting of Observational Studies in Epidemiology (STROBE) statement: guidelines for reporting observational studies. The Lancet [Internet]. 2007 Oct 20 [cited 2022 Jun 28];370(9596):1453–7. Available from: http://www.thelancet.com/article/S014067360761602X/fulltext
38. FASS General - Home [Internet]. [cited 2021 Feb 2]. Available from: https://www.fass.se/LIF/startpage
39. Barlow L, Westergren K, Holmberg L, Talbäck M. The completeness of the Swedish Cancer Register – a sample survey for year 1998. Acta Oncol (Madr) [Internet]. 2009 Jan 8 [cited 2020 Oct 30];48(1):27–33. Available from: http://www.tandfonline.com/doi/full/10.1080/02841860802247664
40. Vogel JD, Eskicioglu C, Weiser MR, Feingold DL, Steele SR. The American society of colon and rectal surgeons clinical practice guidelines for the treatment of colon cancer. Dis Colon Rectum. 2017;60(10):999–1017.
41. Brusselaers N, Lagergren J. Maintenance use of non-steroidal anti-inflammatory drugs and risk of gastrointestinal cancer in a nationwide population-based cohort study in Sweden. BMJ Open. 2018;8(7).

For peer review only

42. Machin D, Breslow NE, Day NE. Statistical Methods in Cancer Research, Vol. II: The Design and Analysis of Cohort Studies. Biometrics [Internet]. 1990 Dec;46(4):1243. Available from: https://www.jstor.org/stable/2532481?origin=crossref
43. Statistics database - National Board of Health and Welfare [Internet]. [cited 2024 Apr 2]. Available from: https://www.socialstyrelsen.se/statistik-och-data/statistik/statistikdatabasen/
44. Bender R. Introduction to the use of regression models in epidemiology. Methods in Molecular Biology. 2009;471:179–95.
45. Simon K. Colorectal cancer development and advances in screening. Clin Interv Aging. 2016;11:967–76.
46. Chan AT, Giovannucci EL, Meyerhardt JA, Schernhammer ES, Curhan GC, Fuchs CS. Long-term use of aspirin and nonsteroidal anti-inflammatory drugs and risk of colorectal cancer. JAMA [Internet]. 2005 Aug 1 [cited 2022 Sep 6];294(8):914–23. Available from: https://europepmc.org/articles/PMC1550973
47. Zhong L, Shanahan ER, Raj A, Koloski NA, Fletcher L, Morrison M, et al. Dyspepsia and the microbiome: time to focus on the small intestine. Vol. 66, Gut. England; 2017. p. 1168–9.
48. Minalyan A, Gabrielyan L, Scott D, Jacobs J, Pisegna JR. The Gastric and Intestinal Microbiome: Role of Proton Pump Inhibitors. Curr Gastroenterol Rep. 2017 Aug;19(8):42.
49. Macke L, Schulz C, Koletzko L, Malfertheiner P. Systematic review: the effects of proton pump inhibitors on the microbiome of the digestive tract-evidence from next-generation sequencing studies. Aliment Pharmacol Ther. 2020 Mar;51(5):505–26.
50. Robertson DJ, Sandler RS, Ahnen DJ, Greenberg ER, Mott LA, Cole BF, et al. Gastrin, Helicobacter pylori, and Colorectal Adenomas. Clinical Gastroenterology and Hepatology [Internet]. 2009 Feb [cited 2018 Dec 19];7(2):163–7. Available from: http://linkinghub.elsevier.com/retrieve/pii/S1542356508009415
51. Effects of three H2-receptor antagonists (cimetidine, famotidine, ranitidine) on serum gastrin level - PubMed [Internet]. [cited 2024 Apr 2]. Available from: https://pubmed.ncbi.nlm.nih.gov/12503773/
52. Baldwin GS, Shulkes A. Gastrin as an autocrine growth factor in colorectal carcinoma: implications for therapy. World J Gastroenterol [Internet]. 1998 Dec 12 [cited 2023 Sep 20];4(6):461. Available from: /pmc/articles/PMC4723428/
53. Babic A, Zhang X, Morales-Oyarvide V, Yuan C, Khalaf N, Khalili H, et al. Acid-suppressive medications and risk of colorectal cancer: results from three large prospective cohort studies. Br J Cancer [Internet]. 2020 Sep 9 [cited 2022 Aug 26];123(5):844–51. Available from: /pmc/articles/PMC7462971/
54. Population statistics [Internet]. [cited 2023 Nov 13]. Available from: https://www.scb.se/en/finding-statistics/statistics-by-subject-area/population/population-composition/population-statistics/
55. Brusselaers N, Wahlin K, Engstrand L, Lagergren J. Maintenance therapy with proton pump inhibitors and risk of gastric cancer: A nationwide population-based cohort study in Sweden. BMJ Open [Internet]. 2017 Oct 30 [cited 2019 Feb 7];7(10):e017739. Available from: http://www.ncbi.nlm.nih.gov/pubmed/29084798
56. Hálfdánarson Ó, Pottegård A, Björnsson ES, Lund SH, Ogmundsdottir MH, Steingrímsson E, et al. Proton-pump inhibitors among adults: a nationwide drug-utilization study. Therap Adv Gastroenterol. 2018;11:1–11.
57. Calvo LLJ, García Cámara P, Llorente Barrio M, Sierra Gabarda O, Monzón Baez R, Arbonés Mainar JM, et al. Successful deprescribing of proton pump inhibitors with a

For peer review only - http://bmjopen.bmj.com/site/about/guidelines.xhtml

patient-centered process: the DESPIBP Project. Eur J Clin Pharmacol [Internet]. 2021;77(12):1927–33. Available from: https://doi.org/10.1007/s00228-021-03186-x

58. Muheim L, Signorell A, Markun S, Chmiel C, Neuner-Jehle S, Blozik E, et al. Potentially inappropriate proton-pump inhibitor prescription in the general population: a claims-based retrospective time trend analysis. Therap Adv Gastroenterol [Internet]. 2021 Jun;14(6):1756284821998928. Available from: http://www.ncbi.nlm.nih.gov/pubmed/28086035

59. Brenner DR, Ruan Y, Shaw E, De P, Heitman SJ, Hilsden RJ. Increasing colorectal cancer incidence trends among younger adults in Canada. Prev Med. 2017 Dec 1;105:345–9.

60. Vuik FER, Nieuwenburg SAV, Bardou M, Lansdorp-Vogelaar I, Dinis-Ribeiro M, Bento MJ, et al. Increasing incidence of colorectal cancer in young adults in Europe over the last 25 years. Gut. 2019;

61. Potter JD. Rising rates of colorectal cancer in younger adults. BMJ [Internet]. 2019 Jun 24 [cited 2022 Sep 15];365. Available from: https://www.bmj.com/content/365/bmj.l4280

62. Meguid RA, Slidell MB, Wolfgang CL, Chang DC, Ahuja N. Is there a difference in survival between right- versus left-sided colon cancers? Ann Surg Oncol [Internet]. 2008 Sep [cited 2020 Oct 29];15(9):2388–94. Available from: https://pubmed.ncbi.nlm.nih.gov/18622647/

63. Mik M, Berut M, Dziki L, Trzcinski R, Dziki A. Right-and left-sided colon cancer-clinical and pathological differences of the disease entity in one organ. Archives of Medical Science [Internet]. 2017 Feb 1 [cited 2020 Oct 29];13(1):157–62. Available from: /pmc/articles/PMC5206358/?report=abstract

For peer review only

Figure 1. Duration of follow-up and risk of colorectal adenocarcinomas among (**a**) maintenance proton pump inhibitor (PPI) users and maintenance histamine-2 receptor antagonist (H_2RA) users (**b**) overall maintenance PPI users and users diagnosed with right-sided or left-sided colorectal adenocarcinomas, expressed as standardised incidence ratios (SIRs) and 95% confidence intervals (CIs)

For peer review only

Figure 1. Duration of follow-up and risk of colorectal adenocarcinomas among (**a**) maintenance proton pump inhibitor (PPI) users and maintenance histamine-2 receptor antagonist (H_2RA) users (**b**) overall maintenance PPI users and users diagnosed with right-sided or left-sided colorectal adenocarcinomas, expressed as standardised incidence ratios (SIRs) and 95% confidence intervals (CIs)

(**a**) maintenance PPI users and H_2RA users

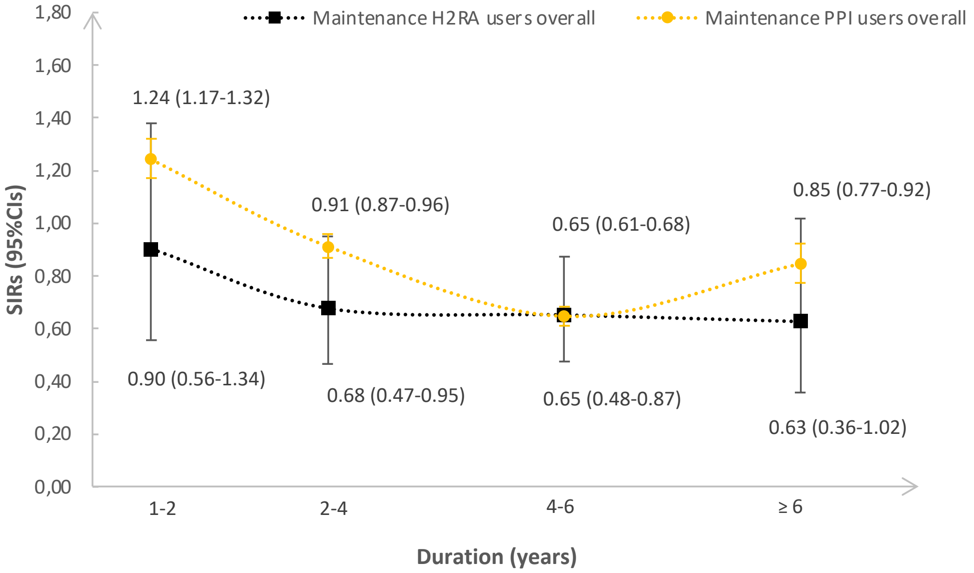

(**b**) overall maintenance PPI users and users diagnosed with right-sided or left-sided colorectal adenocarcinomas

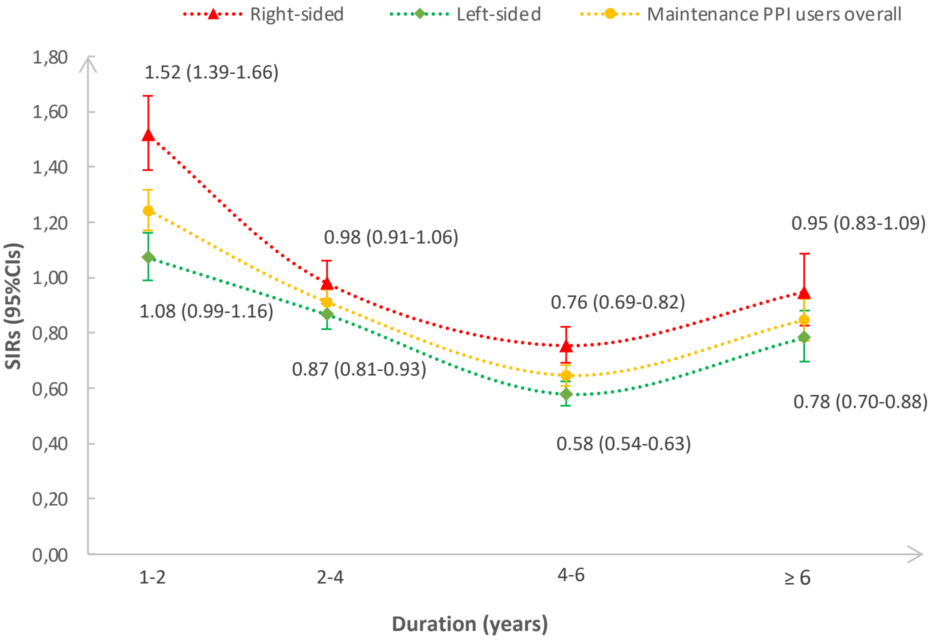

Supplementary Materials:

Table S1. International Classification of Diseases code for oncology and Anatomical Therapeutic Chemical codes used in this study

Table S2. Information of colorectal adenocarcinoma in the Swedish total population between 2005 and 2012

Table S3. Risk of colorectal adenocarcinomas in maintenance proton pump inhibitor (PPI) users followed up from enrolment, expressed as standardised incidence ratios (SIRs) and 95% confidence intervals (CIs) by age, sex, calendar period, tumour subsites and stages

Figure S1. Flow chart illustrating the enrolment of the study population of (**a**) maintenance proton pump inhibitor (PPI) users (**b**) maintenance histamine-2 receptor antagonist (H_2RA) users

Figure S2. Charts showing the crude incidence of Swedish total population.

For peer review only

Table S1. International Classification of Diseases code for oncology and Anatomical Therapeutic Chemical codes used in this study

Diseases	International Classification of Diseases code for oncology, 10th edition	Dispensed drugs	Anatomical Therapeutic Chemical code
Colorectal cancer	C18 -C20	Proton pump inhibitor	A02BC
Adenocarcinoma	096	Non-steroidal anti-inflammatory drug	M01A
Right-sided colorectal cancer	C18.0 -C18.4	Low-dose aspirin	B01AC06
Left-sided colorectal cancer	C18.5 - C18.7, C19, C20	Histamine-2 receptor antagonist	A02BA
Overlapping or unspecified sites of colorectal cancer	C18.8 C18.9		

For peer review only

Table S2. Information of colorectal adenocarcinoma in the Swedish total population between 2005 and 2012

Characteristics	Colorectal adenocarcinoma
Total	34,765
Age at entry the cohort, years	
18-39 years	532
40-49 years	1,324
50-59 years	3,974
60-69 years	9,298
≥70 years	19,637
Sex	
Men	18,601
Women	16,164
Calendar period	
2005-2006	7,998
2007-2009	13,044
2010-2012	13,723
Colorectal adenocarcinoma	
Right-sided	11,743
Left-sided	22,229
Overlapping or unspecified	793

For peer review only

Table S3. Risk of colorectal adenocarcinomas in maintenance proton pump inhibitor (PPI) users followed from enrolment, expressed as standardised incidence ratios (SIRs) and 95% confidence intervals (CIs) by age, sex, calendar period, tumour subsites and stages

	Maintenance PPI users with maximum follow-up time	
	Number	SIRs (95% CI)
Total colorectal adenocarcinomas	6,887	1.44 (1.40-1.47)
Sex		
Men	3,341	1.48 (1.43-1.53)
Women	3,546	1.40 (1.35-1.44)
Age at starting the maintenance PPI use, years		
18-39	67	5.53 (4.03-7.39)
40-49	218	2.75 (2.35-3.19)
50-59	775	1.78 (1.64-1.94)
60-69	1,870	1.60 (1.52-1.68)
≥70	3,957	1.32 (1.28-1.36)
Calendar year period		
2005-2006	4,126	1.76 (1.65-1.88)
2007-2009	1,982	1.55 (1.49-1.60)
2010-2012	779	1.28 (1.24-1.33)
Subsites of colorectal adenocarcinoma		
Right-sided	3,043	1.74 (1.68-1.81)
Left-sided	3,710	1.25 (1.21-1.29)
Stages of colorectal adenocarcinoma		
Stage 0-I	966	1.49 (1.40-1.59)
Stage II	1,657	1.41 (1.34-1.48)
Stage III	1,561	1.40 (1.33-1.47)
Stage IV	1,172	1.27 (1.19-1.34)
Missing	1,531	1.62 (1.54-1.70)
Follow-up time, years (median)	6.1	-

Figure S1. Flow chart illustrating the enrolment of the study population of (**a**) maintenance proton pump inhibitor (PPI) users (**b**) maintenance histamine-2 receptor antagonist (H_2RA) users

(**a**) maintenance PPI users

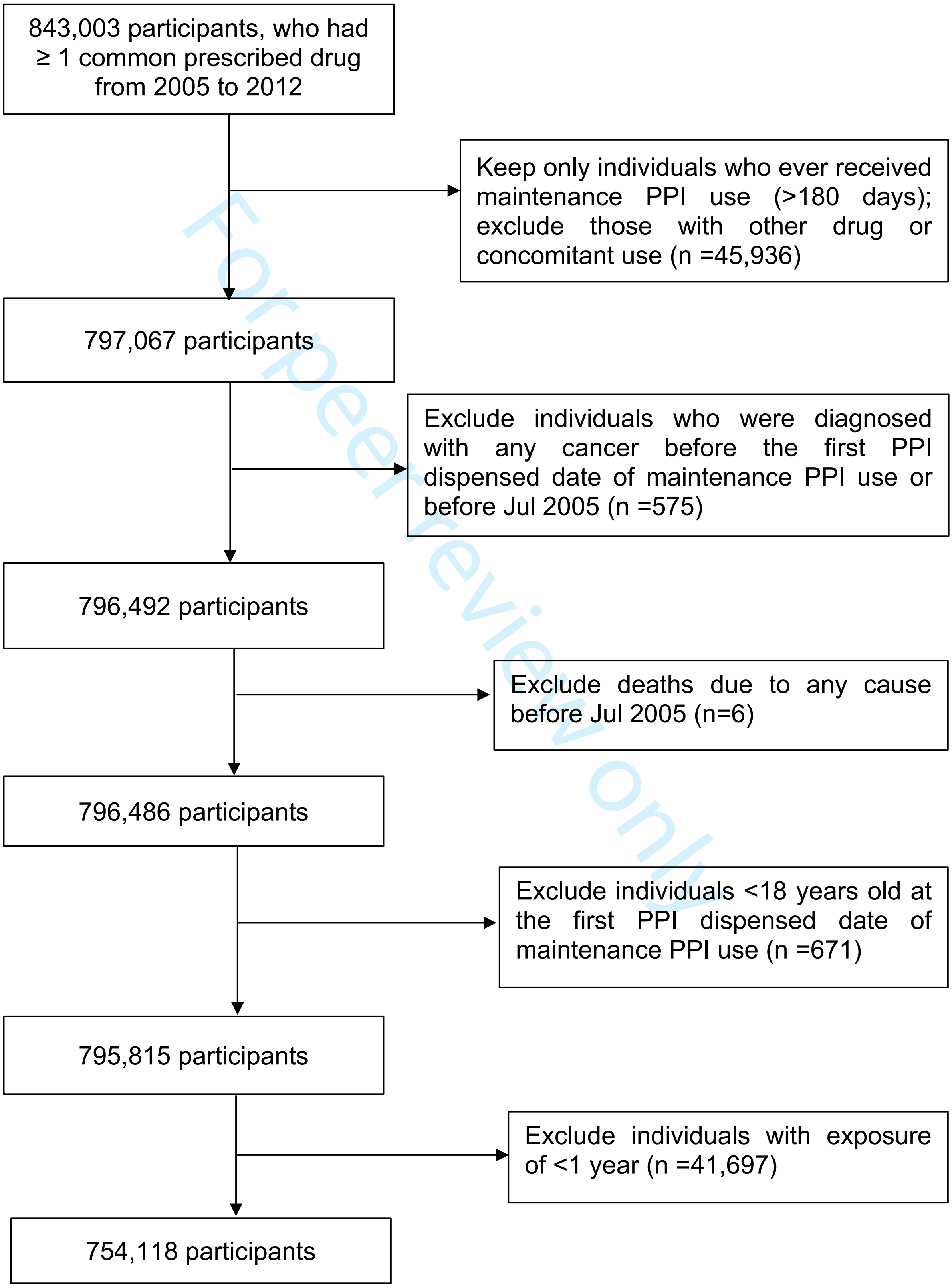

For peer review only

(**b**) maintenance H_2RA users

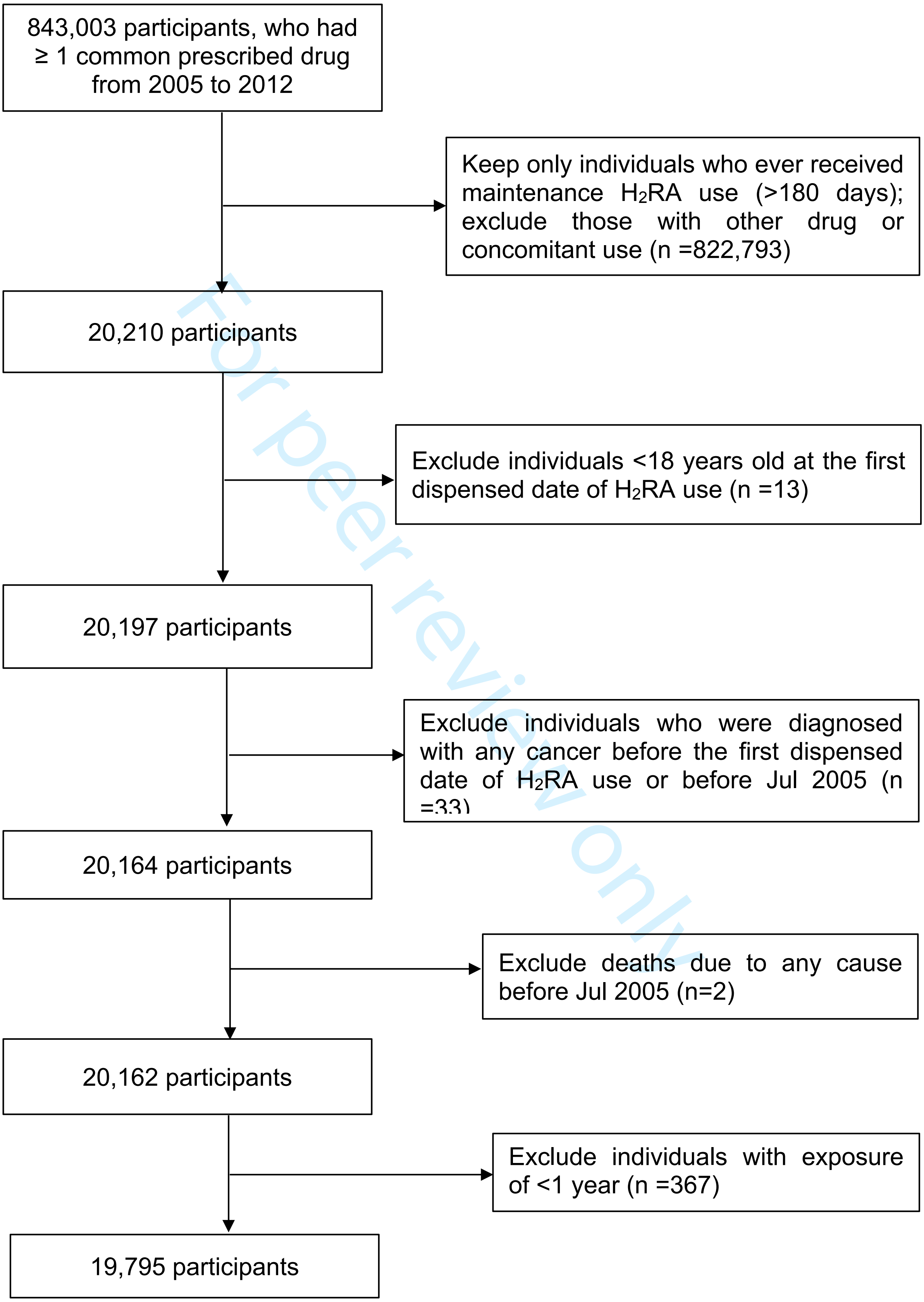

For peer review only

Figure S2. Charts showing the crude incidence of Swedish total population. Data were retrieved from the National Board of Health and Welfare, Statistical Database.

(**a**) Crude incidence of new colorectal adenocarcinoma cases (over 20 years old) in Sweden (2012), by sex

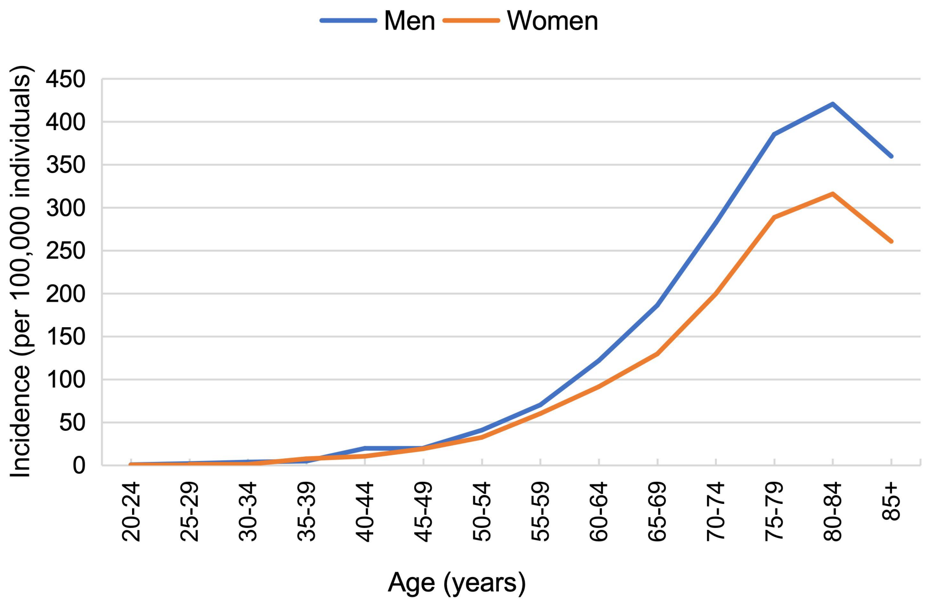

(**b**) Crude incidence of new colorectal adenocarcinoma cases in Sweden (2006-2012), by sex

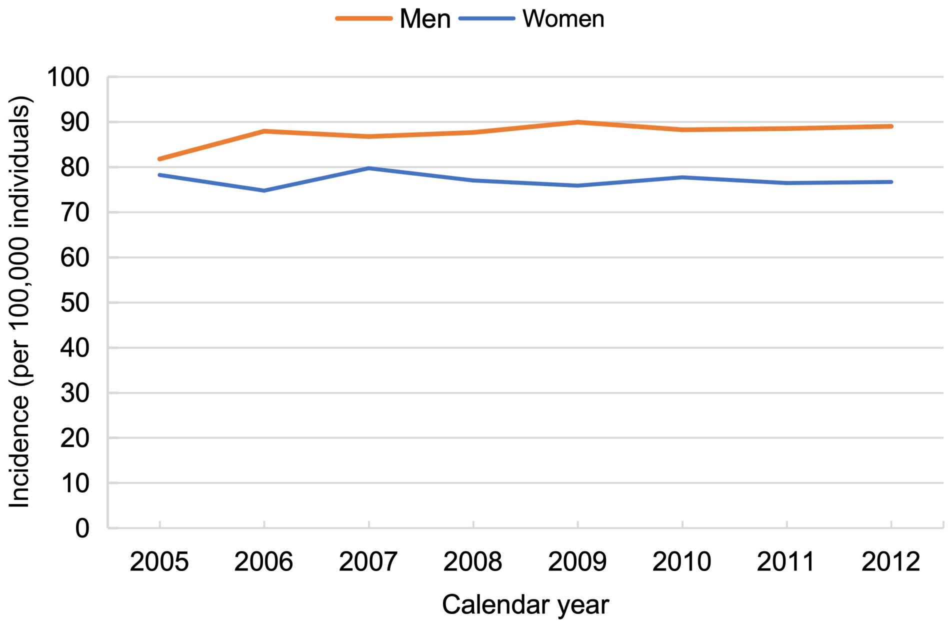

STROBE Statement—Checklist of items that should be included in reports of ***cohort studies***

	Item No	Recommendation	Page No
**Title and abstract**	1	(*a*) Indicate the study's design with a commonly used term in the title or the abstract<br>(*b*) Provide in the abstract an informative and balanced summary of what was done and what was found	1,2
**Introduction**			
Background/rationale	2	Explain the scientific background and rationale for the investigation being reported	4
Objectives	3	State specific objectives, including any prespecified hypotheses	4
**Methods**			
Study design	4	Present key elements of study design early in the paper	5
Setting	5	Describe the setting, locations, and relevant dates, including periods of recruitment, exposure, follow-up, and data collection	5
Participants	6	(*a*) Give the eligibility criteria, and the sources and methods of selection of participants. Describe methods of follow-up<br>(*b*) For matched studies, give matching criteria and number of exposed and unexposed	5
Variables	7	Clearly define all outcomes, exposures, predictors, potential confounders, and effect modifiers. Give diagnostic criteria, if applicable	6
Data sources/ measurement	8*	For each variable of interest, give sources of data and details of methods of assessment (measurement). Describe comparability of assessment methods if there is more than one group	5
Bias	9	Describe any efforts to address potential sources of bias	3,4,5
Study size	10	Explain how the study size was arrived at	5
Quantitative variables	11	Explain how quantitative variables were handled in the analyses. If applicable, describe which groupings were chosen and why	5,6
Statistical methods	12	(*a*) Describe all statistical methods, including those used to control for confounding<br>(*b*) Describe any methods used to examine subgroups and interactions<br>(*c*) Explain how missing data were addressed<br>(*d*) If applicable, explain how loss to follow-up was addressed<br>(*e*) Describe any sensitivity analyses	6,7
**Results**			
Participants	13*	(a) Report numbers of individuals at each stage of study—eg numbers potentially eligible, examined for eligibility, confirmed eligible, included in the study, completing follow-up, and analysed<br>(b) Give reasons for non-participation at each stage<br>(c) Consider use of a flow diagram	S1
Descriptive data	14*	(a) Give characteristics of study participants (eg demographic, clinical, social) and information on exposures and potential confounders<br>(b) Indicate number of participants with missing data for each variable of interest<br>(c) Summarise follow-up time (eg, average and total amount)	7-15
Outcome data	15*	Report numbers of outcome events or summary measures over time	7-15

For peer review only

For peer review only - http://bmjopen.bmj.com/site/about/guidelines.xhtml

Main results	16	(*a*) Give unadjusted estimates and, if applicable, confounder-adjusted estimates and their precision (eg, 95% confidence interval). Make clear which confounders were adjusted for and why they were included<br>(*b*) Report category boundaries when continuous variables were categorized<br>(*c*) If relevant, consider translating estimates of relative risk into absolute risk for a meaningful time period	7-15
Other analyses	17	Report other analyses done—eg analyses of subgroups and interactions, and sensitivity analyses	15
**Discussion**			
Key results	18	Summarise key results with reference to study objectives	15
Limitations	19	Discuss limitations of the study, taking into account sources of potential bias or imprecision. Discuss both direction and magnitude of any potential bias	17
Interpretation	20	Give a cautious overall interpretation of results considering objectives, limitations, multiplicity of analyses, results from similar studies, and other relevant evidence	15,16
Generalisability	21	Discuss the generalisability (external validity) of the study results	17
**Other information**			
Funding	22	Give the source of funding and the role of the funders for the present study and, if applicable, for the original study on which the present article is based	18

*Give information separately for exposed and unexposed groups.

Note: An Explanation and Elaboration article discusses each checklist item and gives methodological background and published examples of transparent reporting. The STROBE checklist is best used in conjunction with this article (freely available on the Web sites of PLoS Medicine at http://www.plosmedicine.org/, Annals of Internal Medicine at http://www.annals.org/, and Epidemiology at http://www.epidem.com/). Information on the STROBE Initiative is available at http://www.strobe-statement.org.

For peer review only
